# Structure-Based Design of Promysalin Analogues to Overcome Mechanisms of Bacterial Resistance

**DOI:** 10.1021/acsomega.3c00884

**Published:** 2023-03-22

**Authors:** Andrew R. Mahoney, Kelly M. Storek, William M. Wuest

**Affiliations:** † Department of Chemistry, Emory Univers ity, and Emory Antibiotic Resistance Center, Emory University School of Medicine, Atlanta, Georgia 30322, United States; ‡ Department of Infectious Diseases, Genentech, Inc. South San Francisco, California 94080, United States

## Abstract

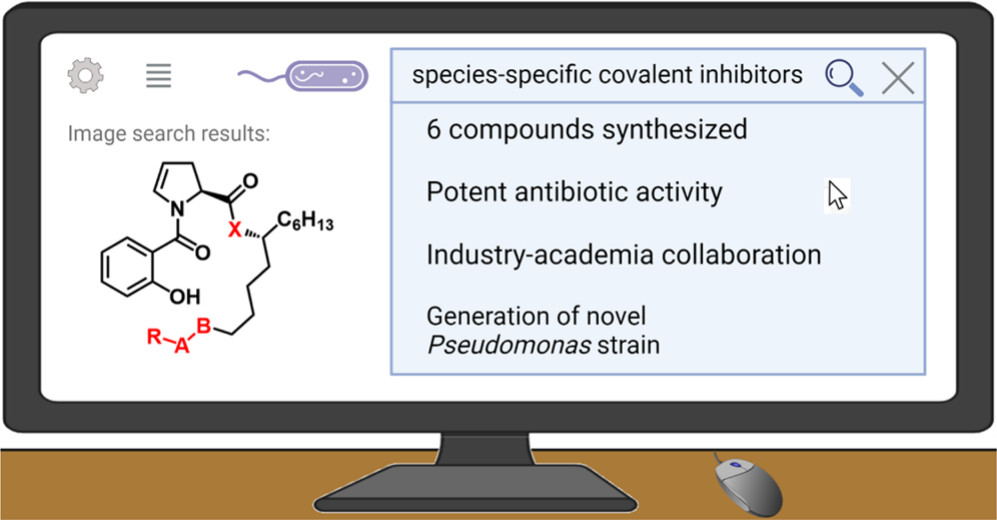

The search for antibiotics that function through novel mechanisms of action is ongoing, and recent progress in our lab identified the tricarboxylic acid cycle as a viable option. Promysalin is a secondary metabolite capable of species-specific inhibition of *Pseudomonas aeruginosa*, a common opportunistic pathogen. Promysalin disrupts primary metabolism in this bacterium by competitively inhibiting succinate dehydrogenase at the ubiquinone binding site. However, the activity of promysalin in cellulo is marred potentially by its chemical instability and/or propensity for efflux. To assess the success of these novel analogues, a novel strain of *P. aeruginosa* harboring gene deletions of eight efflux pumps and porins was developed and implemented. Herein, we disclose the synthesis and biological investigation of six promysalin analogues to overcome these liabilities and demonstrate that efflux likely plays a significant role in tolerating the effect of the inhibitor.

## Introduction

The continuous rise in the prevalence of drug-resistant pathogens presents an ever-growing challenge for society. According to the Centers for Disease Control’s 2019 Antibiotic/Antimicrobial Resistance Threats Report, over 3 million infections caused by antimicrobial-resistant bacteria and fungi occur in the United States each year, resulting in 48,000 deaths annually.^1^ The financial burden of antimicrobial resistance is also extensive, costing an estimated $55 billion annually for healthcare and lost productivity.^2^ The onset of the COVID-19 pandemic has only worsened the issue, reversing the progress made in addressing underlying causes of the resistance crisis such as overprescription (about 80% of patients hospitalized with COVID-19 were prescribed a prophylactic antibiotic) and misuse of antibiotics, leading to a further 15% increase in antimicrobial-resistant infections and deaths in 2020.^3−5^ Antimicrobial resistance also disproportionately affects developing countries, associated with a global death toll of over 5 million people in 2019 alone.^6^ The high costs associated with antibiotic development, short clinical lifespan of such drugs, and increased regulatory challenges in the United States have led to many American pharmaceutical companies downsizing or shuttering their antibiotic research and development programs.

Several strategies have been proposed to mitigate or reverse the antimicrobial resistance crisis. Development of antibacterials and antifungals functioning through novel mechanisms of action can prevent cross-resistance and extend the useful clinical lifespan of these drugs.^7,8^ Resistance mechanisms can be directly targeted in combination therapies to potentiate the activities of currently used antibiotic compounds.^9,10^ Finally, advancements in diagnostic technologies to both quickly and accurately identify infectious pathogens can be coupled with the development of selective, narrow-spectrum antibiotics.^11^ These narrow-spectrum antibiotics are enticing, as they do not disrupt patients’ commensal microbiota and generate less selective pressure for pan-resistance development.

Natural products serve as a plentiful pool of structurally diverse small molecules from which new antibiotics can be developed. Between 1994 and 2014, over a third of the 1,562 FDA-approved drugs were either natural products or derivatives thereof.^12^ This statistic becomes even more impressive when examining only antimicrobial and antitumor drugs, of which an estimated 50–70% are derived from natural product scaffolds.^13^ The use of natural products for drug development is logical, capitalizing on the fact that bacteria exist as complex communities in their natural environments, consisting of many diverse species. Competing for the limited resources around them, these bacteria and other microorganisms constantly utilize intricate biological machinery to rapidly synthesize structurally complex natural products to inhibit the growth of neighboring species.^14−16^

Our group has been interested in the *Pseudomonas putida* (*PP*) secondary metabolite promysalin, which exhibits potent (IC_50_ = 67 nM) antibiotic activity against *Pseudomonas aeruginosa* (*PA*), a common Gram-negative opportunistic pathogen recently identified as a “serious threat” by the CDC.^2^ Promysalin was isolated in 2011 from the rhizosphere of a rice plant, a complex multispecies environment that encourages upregulation of antibiotic-producing biosynthetic pathways.^17^

In addition to completing the first total synthesis of promysalin and elucidating its stereochemistry, our group has used affinity-based protein profiling techniques and resistance selection assays to demonstrate that promysalin functions via competitive inhibition of succinate dehydrogenase by serving as a ubiquinone mimic.^18,19^ The fact that species-specific bacterial inhibition can be derived from targeting a conserved enzyme in primary metabolism is surprising but not unprecedented, as the fungal secondary metabolite siccanin has also been shown to selectively inhibit succinate dehydrogenase in *Pseudomonas* species, and in fact, fungicides which selectively target succinate dehydrogenase, have been in use since the 1960s.^20,21^

**Figure 1:**
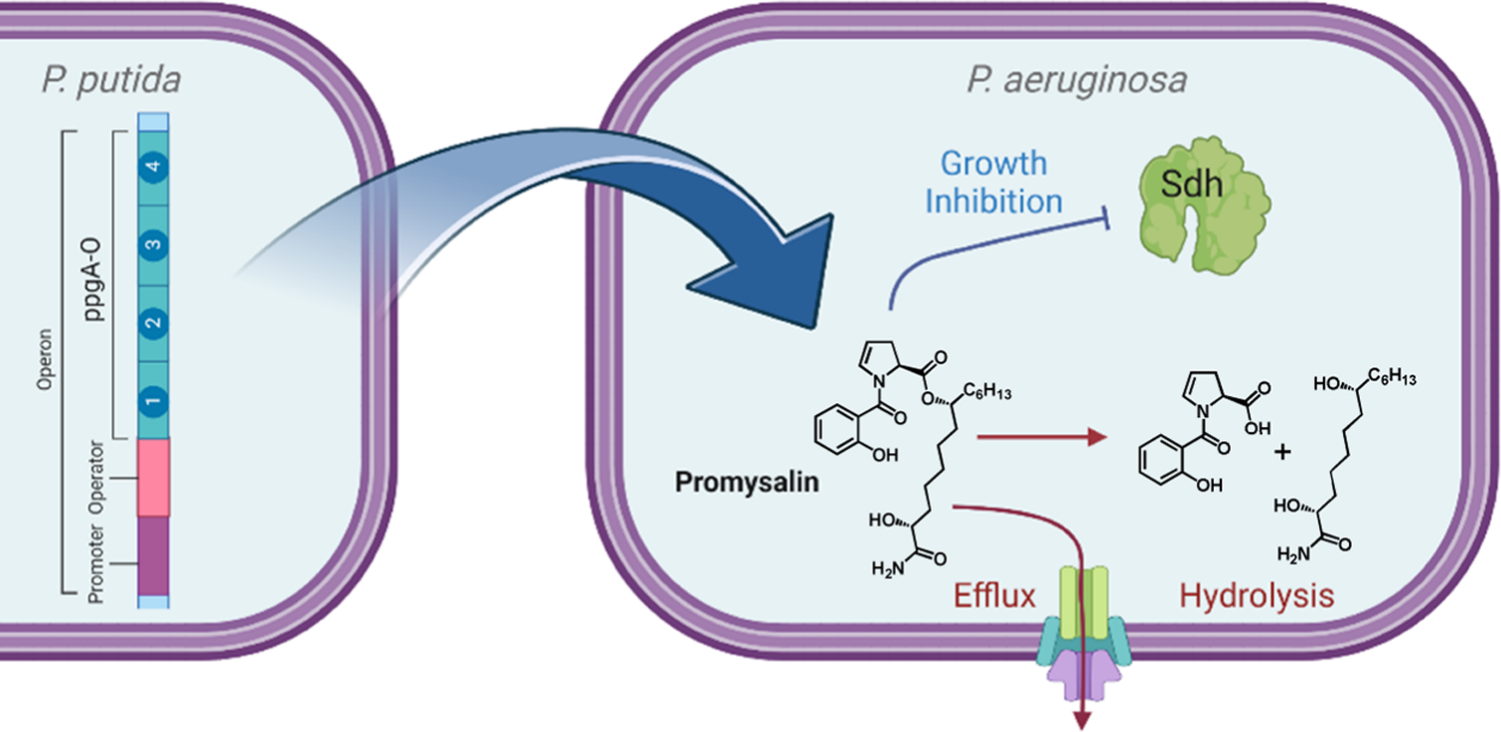
Promysalin is a secondary metabolite produced by *P. putida* to selectively inhibit the growth of closely related *P. aeruginosa* by binding succinate dehydrogenase (Sdh). *P. aeruginosa* is hypothesized to utilize hydrolysis or efflux strategies to resist the effects of this antibiotic.

While promysalin has promising *PA* inhibition results, the large difference between its IC_50_ and its minimum inhibitory concentration (MIC) suggests some form of intrinsic resistance in *PA* populations. Despite an extensive structure–activity relationship (SAR) campaign, no synthetic analogue our group has generated thus far has been able to overcome this resistance and allow for complete bacterial inhibition at sufficiently low promysalin concentrations.^22^ We hypothesize that *PA*’s resistance and/or tolerance to promysalin may be occurring by (at least) one of two potential mechanisms (Figure 1). The hydrolytically labile ester linkage between the proline–salicylate ring system of promysalin and its aliphatic side chain may be cleaved enzymatically (by a yet-unidentified hydrolase) or nonenzymatically (by a stress-mediated change in intracellular pH). Alternatively, promysalin may be a substrate for one of many known specific or multidrug efflux pumps in *Pseudomonas*, decreasing its intracellular concentration to sublethal levels. In the producing strain of *PP*, four genes encoding efflux transporters are upregulated in the presence of exogenous promysalin.^23^

Herein, we report the rational design of six synthetic promysalin analogues to probe these two hypothesized mechanisms of resistance.

## Materials and Methods

### Synthesis Instrumentation and General Notes

NMR spectra were obtained using the following spectrometers: Varian INOVA 600 (600/150 MHz), Varian INOVA 500 (500/125 MHz), or Varian INOVA 400 (400/100 MHz). Chemical shifts are in ppm relative to TMS and use the indicated solvent as an internal reference. The following abbreviations are used to describe signal multiplicities: s (singlet), d (doublet), t (triplet), q (quartet), m (multiplet), br (broad), dd (doublet of doublets), dt (doublet of triplets), etc. Accurate mass spectra were recorded on a Thermo Scientific Exactive Plus Orbitrap MS.

Nonaqueous reactions were performed under an atmosphere of argon, in flame-dried glassware, with HPLC-grade solvents dried by passage through activated alumina. 2,6-lutidine, triethylamine, and diisopropylethylamine were freshly distilled from CaH_2_ prior to use. Brine refers to a saturated aqueous solution of sodium chloride, sat. NaHCO_3_ refers to a saturated aqueous solution of sodium bicarbonate, sat. NH_4_Cl refers to a saturated aqueous solution of ammonium chloride, etc. 3Å molecular sieves were activated via storage in a 120 °C oven and flame-dried under vacuum before use. “Column chromatography” refers to purification in a normal-phase gradient on a Biotage flash chromatography purification system. Metathesis catalysts were obtained as generous gifts from Materia, Inc. All other chemicals were used as received from Oakwood, TCI America, Sigma-Aldrich, Alfa Aesar, Ambeed, Combi-Blocks, or AK Scientific. All synthetic analogues undergoing biological testing were purified to >95% purity by HPLC using a gradient of 5–95% ACN/H2O.

### Bacterial Strains and Culture Conditions

*P. aeruginosa* PAO1 and PA14 were gifts from Prof. O’Toole (Dartmouth University). Bacterial cultures were grown from freezer stocks overnight (16–24 h) with shaking at 37 °C in Tryptic soy broth (TSB) media (10 mL). Growth curves were obtained for PA strains to determine the optical density (OD) of each strain in exponential growth; OD readings at a wavelength of 600 nm were taken every 10 min for 6 h in a plate reader at 37 °C with shaking and repeated six times. This data was used without alteration for this report. *P. aeruginosa* PA14 allelic replacement strains were constructed using an unmarked, nonpolar deletion strategy.^7,8^ Efflux deletion mutants were individually constructed and sequentially constructed to create an efflux null strain missing 8 efflux systems beginning with mexXY followed by mexCD-oprJ, mexJK, opmH, mexEF-oprN, oprD, mexGHI-opmD, and mexAB, respectively. To create each suicide vector, gBlocks containing 500–1000 bp upstream and downstream of the genes of interest, removing the entire coding region, were amplified using primers UP and DN primer sets (Table S1). The resultant PCR products were cloned into the suicide vector, pEX100T, via Gibson Assembly (New England BioLabs) recombination according to the manufacturer’s protocols. The resultant plasmid was verified by sequence analysis (ELIM Biopharm) and transformed into the conjugation-competent auxotroph, S17-1 ΔhemA cells supplemented with 50 μg mL^–1^ 5-aminolevulinic acid.^9^ The suicide vector was introduced into the strain of interest via conjugation.^8^ Single cross-over mutants were selected on LB containing 10 μg mL^–1^ gentamicin or a lower concentration as efflux mutants were created. Unmarked, double cross-over mutants were selected on LB without NaCl plates containing 10% sucrose and confirmed by PCR and sequence analysis.

### IC_50_ Assay

Compounds were serially diluted in sterile DI water from a stock solution (1 mM in 10% DMSO/90% H_2_O) to yield 24 test concentrations. Overnight cultures were diluted 1:100 in 5 mL of fresh media and grown with shaking at 37 °C to an OD reflecting exponential growth. Bacteria were diluted to an optical density of 0.004 using the following equation: (*x* μL bacterial culture)(OD reading) = (0.004)(volume needed) and 100 μL was inoculated into each well of a flat-bottom 96-well plate (Corning 3370) containing 100 μL of compound solution. Plates were incubated statically at 37 °C for 24 h, upon which time the OD at 595 nm was measured using a plate reader. IC_50_ values were calculated by fitting the OD readings vs concentration with a 4-parameter logistic model. Controls were prepared by serially diluting a 10% DMSO/90% H_2_O the same as the compound stock solution. Compounds were tested in triplicate from separate cultures, and results were averaged (Table S2).

## Results

**Figure 2:**
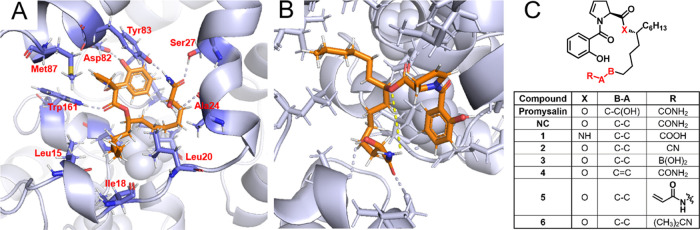
Computational docking model of promysalin in the active site of Sdh in *PA*. (A) Identification of the primary residues comprising the promysalin binding site. (B) Predicted stabilizing intramolecular hydrogen bond between promysalin’s linker ester and terminal amide. (C) Structure of promysalin, known dehydroxylated analogue (−)-**NC**, and desired computationally inspired analogues.

Previous SAR investigations by our group have indicated the immutability of the proline–salicylate ring system of promysalin and have highlighted that the myristic acid-derived aliphatic side chain is much more amenable to analogue design.^22^ We were therefore interested in the investigation of the characteristics of the *PA* Sdh ubiquinone binding site adjacent to the anticipated localization of promysalin’s side chain (Figure 2). Due to the lack of an available crystal structure of *PA* Sdh, we utilized a homology model of the enzyme derived from *Escherichia coli* generated by the Karanicolas group for structural guidance.^24^

Docking studies of promysalin in the ubiquinone binding site of this model reveal a nonpolar pocket composed of alanine, leucine, and isoleucine residues, which presumably stabilize the aliphatic side chain of promysalin through the hydrophobic effect. Additionally, hydrogen-bonding interactions to the nearby Tyr83, Ser27, and backbone of Ala24 are predicted to stabilize the amide terminus of promysalin’s side chain (Figure 2A). Due to the twisted conformation of promysalin’s side chain in the docking model, there also exists the possibility for an intramolecular hydrogen-bonding interaction between one of the amide protons and the oxygen of the linker ester (Figure 2B). This hydrogen bond would provide additional conformational rigidity to the sp^3^-rich molecule, allowing for more ordered and higher affinity binding.

With the knowledge that the side chain of promysalin is amenable to analogue design, we sought to leverage the amino acid residues nearest the amide terminus to engage in a covalent interaction. Such targeted covalent inhibitor drugs have seen a resurgence in recent years, as methods to understand and tune their reactivities have been developed and allow for more potent and selective protein inhibitors. We therefore envisioned replacing the amide functional group on promysalin’s side chain with a variety of electrophilic moieties, which have shown success in pharmaceutical applications, namely, an acrylamide, a boronic acid, and a nitrile.^25,26^ By covalently ligating our small molecule drug to its protein target, we rationalized that the bacterium would be rendered unable to effectively efflux promysalin without degrading this linkage. We also sought to mitigate the potential PA resistance mechanism of ester hydrolysis by inverting the functionalities of the linker ester and terminal amide to create a carboxylic acid analogue linked by an internal amide, maintaining the potential intramolecular hydrogen-bonding interaction while increasing hydrolytic stability (Figure 2C).

Synthesis of the amide-methyl ester side chain required for **1** began with a precedented asymmetric Keck allylation of heptanal to deliver homoallylic alcohol **7** in high yield and enantioselectivity. Grubbs-catalyzed olefin metathesis of the resulting terminal alkene with methyl 5-hexenoate then generated intermediate **8**, possessing the required carbon framework. After significant optimization of reaction and purification conditions, Mitsunobu reaction with diphenylphosphoryl azide was successful in the conversion of this chiral alcohol to a chiral azide with clean inversion of stereochemistry. Catalytic hydrogenation of this azide and the internal olefin then delivered the required side chain **1a** for our desired amide acid analogue.

**Scheme 1:**
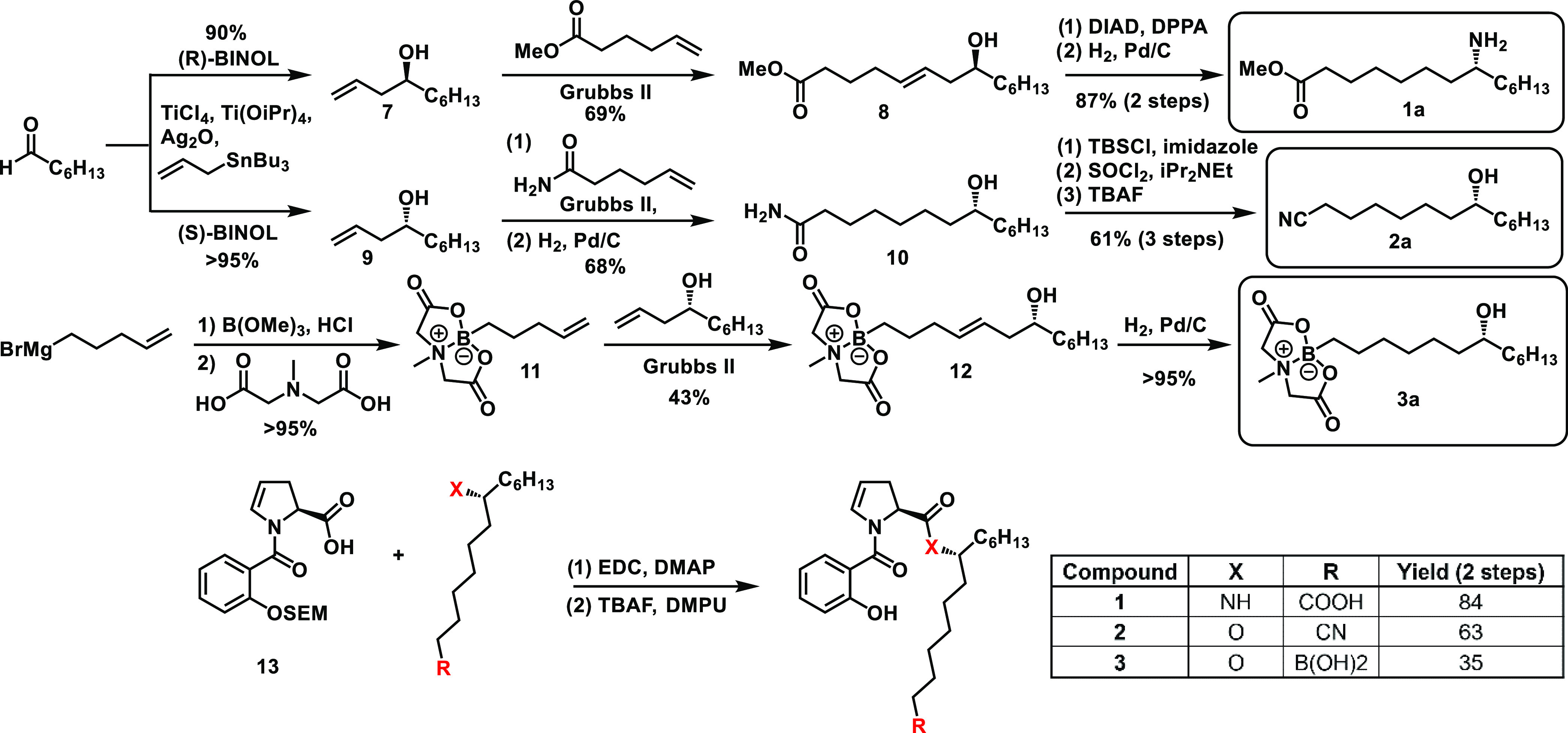
Initial Keck allylation Strategy to Access Analogues **1–3** Due to concerns of functional group compatibility, the order of 1-Ethyl-3-(3-dimethylaminopropyl)carbodiimide (EDC) coupling and tetrabutylammonium flouride (TBAF) deprotection steps for analogue **3** was inverted.

In a similar manner, the opposite enantiomer of our homoallylic alcohol (compound **9**) was used in cross-metathesis with 5-hexenamide, followed by hydrogenation to give 8-hydroxymyristamide **10**. Silyl protection of the chiral alcohol was necessary to prevent its chlorination or elimination during thionyl chloride-mediated dehydration of the terminal amide; deprotection of the silyl group then cleanly afforded the desired side chain **2a** (protection prior to olefin metathesis significantly lowered metathesis yield, presumably due to the high steric bulk near the reactive alkene center) (Scheme 1).

The successful regulatory approval of boron-containing drugs such as ixazomib, vaborbactam, and crisaborole is a relatively new phenomenon spurred on by the development of new synthetic methods to incorporate boron functional groups into organic compounds.^26^ Boron’s innate electrophilicity due to its vacant p-orbital renders it an excellent option for medicinal covalent inhibition, as well as a potential synthetic liability. Fortunately, the development of a robust and general boronic acid protecting group in the form of *N*-methyliminodiacetic acid (MIDA) boronates presented a useful solution to our synthetic concerns.^27^ MIDA protection of 4-pentenylboronic acid (generated by Grignard addition of 4-pentenylmagnesium bromide to trimethylborane) enabled successful cross-metathesis and hydrogenation to afford our desired protected boronic acid side chain **3a**.

**Scheme 2:**
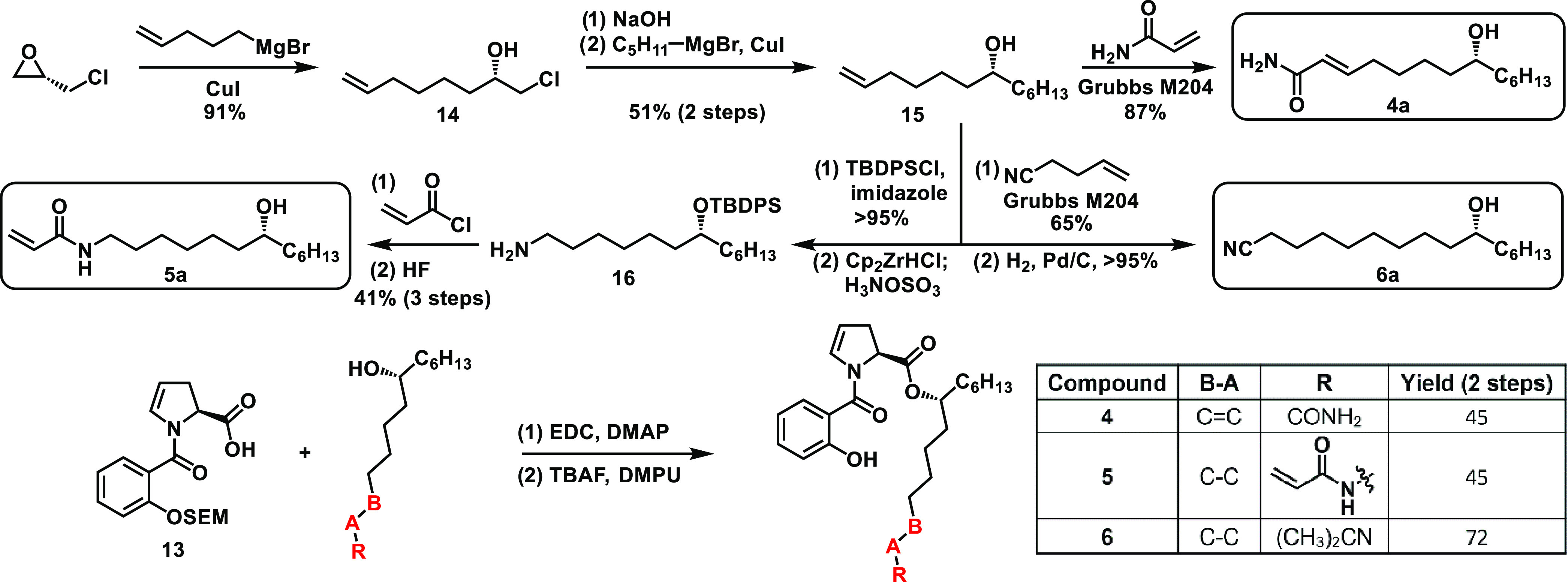
Sequential Grignard addition strategy to access analogues **4–6**

While asymmetric Keck allylation had proved successful in the formation of our chiral homoallylic alcohol intermediates, the arduous reaction setup and purification, as well as the high cost and high toxicity of the reagents required, led us to desire a more chemist-friendly approach. After considering several options, including alternative allylation reactions and chiral reductions, we settled on a sequential Grignard addition approach to commercially available (*S*)-epichlorohydrin.^28^ Regioselective copper-catalyzed Grignard addition of 5-pentenylmagnesium bromide to the less hindered face of this chiral epoxide proceeded in high yield. Base-mediated reformation of a terminal epoxide followed by second Grignard addition of pentylmagnesium bromide successfully generated chiral intermediate **(+)-15**, which we envisioned could be derivatized to form the rest of our desired analogues (Scheme 2).

For example, use of acrylamide in cross-metathesis generated side chain **4a**, whereas use of homoallyl cyanide followed by careful chemoselective hydrogenation of the internal olefin generated nitrile side chain **6a**. Alternatively, protection of the alcohol followed by anti-Markovnikov hydrozirconation amination using Schwartz’s reagent afforded terminal amine **16**, which could then be acylated with acryloyl chloride and O-deprotected to give the last of our desired analogue side chain, **5a**.

In general, EDC coupling of the promysalin analogue side chains to the corresponding proline–salicylate fragment followed by 2-(Trimethylsilyl)ethoxymethyl (SEM) deprotection with TBAF was straightforward. In the case of our boronic acid analogue, the incompatibility of MIDA boronates with hard nucleophiles such as fluoride necessitated SEM deprotection prior to EDC coupling. Rapid boronate deprotection was then achieved under aqueous basic conditions. In the case of the amide-linked analogue, a final base-mediated saponification was necessary to afford amide acid analogue (−)-**1**.

**Figure 3:**
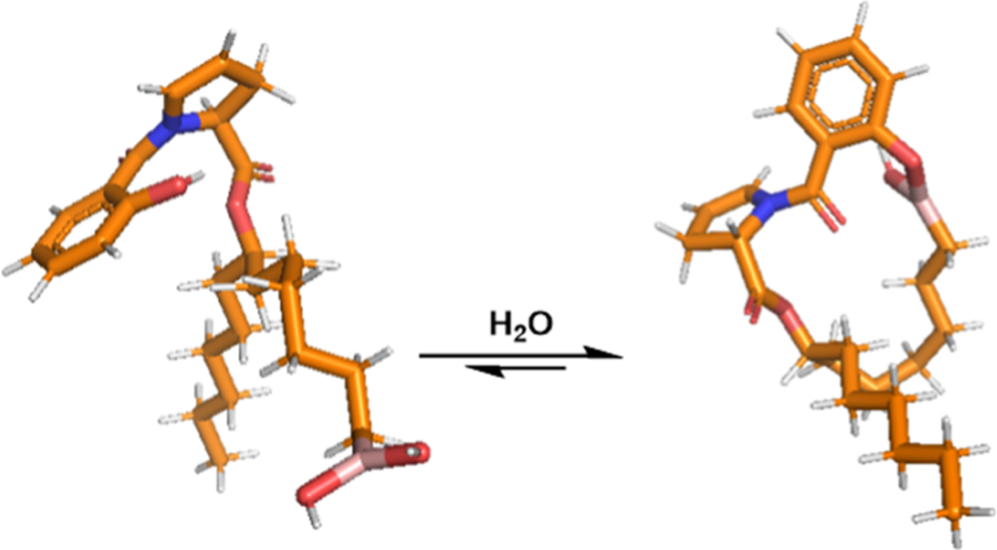
Reversible macrocyclization of boronic acid analogue (−)-**3**.

Armed with this small array of analogues, we turned our attention to biological analysis. All analogues were screened in inhibition assays against *PA* strains PA14 and PAO1, alongside gentamicin and a deoxy-promysalin, a previously reported noncovalent promysalin analogue termed (−)-**NC**, as positive controls (Table 1). As a preface, it should be noted that even at high concentrations (>100 μM) of our best-in-class promysalin analogue, we never observed complete growth inhibition for any of the *PA* strains tested (Figure S1); therefore, we report IC_50_ values as a proxy for activity. Of the newly synthesized analogues, nitrile analogue (−)-**2** proved most potent, with IC_50_ values of 1.58 and 2.04 μM against PA14 and PAO1, respectively. Extended analogues (−)-**5** and (−)-**6** both proved equipotent to their shortened counterparts, indicating a lack of steric or electronic constraints in that sector of the Sdh binding pocket. Interestingly, amide acid analogue (−)-**1** was completely inactive against either strain. One potential explanation for this result is that increased conformational strain about the amide linker relative to that of the ester-containing analogues and natural product prevents these molecules from adopting the twisted conformation observed in our docking model. Boronic acid analogue (−)-**3** also saw drastically decreased activity relative to the other analogues; this may be due to an observed reversible macrocyclization resulting from nucleophilic addition of the salicylate phenol into the boronic acid, confirmed by variable temperature NMR and mass spectrometry studies (Figure 3). As we have previously identified that the salicylate phenol is crucial for biological activity, this macrocyclization may be deleterious and sufficiently decrease Sdh binding affinity to result in the observed reduced *PA* inhibition.

**Table 1:** IC_50_ Data (in μM) for Promysalin Analogues as well as Previously Published Noncovalent Analogue (NC) Used as Positive Control, All Tested against Three *PA* Strains

analogue	PA14 (μM)	PAO1 (μM)	PA14 efflux knockout (μM)
**1**	>250	>250	not tested
**2**	1.58	2.04	2.67 × 10^–1^
**3**	21.6	42.2	not tested
**4**	5.24	15.2	2.99 × 10^–3^
**5**	5.58	7.69	3.88 × 10^–1^
**6**	3.67	2.56	1.28
**gentamicin**	6.16	4.90	3.50
**NC**	5.16 × 10^–1^	3.29	7.44 × 10^–5^

*PA* infections are difficult to successfully eradicate partly due to the organism’s inherent resistance to antibiotics. This trait is due to a combination of low membrane permeability and expression of multiple multidrug resistance (MDR) efflux pumps and porins. This is exemplified in clinical *PA* isolates from cystic fibrosis patients, which typically contain multiple genomic mutations that increase efflux activity. To assess whether our targeted covalent inhibitor-containing analogues were less susceptible to the hypothesized efflux mechanism of *PA* promysalin resistance, we tested analogues (−)-**2**, (−)-**4**, (−)-**5**, and (−)-**6** against a *PA* strain, harboring gene deletions of eight efflux pumps and porins (*mexXY*, *mexCD-oprJ*, *mexJK*, *opmH*, *mexEF-oprN*, *oprD*, *mexGHI-opmD*, and *mexAB*). As expected, all analogues showed an increase in potency, including dehydroxy-promysalin (−)-**NC**, which possesses a 74 picomolar IC_50_, suggesting that efflux through one or more of these pumps is in fact playing at least some role in promysalin resistance in wild-type *PA*. Of the newly synthesized analogues, acrylamide (−)-**4** showed the most marked increase in activity, with a 1000-fold decrease in IC_50_ value relative to the parent *PA14* strain and displayed single-digit nanomolar inhibitory activity. This may indicate that (−)-**4**, while a very potent inhibitor of *PA* Sdh, is particularly sensitive to efflux.

Unfortunately, none of our newly synthesized analogues had improved MIC values against any tested *PA* strain relative to that of promysalin (MIC values >250 μM for all analogues reported herein), indicating that we were unsuccessful in fully overcoming *PA* resistance mechanisms.

## Discussion

We remain interested in the investigation of the mechanisms by which *PA* can generate resistance against promysalin. While we anticipated that efflux and hydrolysis were the most likely mechanisms by which this might occur, the work herein demonstrated that our covalent analogues generated were unable to improve upon the MIC of promysalin. Though our results indicate promysalin efflux is likely occurring (as evidenced by the drastically improved IC_50_ in the efflux knockout), we here show that covalent inhibition is not always a viable tactic to combat this process. Potentially, other mechanisms of resistance may be working in concert with the observed efflux mechanism. For example, in the presence of antibiotic stressors like promysalin, *PA* may respond by upregulation of other metabolic pathways. Transcriptomic studies indicate that in *P. putida*, the Entner–Doudoroff pathway is downregulated, and both nutrient uptake and the β-ketoadipate pathway are upregulated in the presence of exogenous promysalin.^23^ This shift in metabolic flux may account for the lack of promysalin susceptibility in *P. putida*, and similar changes in gene expression could also be present in *PA*.

Alternatively, *PA* may be utilizing the phenomenon of persistence to overcome the activity of promysalin.^29^ Bacterial persister cells have been studied for their role in chronic infections in several common bacterial pathogens, including *Staphylococcus aureus*, *E. coli*, and *PA*.^30−32^ Persistent cells are those that are metabolically slow or dormant and are thought to exist in low numbers in all bacterial populations. This allows the population to survive antibiotic stresses, not due to genetic mutation conferring resistance to the antibiotics, but due to decreased metabolic activity. As promysalin’s mechanism of action targets primary metabolism, it stands to reason that *PAO1* and *PA14* persister cells may be able to overcome the inhibitory activity of promysalin, leading to the large observed difference between its MIC and IC_50_. Assays to detect and quantify persister cells in several *PA* strains, including *PAO1* and *PA14*, are ongoing in our lab, and we plan to use the results of these assays to guide future directions for the development of promysalin.

## Supporting Information Available

Detailed synthetic procedures and characterization data for all new compounds; ^1^H and ^13^C NMR spectra; primers used in strain generation; and growth/inhibition curves for all tested strains (PDF)
