# Supplementary material for: Structure-Based Design of Promysalin Analogues to Overcome Mechanisms of Bacterial Resistance
Source: ACS Omega. 2023 Mar 22;8(13):12558–64. doi: 10.1021/acsomega.3c00884 (PMC10077553; doi:10.1021/acsomega.3c00884)
Supplement: Supplementary file 1 — ao3c00884_si_001.pdf [file ao3c00884_si_001.pdf]

Structure-Based Design of Promysalin Analogues to Overcome Mechanisms of Bacterial Resistance

Andrew R. Mahoney[1], Kelly M. Storek[2], and William M. Wuest[1]

[1]Department of Chemistry, Emory University, and Emory Antibiotic Resistance Center, Emory University School of Medicine, Atlanta, GA 30322, USA

[2]Department of Infectious Diseases, Genentech, Inc. South San Francisco, CA 94080, USA

Supplementary Information

Structure-Based Design of Promysalin Analogues to Overcome Mechanisms of Bacterial Resistance

Andrew R. Mahoney[1], Kelly M. Storek[2], and William M. Wuest[1]

[1]Department of Chemistry, Emory University, and Emory Antibiotic Resistance Center, Emory University School of Medicine, Atlanta, GA 30322, USA

[2]Department of Infectious Diseases, Genentech, Inc. South San Francisco, CA 94080, USA

Supplementary Information

Table of Contents

1. Synthesis --S3

Experimental Procedures and Characterization Data ----------------------------------S3

2. Biology-- S21

Table S1: List of Primers--- S21

Figure S1: PA Growth/Inhibition Curves -- S23

Figure S2: Structures of Tested Compounds -- S24

Table S2: IC_{50} Assay Results --- S25

Figure S3: IC_{50} Assay Curves --- S25

3. References -- S26

4. Appendix: Spectral Data --- S27

1. Synthesis

Experimental Procedures and Characterization Data

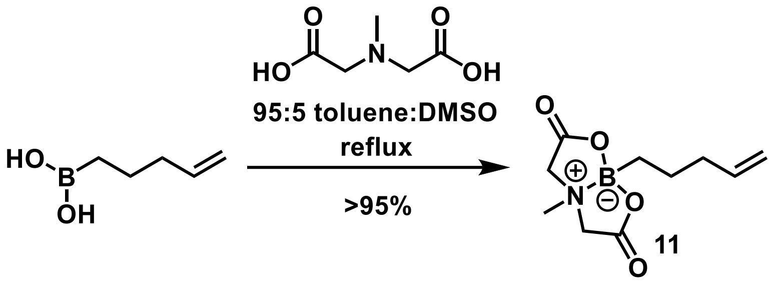

4-methyl-8-(pent-4-en-1-yl)dihydro-4λ^4,8λ^4-[1,3,2]oxazaborolo[2,3-b][1,3,2]oxazaborole-2,6(3H,5H)-dione 11. To a solution of pent-4-en-1-ylboronic acid (0.5 M in THF, 8.340 mL, 4.170 mmol) in 95:5 toluene:dimethylsulfoxide (100 mL) was added N-methyliminodiacetic acid (0.6134 g, 4.170 mmol, 1 eq). The flask was fitted with a toluene-filled Dean Stark condenser, heated to reflux, and stirred overnight. The reaction mixture was then cooled to room temperature and concentrated *in vacuo* to a volume of about 5 mL. Acetone (~5 mL) was added, precipitating a tan solid upon trituration. Ether (~5 mL) was then added to precipitate additional solid. The supernatant was removed to a separate flask and this process was repeated three times. The resulting solids were combined and rinsed one more time with acetone and ether, which was then again removed to give the title compound as an off-white powder (940 mg, >95% yield). **^{1}H NMR** (500 MHz, $CDCl_3$): δ 5.78 (ddt, J = 17.0, 10.22, 6.69 Hz, 1H), 4.96 (dd, J = 17.15, 10.15 Hz, 2H), 3.98 (d, J = 16.60 Hz, 2H), 3.69 (d, J = 16.62 Hz, 2H), 2.90 (s, 3H), 2.09 (dt, J = 7.15, 7.14, 2H), 1.45 (dtd, J = 15.11, 9.06, 7.44, 3.89 Hz, 2H), 0.69 – 0.49 (m, 2H). **^{13}C NMR** (125 MHz, $CDCl_3$): δ 167.77, 138.80, 114.68, 61.92, 45.83, 40.97, 36.61, 23.39. **HRMS** Accurate Mass (ES^+): Found 223.11365 (+0.24 ppm), $C_{10}H_{15}O_4N^{10}B$ (M - H^+) requires 223.11359.

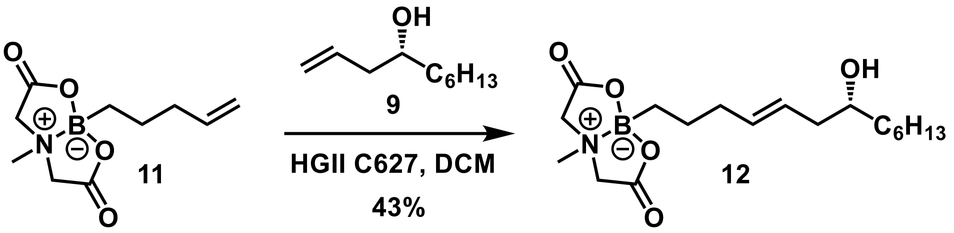

(R)-8-(7-hydroxytridec-4-en-1-yl)-4-methyldihydro-4λ^4,8λ^4-[1,3,2]oxazaborolo[2,3-b][1,3,2]oxazaborole-2,6(3H,5H)-dione 12. Hoveyda-Grubbs C627 catalyst (55.7 mg, 0.089 mmol, 10 mol %) was added to a solution of MIDA boronate **11** (200 mg, 0.889 mmol, 1 eq) and **9**[1] (416.6 mg, 2.666 mmol, 3 eq) dissolved in dichloromethane (6 mL). The solution stirred at room temperature overnight, then was concentrated under reduced pressure. Product was purified by silica column chromatography (100% Et_2O, then 20-30% acetone in dichloromethane). to give the title compound as a dark brown oil (135.6 mg, 43% yield). **^{1}H NMR** (600 MHz, $CDCl_3$): δ 5.57 – 5.47 (m, 1H), 5.45-5.33 (m, 1H), 3.97 (d, J = 16.47 Hz, 2H), 3.70 (d, J = 16.61 Hz, 2H), 3.56 (br s, 1H), 2.90 (s, 3H), 2.27 – 2.15 (m, 1H), 2.13 – 1.95 (m, 3H), 1.88 – 1.56 (m, 2H), 1.46 – 1.38 (m, 4H), 1.35 – 1.16 (m, 8H), 0.91 – 0.67 (t, J = 6.00 Hz, 3H), 0.65 – 0.44 (m, 2H). **^{13}C NMR** (125

MHz, $CDCl_3$): δ 167.75, 134.05, 126.54, 70.97, 61.91, 45.83, 40.70, 36.88, 35.45, 31.82, 29.35, 25.75, 25.68, 23.87, 22.59, 14.06. **$[\alpha]^{25}_D$** +4.8 (c 1.41, DMSO). **HRMS** Accurate Mass (ES^+): Found 354.24393 (-1.99 ppm), $C_{18}H_{33}O_5N^{11}B$ ($M + H^+$) requires 354.24463.

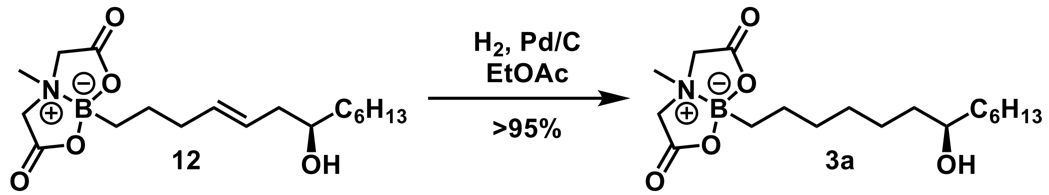

(R)-8-(7-hydroxytridecyl)-4-methyldihydro-4λ^4,8λ^4-[1,3,2]oxazaborolo[2,3-b][1,3,2]oxazaborole-2,6(3H,5H)-dione 3a. To a solution of **12** (115 mg, 0.326 mmol, 1 eq) dissolved in ethyl acetate (5 mL) was added palladium on carbon (10 wt. %, 32.6 mg). The flask was purged five times with hydrogen and was stirred overnight at room temperature under a hydrogen atmosphere. The following day, the solution was filtered through a pad of Celite and was concentrated under vacuum, yielding the title compound as a white powder (134.9 mg, >95% yield). **^{1}H NMR** (400 MHz, $CDCl_3$): δ 3.82 (d, J = 16.28 Hz, 2H), 3.67 (d, J = 16.28 Hz, 2H), 3.58 (br s, 1H), 2.90 (s, 3H), 1.58 (s, 2H) 1.49-1.20 (m, 18H), 0.88 (t, J = 8.00 Hz, 3H), 0.61 (t, J = 7.52 Hz, 2H). **^{13}C NMR** (125 MHz, $CDCl_3$): δ 167.77, 71.91, 61.94, 45.79, 37.55, 37.49, 32.73, 31.84, 29.46, 29.38, 25.65, 25.53, 23.91, 22.60, 14.06. **$[\alpha]^{25}_D$** +0.6 (c 1.05, DMSO). **HRMS** Accurate Mass (ES^+): Found 390.22262 (+0.56 ppm), $C_{18}H_{34}O_5N^{11}B^{35}Cl$ ($M + Cl^-$) requires 390.22240.

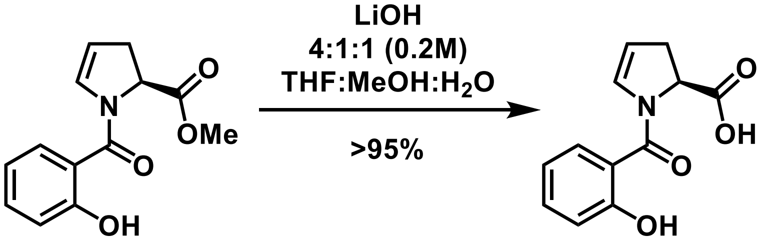

17

(S)-1-(2-hydroxybenzoyl)-2,3-dihydro-1H-pyrrole-2-carboxylic acid 17. To a solution of methyl (S)-1-(2-hydroxybenzoyl)-2,3-dihydro-1H-pyrrole-2-carboxylate[2] (31.2 mg, 0.126 mmol, 1 eq.) dissolved in 4:1 THF:MeOH (2.5 mL) was added a solution of LiOH (52.9 mg, 1.26 mmol, 10 eq.) dissolved in water (0.5 mL) dropwise. The reaction was stirred for 5 hours at room temperature, then was carefully acidified to pH=5 with 1M HCl. The organic layer was extracted 5x with dichloromethane and washed once with brine, then was dried over anhydrous Na_2SO_4, filtered, and concentrated to afford the title compound as a pale yellow oil that was used in the next step without further purification (30.4 mg, >95% yield).

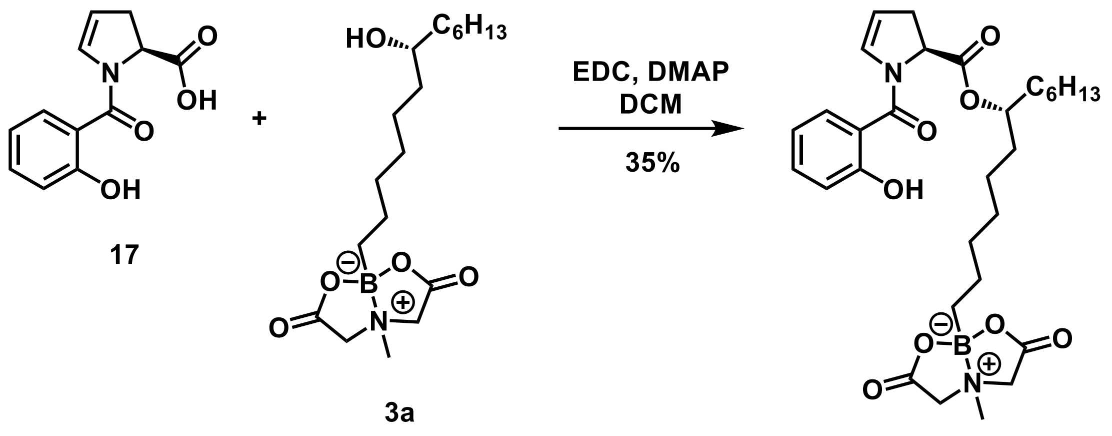

17 3a 18

(R)-1-(4-methyl-2,6-dioxotetrahydro-2H-4λ^4,8λ^4-[1,3,2]oxazaborolo[2,3-b][1,3,2]oxazaborol-8-yl)tridecan-7-yl (S)-1-(2-hydroxybenzoyl)-2,3-dihydro-1H-pyrrole-2-carboxylate 18. A solution of acid **17** (47.1 mg, 0.202 mmol, 1.5 eq) and alcohol **3a** (49.9 mg, 0.140 mmol, 1 eq) dissolved in dichloromethane (4 mL) was cooled to 0 °C, and EDC (65.2 mg, 0.420 mmol, 3 eq.) and DMAP (68.4 mg, 0.420 mmol, 4 eq.) were added. The reaction mixture was warmed to room temperature and stirred overnight, then was added to water. The organic layer was extracted 5x with dichloromethane, washed once with brine, and dried over anhydrous Na_2SO_4, then was filtered and concentrated *in vacuo*. Product was then purified by silica column chromatography (0-50% acetone in dichloromethane, then 100% iPrOH) to afford the title compound as a white solid (28.3 mg, 35% yield). **^{1}H NMR** (600 MHz, $CDCl_3$): δ 9.73 (s, 1H), 7.41 (dd, J = 7.82, 1.66, 1H), 7.36 (ddd, J = 8.64, 7.32, 1.66, 1H), 6.97 (dd, J = 8.31, 1.13), 6.89 (m, 1H), 6.76 (br s, 1H), 5.34-5.22 (m, 1H), 5.03-4.85 (m, 2H), 3.93-3.84 (m, 2H), 3.71-3.65 (m, 2H), 3.18-3.06 (m, 1H), 2.90-2.83 (m, 3H), 2.74-2.64 (m, 1H), 0.85 (t, J = 7.21 Hz, 3H), 0.60-0.55 (m, 2H). **^{13}C NMR** (125 MHz, $CDCl_3$): δ 170.81, 167.54, 167.44, 158.98, 158.67, 133.35, 130.75, 128.26, 119.00, 117.83, 110.73, 76.00, 71.96, 61.90, 45.66, 37.53, 34.11, 32.46, 31.65, 29.67, 29.10, 28.91, 25.53, 25.22, 24.84, 23.59, 22.52, 14.03. **$[\alpha]^{25}_D$** -25.1 (c 0.342, DMSO). **HRMS** Accurate Mass (ES^+): Found 570.3112 (+0.76 ppm), $C_{30}H_{44}O_8N_2{}^{11}B$ ($M + H^+$) requires 571.31852.

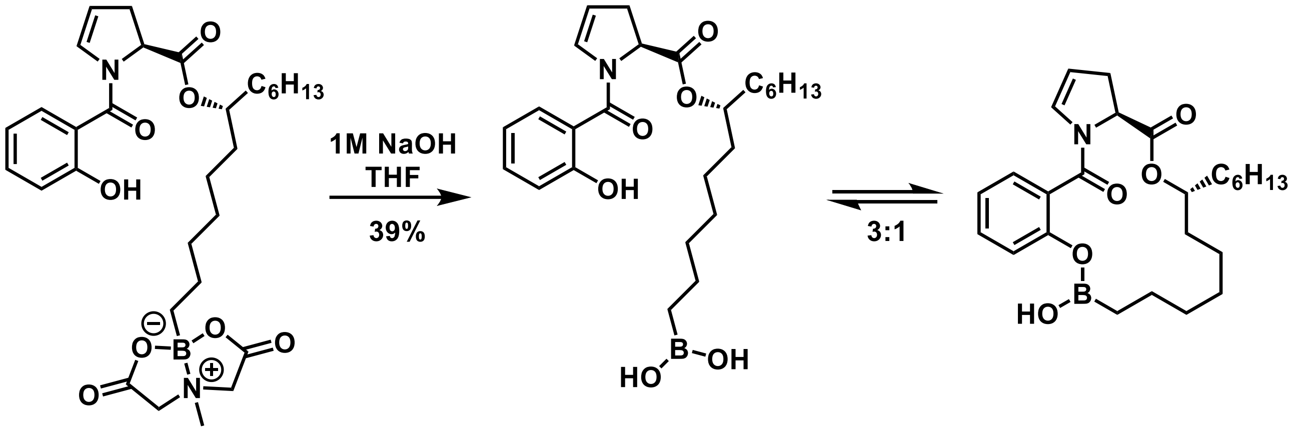

18 3 3-cyc

((R)-7-(((S)-1-(2-hydroxybenzoyl)-2,3-dihydro-1H-pyrrole-2-carbonyl)oxy)tridecyl)boronic acid 3, 3-cyc. To a solution of MIDA boronate **18** (15.4 mg, 0.0270 mmol) dissolved in tetrahydrofuran (1 mL) was added 1 M aqueous sodium hydroxide (0.3 mL) and the reaction stirred at room temperature for ten minutes. The reaction was quenched with phosphate buffer (1M, pH=7, 1 mL) and Et_2O (1 mL) was added. The organic layer was extracted 3x with 1:1 Et_2O:THF, dried over anhydrous $MgSO_4$, filtered, and concentrated under reduced pressure. Product was purified by HPLC to afford the title compounds as a white solid (4.8 mg, 39% yield). **^{1}H NMR** (600 MHz, DMSO-d_6, 25°C): δ 10.06 (s, 0.30 H), 9.91 (s, 1H), 7.33 (s, 0.30 H), 7.29 (s, 1H), 7.25 (td, J = 7.8, 7.4, 1.8 Hz, 1H), 7.12 – 7.06 (m, 1H), 7.05-6.99 (m, 0.30 H), 6.99-6.93 (m, 0.30 H), 6.87 (d, J = 8.2 Hz, 1H), 6.83 (td, J = 7.5, 2.8 Hz, 1H), 6.19-6.10 (m, 1H), 5.30-5.20 (m, 0.30 H), 5.11-5.03 (m, 1H), 4.77 (dd, J = 11.5, 5.2 Hz, 3H), 4.50-4.42 (m, 0.30 H), 4.32 (t, J = 5.1 Hz, 1H), 3.79 – 3.59 (m, 1H), 3.39 (qd, J = 6.9, 4.7 Hz, 1H), 3.12 – 3.01 (m, 1H), 2.00-1.88 (m, 0.30 H), 1.45 (s, 7H), 1.37 – 0.91 (m, 36H), 0.88 – 0.67 (m, 7H), 0.52 (dt, J = 15.5, 7.8 Hz, 2H), 0.45-0.30 (m, 0.30 H). **^{13}C NMR** (125 MHz, DMSO-d_6, 25°C): δ 171.44, 157.24, 134.39, 133.38, 130.84, 128.30, 122.97, 119.12, 117.94, 116.64, 88.67, 75.79, 58.34, 53.42, 34.58, 34.33, 31.74, 30.73, 29.71, 29.11, 28.13, 26.46, 25.41, 23.08, 22.55, 14.07. **$[\alpha]^{25}_D$** -7.2 (c 0.68, $CHCl_3$). **HRMS** Accurate Mass (ES^+): Found 458.26973 (-2.42 ppm), $C_{25}H_{37}O_6N^{11}B$ ($M - H^+$) requires 458.27084. Found 442.27477 (-2.62 ppm) $C_{25}H_{37}O_5N^{11}B$ ($M + H^+$) requires 442.27593.

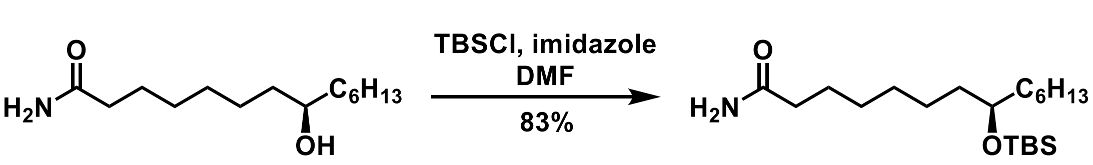

10 19

(R)-8-((tert-butyldimethylsilyl)oxy)tetradecanamide 19. To a solution of (R)-8-hydroxytetradecanamide **10**[2] (56.3mg, 0.231 mmol, 1 eq) dissolved in dimethylformamide (3 mL) was added tert-butyldimethylsilyl chloride (87.2 mg, 0.578 mmol, 2.5 eq) and imidazole (78.7 mg, 1.157 mmol, 5 eq). The reaction was stirred at room temperature overnight then was added to water. The organic layer was extracted 3x with 1:1 EtOAc: hexanes, washed once with brine, and

dried over anhydrous Na_2SO_4, then was filtered and concentrated *in vacuo*. Product was purified by silica column chromatography (25-100% EtOAc in hexanes) to afford the title compound as an off-white solid (68.8 mg, 83% yield). **^{1}H NMR** (600 MHz, $CDCl_3$): δ 5.79 (br s, 1H), 5.45 (br s, 1H), 3.63-3.52 (m, 1H), 2.23-2.12 (m, 2H), 1.66-1.54 (m, 2H), 1.41-1.15 (m, 19H), 0.84 (m, 12H), 0.12--0.14 (m, 6H). **^{13}C NMR** (125 MHz, $CDCl_3$): δ 175.8, 72.31, 37.14, 37.05, 35.94, 31.87, 29.59, 29.52, 29.26, 25.92, 25.51, 25.27, 25.16, 22.61, 18.14, 14.07, 4.42. **$[\alpha]^{25}_D$** -1.4 (c 1.03, $CHCl_3$). **HRMS** Accurate Mass (ES^+): Found 356.29909 (+0.16 ppm), $C_{20}H_{42}O_2N^{28}Si$ ($M - H^+$) requires 356.29903.

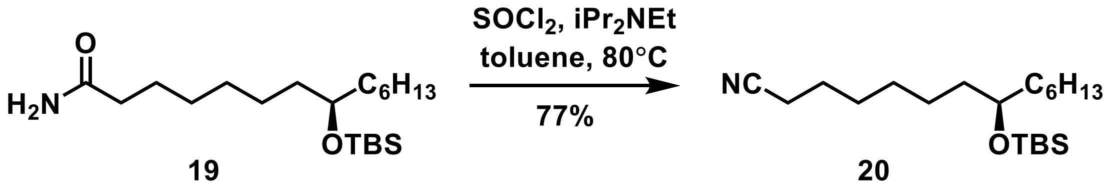

19 20

(R)-8-((tert-butyldimethylsilyl)oxy)tetradecanenitrile 20. To a solution of amide **19** (66.6 mg, 0.186 mmol, 1 eq.) dissolved in toluene (3 mL) was added iPr_2NEt (0.194 mL, 1.118 mmol, 6 eq.) and thionyl chloride (0.04 mL, 0.558 mmol, 3 eq.). The solution was heated to 80°C, resulting in a change in color to a deep red, and was stirred at that temperature overnight. The reaction mixture was then cooled to room temperature and added to water. The organic layer was extracted 4x with hexanes, washed once with brine, and dried over anhydrous Na_2SO_4, then filtered and concentrated *in vacuo*. Product was purified by silica column chromatography (0-20% EtOAc in hexanes) to yield the title compound as a red oil (48.3 mg, 77% yield). **^{1}H NMR** (500 MHz, $CDCl_3$): δ 3.61 (p, J = 5.7 Hz, 1H), 2.33 (t, J = 7.1 Hz, 2H), 1.65 (dt, J = 15.0, 7.2 Hz, 2H), 1.50 – 1.19 (m, 20H), 0.95 – 0.80 (m, 12H), 0.03 (d, J = 2.0 Hz, 6H). **^{13}C NMR** (125 MHz, $CDCl_3$): δ 119.76, 77.27, 77.02, 76.77, 72.20, 37.15, 36.89, 31.87, 29.52, 29.02, 28.67, 25.91, 25.34, 25.26, 24.95, 22.61, 18.12, 17.09, 14.07, -4.41. **$[\alpha]^{25}_D$** -2.4 (c 0.25, $CHCl_3$). **HRMS** Accurate Mass (ES^+): Found 340.30165 (-4.02 ppm), $C_{20}H_{42}ON^{28}Si$ ($M + H^+$) requires 340.30302.

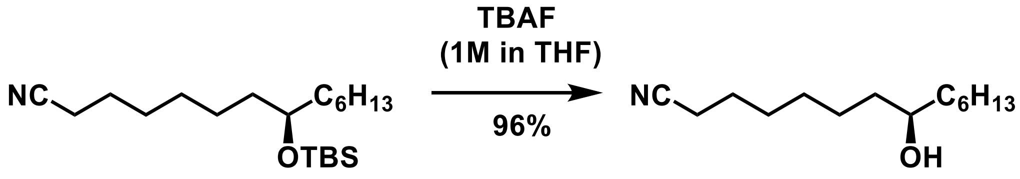

20 2a

(R)-8-hydroxytetradecanenitrile 2a. To a flask containing silyl ether **20** (46.3 mg, 0.136 mmol, 1 eq) was added tetrabutylammonium fluoride (1.0 M in THF, 1.36 mL, 1.36 mmol, 10 eq.). The reaction stirred at room temperature for two hours, then was quenched with saturated aqueous ammonium chloride (4 mL). The organic layer was extracted five times with diethyl ether. The combined organic layers were washed five times with saturated aqueous ammonium chloride and five times with brine, then dried over anhydrous Na_2SO_4, filtered, and concentrated under vacuum

to give the title compound as a red oil. (29.5 mg, 96% yield). **^{1}H NMR** (600 MHz, $CDCl_3$): δ 3.64-3.49 (m, 1H), 2.33 (t, J = 7.13 Hz, 2 H), 1.66 (q, J = 7.22 Hz, 2H), 1.50-1.22 (m, 20H), 0.88 (t, J = 7.07 Hz, 3H). **^{13}C NMR** (125 MHz, $CDCl_3$): δ 119.76, 71.82, 37.56, 37.23, 31.81, 29.67, 29.33, 28.80, 28.61, 25.59, 25.32, 25.28, 22.59, 17.09, 14.05. **$[\alpha]^{25}_D$** -2.6 (c 0.96, $CHCl_3$). **HRMS** Accurate Mass (ES^+): Found 226.21618 (-1.60 ppm), $C_{14}H_{28}ON$ ($M + H^+$) requires 226.21654.

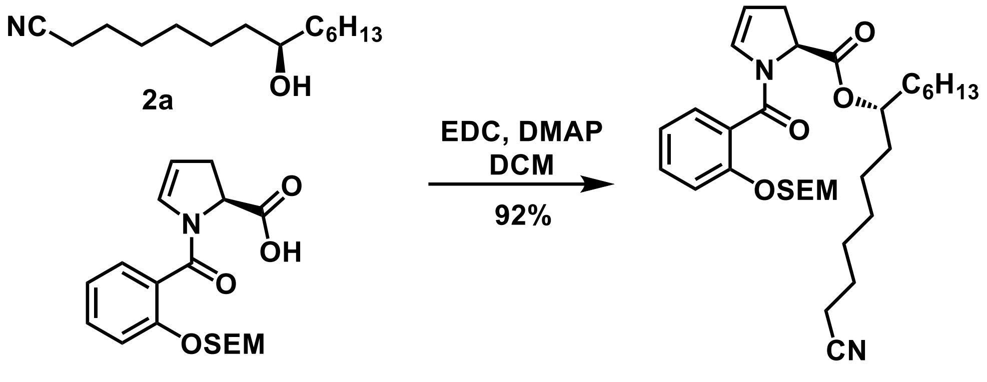

(R)-1-cyanotridecan-7-yl (S)-1-(2-((2-(trimethylsilyl)ethoxy)methoxy)benzoyl)-2,3-dihydro-1H-pyrrole-2-carboxylate 22. A solution of methyl (S)-1-(2-((2-(trimethylsilyl)ethoxy)methoxy)benzoyl)-2,3-dihydro-1H-pyrrole-2-carboxylate **21**[1] (67.7 mg, 0.156 mmol, 2 eq.) and alcohol **2a** (21.0 mg, 0.0932 mmol, 1 eq.) dissolved in dichloromethane (3 mL) was cooled to 0°C and EDC (35.7 mg, 0.186 mmol, 2 eq.) and DMAP (45.5 mg, 0.373 mmol, 4 eq.) were added. The reaction mixture was warmed to room temperature and stirred overnight, then was added to water. The organic layer was extracted 5x with DCM, washed once with brine, and dried over anhydrous Na_2SO_4, then was filtered and concentrated *in vacuo*. Product was purified by silica column chromatography (0-20% EtOAc in dichloromethane, then 0-10% methanol in dichloromethane) to afford the title compound as a vivid yellow oil (48.9 mg, 92% yield). **^{1}H NMR** (500 MHz, $CDCl_3$): δ δ 7.34 (d, J = 7.3 Hz, 2H), 7.19 (d, J = 8.0 Hz, 1H), 7.04 (td, J = 7.5, 1.0 Hz, 1H), 6.16 (dt, J = 4.3, 2.2 Hz, 1H), 5.28 – 5.18 (m, 3H), 5.02 (dt, J = 4.3, 2.5 Hz, 1H), 4.96 (dd, J = 11.5, 5.0 Hz, 2H), 3.74 (t, J = 8.0 Hz), 3.12 (ddt, J = 16.8, 11.7, 2.4 Hz, 1H), 2.66 (ddt, J = 17.0, 4.8, 2.7 Hz, 1H), 2.30 (t, J = 7.1 Hz, 2H), 1.72 – 1.49 (m, 7H), 1.49 – 1.16 (m, 18H), 0.93 (dd, J = 9.3, 7.2 Hz, 3H), 0.86 (t, J = 8.6 Hz, 3H), -0.02 (s, 9H). **^{13}C NMR** (125 MHz, $CDCl_3$): δ 170.70, 164.86, 153.72, 131.12, 130.96, 128.86, 125.86, 121.87, 119.84, 115.18, 108.13, 93.26, 77.28, 77.02, 76.77, 75.10, 66.49, 58.02, 34.24, 34.16, 33.96, 31.71, 29.67, 29.15, 28.44, 25.24, 25.20, 24.71, 22.56, 18.04, 17.01, 14.03, -1.42. **$[\alpha]^{25}_D$** -46.6 (c 0.86, $CHCl_3$). **HRMS** Accurate Mass (ES^+): Found 571.35500 (-2.07 ppm), $C_{32}H_{51}O_5N_2{}^{28}Si$ ($M + H^+$) requires 571.35618.

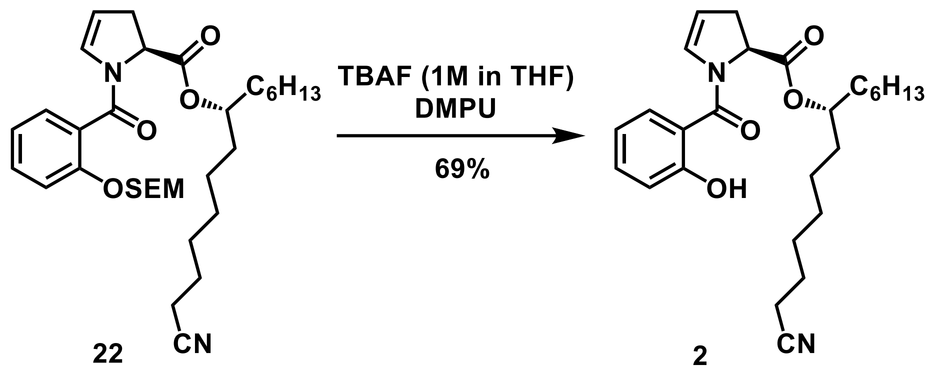

(R)-1-cyanotridecan-7-yl (S)-1-(2-hydroxybenzoyl)-2,3-dihydro-1H-pyrrole-2-carboxylate 2. To a solution of silyl ether **22** (23.5 mg, 0.0412 mmol, 1 eq.) dissolved in DMPU (0.823 mL) was added TBAF (1M in THF, 0.823 mL, 0.823 mmol, 20 eq.) dropwise. The reaction was stirred at room temperature for 2 hours, then was quenched with saturated aqueous NH_4Cl (3 mL) and acidified to pH=5. The organic layer was extracted 5x with Et_2O, washed 5x with saturated aqueous NH_4Cl and 5x with brine, and dried over Na_2SO_4, then filtered and concentrated *in vacuo*. Product was purified by silica column chromatography, followed by HPLC to yield the title compound as a clear oil. (12.6 mg, 69% yield). **^{1}H NMR** (500 MHz, $CDCl_3$): δ 9.86 (s, 1H), 7.42 (dd, J = 7.81, 1.68 Hz, 1H), 7.38 (ddt, J = 8.31 7.32, 2.50, 2.50 Hz, 1H), 7.00 (dd, J = 8.33, 1.09 Hz, 1H), 6.89 (td, J = 7.59, 1.18 Hz, 1H), 6.82 (br s, 1H), 5.27 (dt, J = 4.36, 2.69 Hz, 1H), 5.03-4.89 (m, 2H), 3.12 (ddt, J = 16.50, 11.33, 2.31, 2.31 Hz, 1H), 2.70 (ddd, J = 16.57, 5.31, 2.60 Hz, 1H), 2.32 (t, J = 7.13 Hz, 2H), 1.70-1.48 (m, 7H), 1.48-1.15 (m, 15H), 0.85 (t, J = 7.18 Hz, 3H). **^{13}C NMR** (125 MHz, $CDCl_3$): δ 170.77, 170.66, 159.21, 133.44, 130.91, 128.28, 119.82, 118.81, 117.97, 116.80, 110.50, 75.65, 59.49, 34.09, 33.95, 33.81, 31.66, 29.09, 28.49, 28.42, 25.22, 25.17, 24.72, 22.52, 17.04, 14.03. **$[\alpha]^{25}_D$** -56.0 (c 0.59, $CHCl_3$). **HRMS** Accurate Mass (ES^+): Found 441.27391 (-1.97 ppm), $C_{26}H_{37}O_4N_2$ (M + H^+) requires 441.27478.

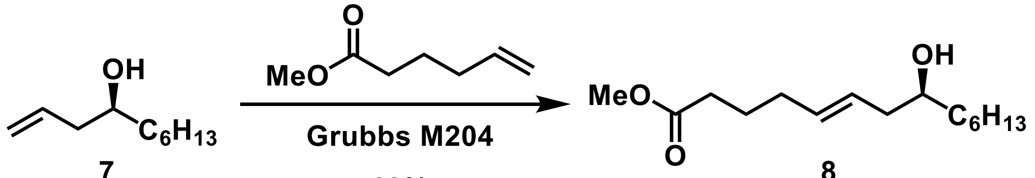

Methyl (S,E)-8-hydroxytetradec-5-enoate 8. A 10 mL round-bottom flask was flame-dried and purged thrice with argon. **7**[1] (306.9 mg, 1.964 mmol, 1.0 eq.), methyl hex-5-enoate (1.12 mL, 7.856 mmol, 4.0 eq.), and Grubbs II catalyst (M204, 166.9 mg, 0.196 mmol, 0.1 eq.) were sequentially added. The dark red reaction stirred at room temperature overnight. Crude product was purified by silica column chromatography (50-100% DCM/hexanes to remove ester dimer, then 0-10% EtOAc/DCM to elute cross product), yielding the title compound as a brown oil (348.5 mg, 69% yield). **^{1}H NMR** (500 MHz, $CDCl_3$): δ 5.56 – 5.38 (m, 2H), 3.66 (s, 3H), 3.61 – 3.54 (m, 1H), 2.31 (t, J = 7.5 Hz, 2H), 2.22 (dq, J = 14.4, 4.4 Hz, 1H), 2.10 – 2.03 (m, 3H), 1.75 – 1.67 (m, 2H), 1.61 (s, 2H), 1.43 (t, J = 5.7 Hz, 3H), 1.33 – 1.22 (m, 8H), 0.89 – 0.85 (m, 3H). **^{13}C NMR**

(125 MHz, $CDCl_3$): δ 174.25, 133.12, 127.42, 71.11, 51.66, 40.84, 36.92, 33.51, 32.11, 31.97, 29.48, 25.80, 24.64, 22.76, 14.23. **$[\alpha]^{25}_D$** +0.33 (c 0.60, $CHCl_3$). **HRMS** Accurate Mass (ES^+): Found 257.21101 (-0.43 ppm), $C_{15}H_{29}O_3$ ($M + H^+$) requires 257.21112.

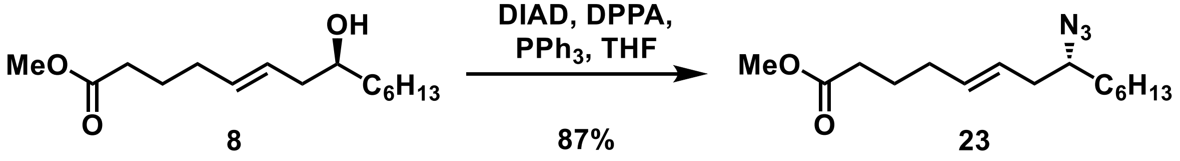

Methyl (R,E)-8-azidotetradec-5-enoate 23. A 10 mL round-bottom flask was flame-dried and purged thrice with argon. Alcohol **8** (250 mg, 0.975 mmol, 1.0 eq.) and triphenylphosphine (1.023 g, 3.900 mmol, 4.0 eq.) were added and dissolved in THF (12 mL). The solution was cooled to 0°C and diisopropyl azodicarboxylate (0.758 mL, 3.900 mmol, 4.0 eq.) and diphenylphosphoryl azide (0.839 mL, 3.900 mmol, 4.0 eq.) were added dropwise. The cloudy white solution was warmed to RT and stirred two hours, then was heated to 45°C and stirred two additional hours, steadily turning more yellow and less cloudy. Crude product was concentrated *in vacuo* and purified by silica column chromatography (10% EtOAc/hexanes, coeluting with DIAD and PPh_3). Product was further purified by preparative TLC (10% EtOAc/hexanes) and triturated with cold pentane. Solution was decanted and concentrated *in vacuo*, affording the title compound as a clear oil (239.2 mg, 87% yield). **^{1}H NMR** (600 MHz, $CDCl_3$): δ 5.50 (dq, J = 11.6, 5.9, 5.2 Hz, 1H), 5.43 (dt, J = 15.0, 6.9 Hz, 1H), 3.66 (s, 3H), 3.26 (dt, J = 13.0, 5.8 Hz, 1H), 2.31 (t, J = 7.5 Hz, 2H), 2.24 (dp, J = 14.1, 7.0 Hz, 2H), 2.09 – 2.03 (m, 2H), 1.71 (p, J = 7.5 Hz, 2H), 1.52 – 1.39 (m, 3H), 1.30 (dd, J = 13.9, 8.4 Hz, 7H), 0.88 (t, J = 6.9 Hz, 3H). **^{13}C NMR** (151 MHz, $CDCl_3$): δ 174.21, 132.99, 126.58, 62.87, 51.62, 37.81, 34.02, 33.48, 32.05, 31.84, 29.20, 26.17, 24.58, 22.71, 14.18. **$[\alpha]^{25}_D$** +10.4 (c 0.45, $CHCl_3$). **HRMS** Accurate Mass (ES^+): Found 304.19946 (-0.28 ppm), $C_{15}H_{27}O_2N_3{}^{23}Na$ ($M + Na^+$) requires 304.19955.

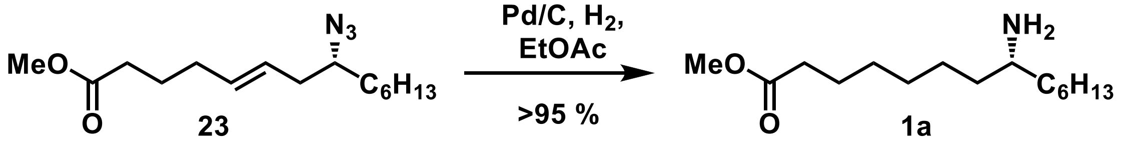

Methyl (R)-8-aminotetradecanoate 1a. A 20 mL scintillation vial containing azide **23** (77.2 mg, 0.274 mmol, 1.0 eq.) was purged thrice with argon. Palladium on activated carbon (10% Pd, 29.2 mg, 0.274 mmol, 1.0 eq.) was added, followed by ethyl acetate (3 mL). The vial was purged five times with hydrogen and the reaction was left to stir under hydrogen atmosphere (1 atm) for 16 hours. The dark grey suspension was filtered over a pad of celite, eluting with 100% EtOAc, and concentrated *in vacuo*. Product was purified by silica column chromatography (0-10% MeOH/DCM) to yield the title compound as a pale yellow oil (70.1 mg, >95% yield). **^{1}H NMR** (500 MHz, $CDCl_3$): δ 3.69-3.57 (m, 5H), 2.74 (br s, 1H), 2.25 (t, J = 7.5 Hz, 2H), 1.61 – 1.53 (m, 2H), 1.45 – 1.38 (m, 2H), 1.37 – 1.18 (m, 17H), 0.83 (t, J = 6.3 Hz, 3H). **^{13}C NMR** (126 MHz, $CDCl_3$) δ 174.28, 51.49, 51.47, 36.53, 36.42, 34.07, 31.83, 29.40, 29.34, 29.09, 25.91, 25.76,

24.91, 22.66, 14.11. **[α]$^{25}_{D}$** -0.43 (c 0.375, $CHCl_3$). **HRMS** Accurate Mass (ES^+): Found 258.24301 (+ 1.00 ppm), $C_{15}H_{32}O_2N$ (M + H^+) requires 258.24276.

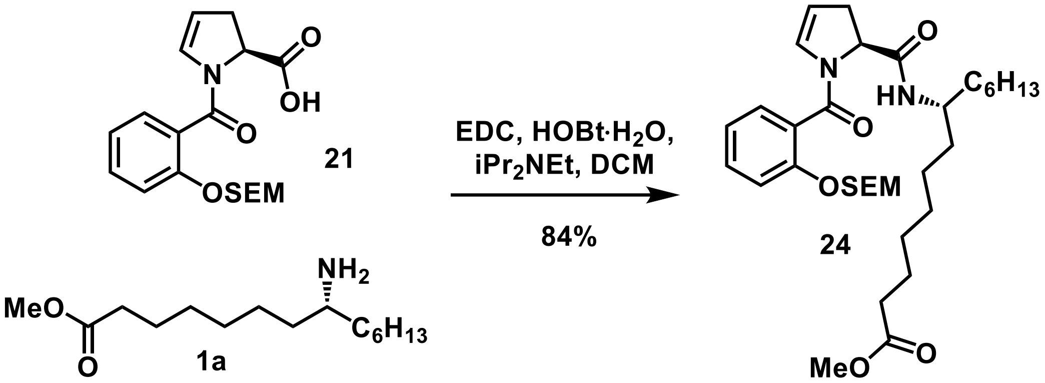

Methyl (R)-8-((S)-1-(2-hydroxybenzoyl)-2,3-dihydro-1H-pyrrole-2-carboxamido)tetradecanoate 24. A 20 mL scintillation vial containing acid **21** (48.0 mg, 0.132 mmol, 1.5 eq.) and 3Å molecular sieves was purged 3x with argon. DCM (2.5 mL) was added and the flask was cooled to 0°C. EDC (26.0 mg, 0.132 mmol, 1.5 eq.), HOBt•H_2O (16.0 mg, 0.091 mmol, 1.0 eq.), and diisopropylethylamine (0.040 mL, 0.23 mmol, 2.5 eq.) were added sequentially. Amine **1a** (22.0 mg, 0.091 mmol, 1.0 eq.) was transferred into the vial dropwise in DCM (2.5 mL). The reaction stirred overnight, allowing it to warm to room temperature. Water was added and the aqueous layer was extracted 5x with DCM, washed once with brine, and back-extracted twice. Combined organic layers were dried over anhydrous Na_2SO_4, filtered, and concentrated *in vacuo.* Crude product was purified by silica column chromatography (15-35% EtOAc/hex) to yield a yellow oil (43.5 mg, 84% yield). **^{1}H NMR** (500 MHz, $CDCl_3$): δ 7.37 (td, J = 8.7, 1.6 Hz, 1H), 7.28 (br s, 1H), 7.23 (d, J = 8.4 Hz, 1H), 7.05 (t, J = 7.4 Hz, 1H), 6.04 (s, 1H), 5.28 – 5.18 (m, 2H), 5.17 – 5.11 (m, 1H), 5.08 – 5.03 (m, 1H), 3.90 (br s, 1H), 3.71 (td, J = 8.6, 3.5 Hz, 2H), 3.63 (s, 3H), 3.03 (d, J = 74.6 Hz, 2H), 2.25 (t, J = 7.5 Hz, 2H), 1.56 (p, J = 7.5 Hz, 2H), 1.51 – 1.43 (m, 2H), 1.37 – 1.18 (m, 17H), 0.96 – 0.90 (m, 2H), 0.82 (t, J = 6.6 Hz, 3H), -0.03 (s, 9H). **^{13}C NMR** (151 MHz, $CDCl_3$): δ 174.26, 169.78, 166.04, 153.55, 131.54, 129.55, 128.54, 125.51, 122.20, 114.79, 111.83, 93.24, 66.92, 59.46, 51.52, 49.37, 35.53, 35.35, 34.13, 31.83, 29.79, 29.32, 29.30, 29.21, 25.84, 25.68, 24.97, 22.70, 18.19, 14.17, -1.30. **[α]$^{25}_{D}$** -52.132 (c 0.333, $CHCl_3$). **HRMS** Accurate Mass (ES^+): Found 603.38282 (+ 0.71 ppm), $C_{33}H_{55}O_6N_2{}^{28}Si$ (M + H^+) requires 603.38239.

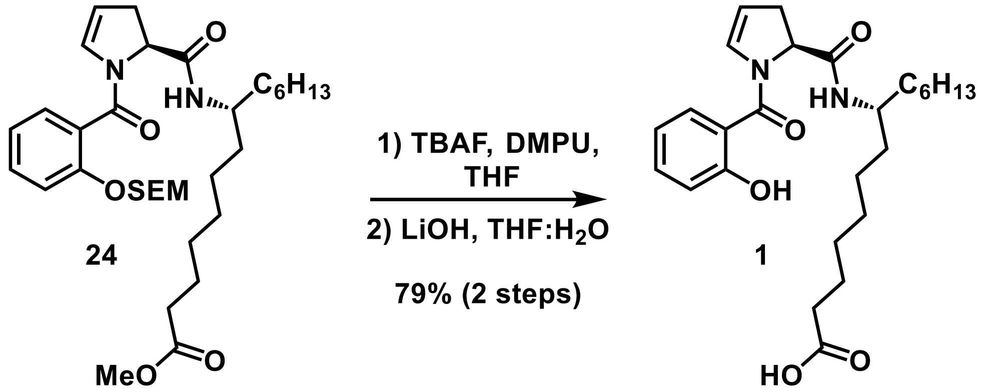

(R)-8-((S)-1-(2-hydroxybenzoyl)-2,3-dihydro-1H-pyrrole-2-carboxamido)tetradecanoic acid 1. A 20 mL scintillation vial containing SEM-protected methyl ester **24** (38.3 mg, 0.064 mmol, 1.0 eq.) was purged thrice with argon. DMPU (1.27 mL) was added, followed by TBAF (1M in THF 1.27 mL, 1.27 mmol, 20 eq.), turning the solution a golden color. The reaction stirred at room temperature for 3 hours, then was quenched with saturated aqueous NH_4Cl (3 mL) and acidified to pH=5. The organic layer was extracted 5x with Et_2O, washed 5x with saturated aqueous NH_4Cl and 5x with brine, and dried over Na_2SO_4, then filtered and concentrated *in vacuo*. Column chromatography (0-75% EtOAc/hexanes) yielded deprotected methyl ester intermediate. **^{1}H NMR** (600 MHz, $CDCl_3$): δ 9.67 (s, 1H), 7.40 – 7.34 (m, 2H), 7.00 (d, J = 8.3 Hz, 1H), 6.89 (t, J = 7.6 Hz, 1H), 6.66 (s, 1H), 6.40 (s, 1H), 5.34 (s, 1H), 5.04 (dd, J = 10.4, 3.9 Hz, 1H), 3.89 (qt, J = 8.6, 5.2 Hz, 1H), 3.66 (s, 3H), 3.10 (d, J = 16.1 Hz, 1H), 2.95 (d, J = 11.8 Hz, 1H), 2.23 (t, J = 7.5 Hz, 2H), 1.56 – 1.50 (m, 2H), 1.49 – 1.43 (m, 2H), 1.40 – 1.17 (m, 20H), 0.86 (t, J = 6.9 Hz, 3H). To this intermediate, THF (4 mL) was added, followed by a solution of LiOH (5.1 mg, 0.212 mmol, 10.0 eq.) in H_2O (0.75 mL). The solution was stirred at room temperature for 4 hours, then was carefully acidified to pH = 4 using HCl (1M) and the aqueous layer was extracted 5x with EtOAc. Combined organic layers were washed twice with brine, back-extracted twice, dried over anhydrous Na_2SO_4, filtered, and concentrated *in vacuo.* Crude product was purified by HPLC to afford the title compound as an off-white solid (7.7 mg, 79% yield). **^{1}H NMR** (600 MHz, $CDCl_3$): δ 7.41 – 7.32 (m, 2H), 6.99 (d, J = 8.2 Hz, 1H), 6.89 (t, J = 7.5 Hz, 1H), 6.61 (s, 1H), 6.44 (s, 1H), 5.33 (s, 1H), 5.06 (dd, J = 10.1, 4.3 Hz, 1H), 3.90 (qt, J = 8.2, 5.3 Hz, 1H), 3.03 – 2.95 (m, 2H), 2.26 (h, J = 8.3 Hz, 2H), 1.57 – 1.50 (m, 2H), 1.49 – 1.42 (m, 2H), 1.42 – 1.35 (m, 2H), 1.35 – 1.16 (m, 20H), 0.86 (t, J = 6.9 Hz, 3H). **^{13}C NMR** (151 MHz, $CDCl_3$): δ 178.29, 170.03, 162.36, 157.67, 133.49, 130.17, 128.54, 119.35, 118.01, 116.98, 112.95, 60.92, 52.38, 49.88, 35.34, 35.10, 33.98, 31.89, 29.34, 28.91, 28.80, 26.01, 25.36, 24.49, 22.73, 20.33, 14.21. **$[\alpha]^{25}_D$** -55.35 (c 0.390, $CHCl_3$). **HRMS** Accurate Mass (ES+): Found 459.28528 (- 0.16 ppm), $C_{26}H_{39}O_5N_2$ (M + H+) requires 459.28535.

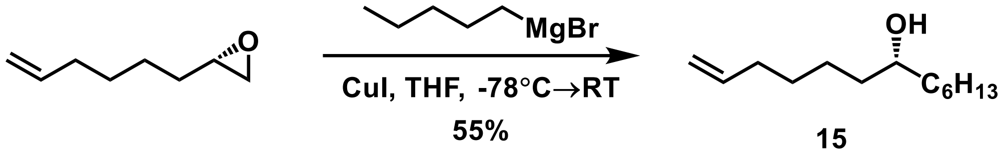

(R)-tridec-1-en-7-ol 15. A 50 mL round-bottom flask was flame-dried and purged thrice with argon. (S)-2-(hex-5-en-1-yl)oxirane[3] (0.589 g, 4.67 mmol, 1.0 eq.) was added and dissolved in THF (7 mL). Flame-dried CuI (0.444 g, 0.5 eq.) was added and the solution was cooled to -78 °C. Pentylmagnesium bromide solution (2.0M in THF, 11.7 mL, 5.0 eq.) was added dropwise and the dark blue reaction stirred 2.5 hours, allowing it to warm to room temperature. A saturated aqueous solution of NH_4Cl (8 mL) was added to quench the reaction, and the aqueous layer was extracted thrice with Et_2O. Combined organic layers were dried over anhydrous Na_2SO_4, filtered, and concentrated *in vacuo*. Crude product was purified by silica column chromatography (0-8% EtOAc/hexanes), to yield the title compound as a colorless oil (471.0 mg, 51% yield). **^{1}H NMR** (500 MHz, $CDCl_3$): δ 5.80 (ddt, J = 16.9, 10.2, 6.7 Hz, 1H), 4.99 (dq, J = 17.1, 2.1 Hz, 1H), 4.93 (ddt, J = 10.2, 2.3, 1.2 Hz, 1H), 3.65 – 3.52 (m, 1H), 2.06 (dt, J = 7.0, 6.9 Hz, 2H), 1.48 – 1.36 (m, 9H), 1.33 – 1.22 (m, 9H), 0.88 (t, J = 7.1 Hz, 3H). **^{13}C NMR** (151 MHz, $CDCl_3$): δ 138.91, 114.33, 71.92, 37.49, 37.28, 33.72, 31.83, 29.35, 28.95, 25.59, 25.12, 22.60, 14.06. **$[\alpha]^{25}_D$** +0.25 (c 0.79, $CHCl_3$). **HRMS** Accurate Mass (ES^+): Found 199.20570 (+ 0.30 ppm), $C_{13}H_{27}O$ (M + H^+) requires 199.20564.

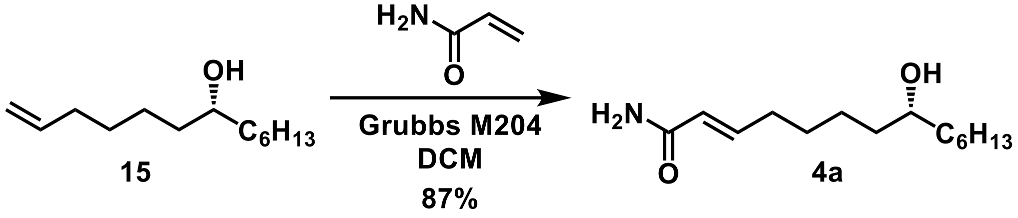

(R,E)-8-hydroxytetradec-2-enamide 4a. A 10 mL round-bottom flask fitted with reflux condenser was flame-dried and purged 3x with argon. Acrylamide (0.048 mL, 0.76 mmol, 3.0 eq.) was added, followed by Grubbs catalyst (M204, 21.0 mg, 0.025 mmol, 0.10 eq.). Alkene **15** (50.0 mg, 0.25 mmol, 1.0 eq.) was dissolved in DCM (3 mL) and added to the flask in one portion. The reaction was heated to reflux and stirred overnight. Crude product was directly purified by silica column chromatography (0-10 % MeOH/DCM), to yield the title compound as a yellow oil (52.7 mg, 87% yield). **^{1}H NMR** (500 MHz, $CDCl_3$): δ 6.79 (dt, J = 14.7, 6.9 Hz, 1H), 6.11 (d, J = 21.3 Hz, 2H), 5.83 (d, J = 15.4 Hz, 1H), 3.60-3.46 (m, 1H), 2.29 (br s, 1H), 2.16 (q, J = 6.3 Hz, 2H), 1.49 – 1.15 (m, 16H), 0.85 (t, J = 6.9 Hz, 3H). **^{13}C NMR** (151 MHz, $CDCl_3$): δ 168.48, 145.92, 122.97, 71.57, 37.50, 37.05, 31.89, 31.76, 29.29, 28.11, 25.58, 25.09, 22.52, 13.99. **$[\alpha]^{25}_D$** -1.6 (c 0.80, $CHCl_3$). **HRMS** Accurate Mass (ES^+): Found 242.21138 (- 0.32 ppm), $C_{14}H_{28}O_2N$ (M + H^+) requires 242.21146.

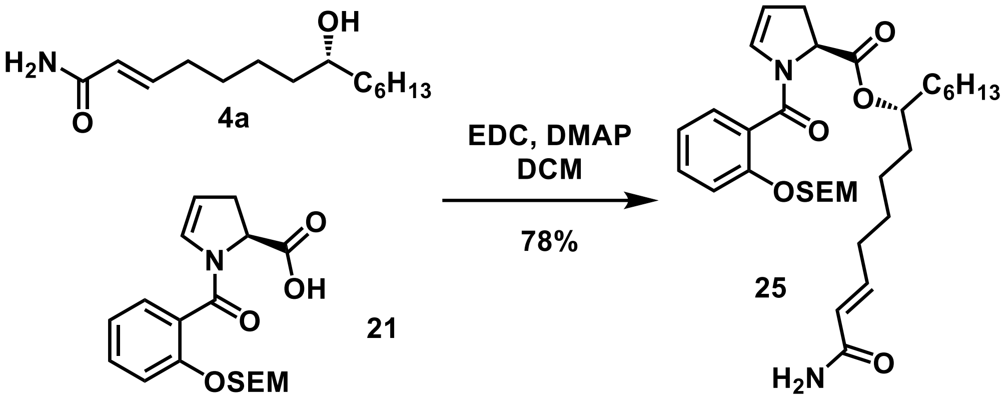

(R,E)-14-amino-14-oxotetradec-12-en-7-yl (S)-1-(2-((2-(trimethylsilyl)ethoxy)methoxy)benzoyl)-2,3-dihydro-1H-pyrrole-2-carboxylate 25. A 20 mL scintillation vial containing acid **21** (63.0 mg, 0.173 mmol, 1.0 eq.) was purged thrice with argon. DCM (6 mL) was added and the solution was cooled to 0 °C. EDC (49.8 mg, 0.260 mmol, 1.5 eq.) and DMAP (21.2 mg, 0.173 mmol, 1.0 eq.) were added and the yellow solution strred at 0 °C for 5 minutes. A solution of alcohol **4a** (83.7 mg, 0.347 mmol, 2.0 eq.) in DCM (3 mL) was added to the vial dropwise. The reaction stirred 16 hours, allowing it to warm to room temperature. The reaction was diluted with water, and the aqueous layer was extracted 5x with DCM. Combined organic layers were washed once with brine, back-extracted once with DCM, dried over anhydrous Na_2SO_4, filtered, and concentrated *in vacuo.* Crude product was purified by silica column chromatography (0-50% EtOAc/hexanes), yielding the title compound as a yellow oil (79.1 mg, 78% yield). **^{1}H NMR** (500 MHz, $CDCl_3$): δ 7.37 – 7.31 (m, 2H), 7.18 (d, J = 7.9 Hz, 1H), 7.03 (td, J = 7.5, 0.9 Hz, 1H), 6.78 (dt, J = 15.6, 6.8 Hz, 1H), 6.59 (s, 1H), 6.16 (dt, J = 4.3, 2.1 Hz, 1H), 5.88 (d, J = 15.7 Hz, 1H), 5.27 (d, J = 9.2 Hz, 1H), 5.23 (d, J = 7.1 Hz, 1H), 5.19 (d, J = 7.1 Hz, 1H), 5.05 (dt, J = 4.5, 2.6 Hz, 1H), 4.99 (ddd, J = 12.9, 7.3, 5.4 Hz, 1H), 4.95 (dd, J = 11.7, 4.9 Hz, 1H), 3.72 (dd, J = 9.2, 7.5 Hz, 2H), 3.12 (ddt, J = 16.7, 11.6, 2.4 Hz, 1H), 2.65 (ddt, J = 17.0, 4.8, 2.2 Hz, 1H), 2.18 (q, J = 6.5 Hz, 2H), 1.62 – 1.34 (m, 9H), 1.33 – 1.19 (m, 10H), 0.92 (dd, J = 9.4, 7.3 Hz, 2H), 0.85 (t, J = 7.1 Hz, 3H), -0.03 (s, 9H). **^{13}C NMR** (151 MHz, $CDCl_3$): δ 170.47, 168.63, 165.19, 153.55, 144.48, 131.27, 130.73, 128.68, 125.41, 123.86, 121.85, 115.11, 108.69, 93.13, 75.07, 66.54, 57.85, 34.39, 34.17, 34.07, 31.64, 31.51, 29.05, 27.40, 25.30, 24.16, 22.48, 17.95, 13.98, -1.48. **$[\alpha]^{25}_D$** -23.2 (c 0.97, $CHCl_3$). **HRMS** Accurate Mass (ES+): Found 587.35194 (+ 0.85 ppm), $C_{32}H_{51}O_6N_2{}^{28}Si$ (M + H+) requires 587.35109.

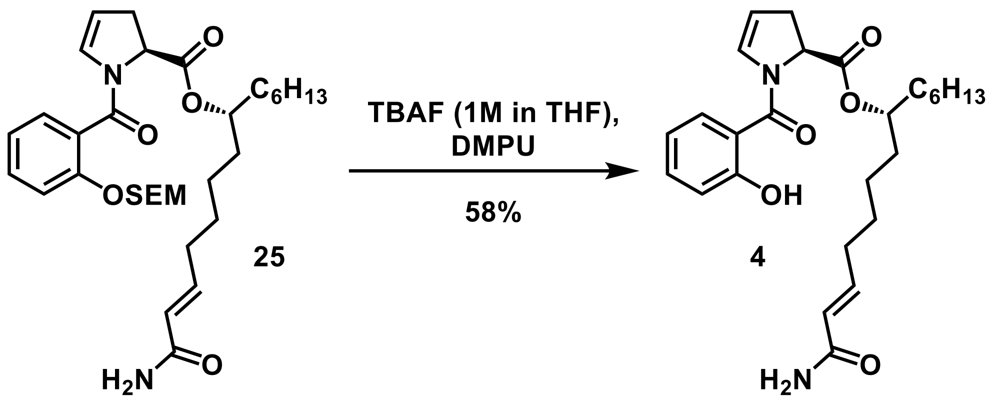

(R,E)-14-amino-14-oxotetradec-12-en-7-yl (S)-1-(2-hydroxybenzoyl)-2,3-dihydro-1H-pyrrole-2-carboxylate 4. A 20 mL scintillation vial containing silyl ether **25** (20.0 mg, 0.0341 mmol, 1.0 eq.) was purged thrice with argon. DMPU (0.68 mL) was added, followed by TBAF (1M in THF, 0.68 mL, 0.682 mmol, 20 eq.) dropwise, turning the reaction golden brown. The reaction was stirred at room temperature for 2 hours, then was quenched with saturated aqueous NH_4Cl (6 mL). The aqueous layer was extracted 3x with Et_2O, washed 5x with saturated aqueous NH_4Cl and 5x with brine, and dried over Na_2SO_4, then was filtered and concentrated *in vacuo.* Crude product was purified by silica column chromatography (0-6% MeOH/DCM) followed by HPLC, yielding the title compound as a colourless oil (9.1 mg, 58% yield). **^{1}H NMR** (600 MHz, $CDCl_3$): δ 9.69 (s, 1H), 7.41 (d, J = 7.7 Hz, 1H), 7.37 (t, J = 7.7 Hz, 1H), 7.00 (d, J = 8.3 Hz, 1H), 6.90 (t, J = 7.5 Hz, 1H), 6.82 (dt, J = 14.7, 6.9 Hz, 1H), 6.77 (s, 1H), 5.84 (d, J = 15.4 Hz, 1H), 5.75 (s, 1H), 5.48 (s, 1H), 5.27 (d, J = 3.3 Hz, 1H), 5.00 (dd, J = 11.3, 4.6 Hz, 1H), 4.98 (s, 1H), 3.13 (dd, J = 16.4, 11.8 Hz, 1H), 2.69 (d, J = 16.8 Hz, 1H), 2.20 (q, J = 6.7 Hz, 2H), 1.64 – 1.17 (m, 22H), 0.86 (t, J = 7.1 Hz, 3H). **^{13}C NMR** (151 MHz, $CDCl_3$): δ 170.90, 168.26, 167.31, 158.61, 145.87, 133.38, 130.83, 128.23, 123.08, 119.01, 117.91, 117.22, 110.65, 75.77, 59.32, 34.19, 33.89, 33.52, 31.74, 31.66, 29.07, 27.58, 25.27, 24.39, 22.53, 14.03. **$[\alpha]^{25}_D$** -25.1 (c 0.45, $CHCl_3$). **HRMS** Accurate Mass (ES+): Found 457.26983 (+ 0.28 ppm), $C_{26}H_{37}O_5N_2$ (M + H+) requires 457.26970.

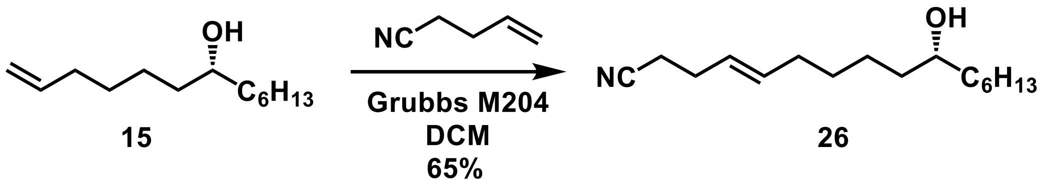

(R,E)-10-hydroxyhexadec-4-enenitrile 26.[4] A 10 mL round-bottom flask fitted with reflux condenser was flame-dried and purged thrice with argon. Homoallyl cyanide (142.2 mg, 1.753 mmol, 3.0 eq.) was added, followed by a solution of alcohol **15** (115.9 mg, 0.584 mmol, 1.0 eq.) in DCM (4 mL). Hoveyda-Grubbs catalyst (M720, 55.0 mg, 0.0877 mmol, 0.15 eq.) was added in one portion and the solution was heated to reflux and stirred 2 hours. Product was directly purified by silica column chromatography (0-40% EtOAc/hexanes), yielding title compound as a brown oil (95.3 mg, 65% yield). **^{1}H NMR** (500 MHz, $CDCl_3$): δ 5.87 – 5.24 (m, 2H), 3.54 (br s, 1H), 2.38 – 2.34 (m, 1H), 2.34 – 2.26 (m, 1H), 2.08 – 1.95 (m, 2H), 1.60 (s, 1H), 1.47 – 1.33 (m, 8H), 1.32 – 1.20 (m, 8H), 0.86 (t, J = 6.9 Hz, 3H). **^{13}C NMR** (126 MHz, $CDCl_3$): δ 134.16, 125.83,

117.29, 71.90, 37.59, 37.33, 32.41, 31.92, 29.44, 29.28, 28.44, 25.70, 25.14, 22.69, 17.82, 14.15. **[α]$^{25}_{D}$** -2.8 (c 0.638, $CHCl_3$). **HRMS** Accurate Mass (ES+): Found 252.23237 (+ 0.71 ppm), $C_{16}H_{30}ON$ (M + H+) requires 252.23219.

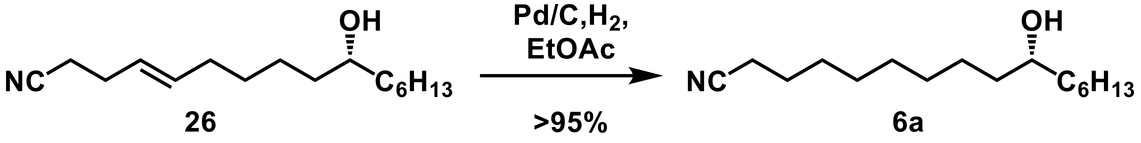

(R)-10-hydroxyhexadecanenitrile 6a.[5] A 20 mL scintillation vial was flame-dried and purged thrice with argon. Alkene **26** (30.0 mg, 0.119 mmol, 1.0 eq.) and palladium on activated carbon (5% Pd, 8.9 mg, 0.0042 mmol, 0.035 eq.) were added and dissolved in ethyl acetate (2 mL). The vial was evacuated and backfilled with hydrogen gas four times, then the reaction was left to stir under a hydrogen atmosphere (1 atm) for 4 hours. The dark grey suspension was filtered through Celite, eluting with 100% ethyl acetate to afford the title compound as a colourless oil (30.2 mg, >95% yield). **^{1}H NMR** (500 MHz, $CDCl_3$): δ 3.56 (dt, J = 8.4, 4.1 Hz, 1H), 2.32 (t, J = 7.1 Hz, 2H), 1.64 (p, J = 7.2 Hz, 2H), 1.49 – 1.34 (m, 10H), 1.34 – 1.21 (m, 14H), 0.87 (t, J = 6.9 Hz, 3H). **^{13}C NMR** (126 MHz, $CDCl_3$): δ 119.94, 72.05, 37.64, 37.52, 31.95, 29.64, 29.48, 29.37, 28.80, 28.73, 25.72, 25.68, 25.45, 22.72, 17.22, 14.19. **[α]$^{25}_{D}$** -1.5 (c 0.881, $CHCl_3$). **HRMS** Accurate Mass (ES+): Found 254.24775 (- 0.35 ppm), $C_{16}H_{32}ON$ (M + H+) requires 254.24784.

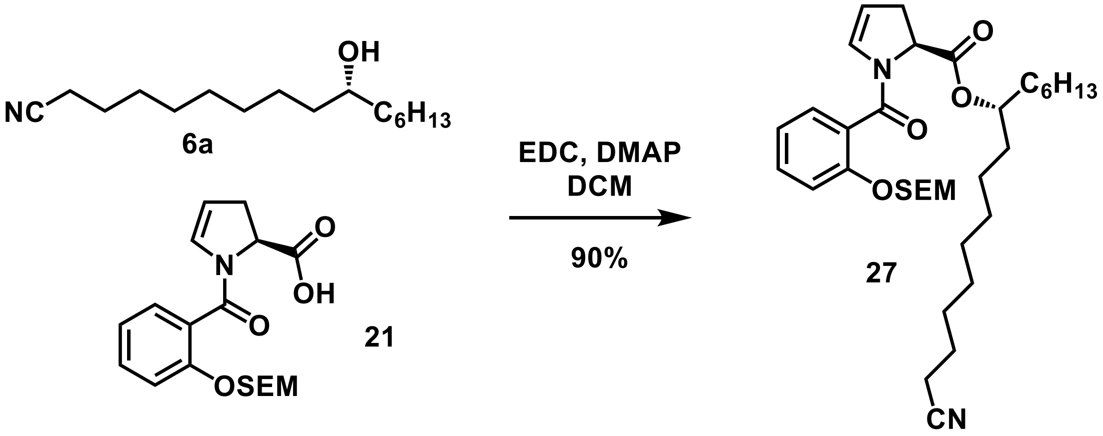

(R)-15-cyanopentadecan-7-yl (S)-1-(2-((2-(trimethylsilyl)ethoxy)methoxy)benzoyl)-2,3-dihydro-1H-pyrrole-2-carboxylate 27. A 20 mL scintillation vial containing acid **21** (41.0 mg, 0.113 mmol, 2.0 eq.) was purged thrice with argon. DCM (1.5 mL) was added and the reaction was cooled to 0 °C. EDC (21.6 mg, 0.113 mmol, 2 eq.) and DMAP (27.6 mg, 0.226 mmol, 4.0 eq.) were added and the orange solution stirred 5 minutes at 0 °C. A solution of alcohol **6a** (14.3 mg, 0.056 mmol, 1.0 eq.) in DCM (2 mL) was added and the reaction stirred overnight, allowing it to warm to room temperature. The reaction was diluted with water and the aqueous phase was extracted 5x with DCM. Combined organic layers were washed once with brine, back-extracted once with DCM, dried over anhydrous Na_2SO_4, filtered, and concentrated *in vacuo.* Product was purified by silica column chromatography (0-25% EtOAc/hexanes), affording the title compound as a yellow oil (30.4 mg, 90% yield). **^{1}H NMR** (500 MHz, $CDCl_3$): δ 7.39-7.32 (m, 2H), 7.19 (d, J = 8.0 Hz, 1H), 7.03 (t, J = 7.5 Hz, 1H), 6.16 (s, 1H), 5.25 – 5.19 (m, 2H), 5.02 (dt, J = 4.0, 1.9

Hz, 1H), 4.99 – 4.93 (m, 2H), 3.76 – 3.70 (m, 2H), 3.12 (ddt, J = 16.6, 11.6, 2.3 Hz, 1H), 2.66 (dt, J = 16.8, 2.2 Hz, 1H), 2.30 (t, J = 7.2 Hz, 2H), 1.64 – 1.51 (m, 6H), 1.44 – 1.36 (m, 3H), 1.26 (dd, J = 13.7, 6.9 Hz, 16H), 0.93 (dd, J = 9.4, 7.4 Hz, 2H), 0.86 (t, J = 6.6 Hz, 3H), -0.02 (s, 9H). **^{13}C NMR** (126 MHz, $CDCl_3$): δ 170.82, 164.98, 153.85, 131.23, 131.08, 129.00, 126.00, 121.98, 119.99, 115.30, 108.27, 93.38, 75.47, 66.62, 58.18, 34.39, 34.23, 31.85, 29.41, 29.30, 29.24, 28.72, 28.64, 25.45, 25.34, 25.15, 25.05, 22.69, 18.17, 17.16, 14.17, -1.29. **$[\alpha]^{25}_{D}$** -41.4 (c 1.047, $CHCl_3$). **HRMS** Accurate Mass (ES+): Found 599.38769 (+ 0.36 ppm), $C_{34}H_{55}O_5N_2{}^{28}Si$ (M + H+) requires 599.38748.

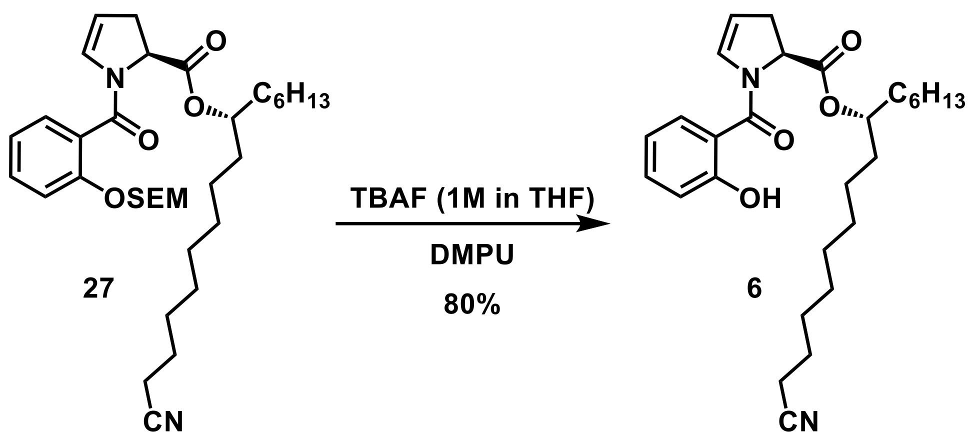

(R)-15-cyanopentadecan-7-yl (S)-1-(2-hydroxybenzoyl)-2,3-dihydro-1H-pyrrole-2-carboxylate 6. A 20 mL scintillation vial containing silyl ether **27** (13.6 mg, 0.0227 mmol, 1.0 eq.) was purged thrice with argon. DMPU (0.44 mL) was added, followed by TBAF (1M in THF, 0.44 mL, 0.444 mmol, 20 eq.) dropwise, turning the reaction golden brown. The reaction was stirred at room temperature for 2 hours, then was quenched with saturated aqueous NH_4Cl (6 mL). The aqueous layer was extracted 3x with Et_2O, washed 5x with saturated aqueous NH_4Cl and 5x with brine, and dried over Na_2SO_4, then was filtered and concentrated *in vacuo*. Crude product was purified by silica column chromatography (0-40% EtOAc/hexanes) followed by HPLC, yielding the title compound as a colourless oil (8.5 mg, 80% yield). **^{1}H NMR** (600 MHz, $CDCl_3$): δ 9.87 (s, 1H), 7.42 (d, J = 7.9 Hz, 1H), 7.37 (t, J = 8.5 Hz, 1H), 7.00 (d, J = 8.3 Hz, 1H), 6.89 (t, J = 7.5 Hz, 1H), 6.82 (s, 1H), 5.33 – 5.21 (m, 1H), 5.00 (dd, J = 11.2, 5.0 Hz, 1H), 4.96 (br s, 1H), 3.20 – 3.05 (m, 1H), 2.70 (d, J = 16.9 Hz, 1H), 2.31 (t, J = 7.2 Hz, 2H), 1.67 – 1.50 (m, 15H), 1.43 (dt, J = 15.0, 7.4 Hz, 3H), 1.36 – 1.17 (m, 22H), 0.86 (t, J = 7.1 Hz, 3H). **^{13}C NMR** (151 MHz, $CDCl_3$): δ 170.95, 167.58, 159.33, 133.60, 131.06, 128.47, 120.05, 118.98, 118.12, 116.98, 110.69, 75.95, 59.62, 34.24, 33.64, 31.83, 29.85, 29.81, 29.26, 29.18, 28.79, 28.66, 25.49, 25.38, 25.09, 22.69, 17.20, 14.20. **$[\alpha]^{25}_{D}$** -59.101 (c 0.089, $CHCl_3$). **HRMS** Accurate Mass (ES+): Found 469.30704 (+ 0.96 ppm), $C_{28}H_{41}O_4N_2$ (M + H+) requires 469.30608.

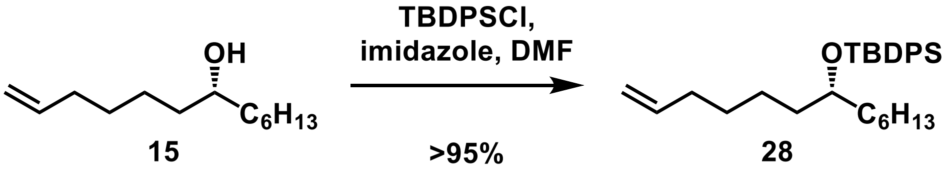

(R)-tert-butyldiphenyl(tridec-1-en-7-yloxy)silane 28. A 20 mL scintillation vial was flame-dried and purged thrice with argon. Alcohol **15** (100.0 mg, 0.504 mmol, 1.0 eq.), tert-butylchlorodiphenylsilane (0.197 mL, 0.756 mmol, 1.5 eq.), and imidazole (85.8 mg, 1.260 mmol, 2.5 eq.) were added sequentially and dissolved in DMF (5 mL). Solution stirred overnight at room temperature, then was concentrated *in vacuo*. Crude product was purified by silica column chromatography (0-10% EtOAc/hexanes), yielding the title compound as a colorless oil (217.7 mg, >95% yield). **^{1}H NMR** (500 MHz, $CDCl_3$): δ 7.72 – 7.69 (m, 4H), 7.45 – 7.41 (m, 2H), 7.40 – 7.36 (m, 4H), 5.77 (ddt, J = 16.9, 10.2, 6.7 Hz, 1H), 4.97 (dq, J = 17.1, 1.6 Hz, 1H), 4.93 (ddt, J = 10.2, 2.3, 1.2 Hz, 1H), 3.73 (p, J = 5.6 Hz, 1H), 1.97 (q, J = 6.9 Hz, 2H), 1.43 (dt, J = 11.7, 5.6 Hz, 4H), 1.31 – 1.19 (m, 9H), 1.20 – 1.12 (m, 4H), 1.08 (s, 9H), 0.88 (t, J = 7.2 Hz, 3H). **^{13}C NMR** (126 MHz, $CDCl_3$): δ 139.07, 135.97, 134.85, 129.37, 127.38, 114.16, 73.22, 36.35, 36.15, 33.72, 31.81, 29.37, 28.98, 27.12, 24.85, 24.42, 22.61, 19.44, 14.10. **$[\alpha]^{25}_D$** -0.40 (c 0.443, $CHCl_3$). **HRMS** Accurate Mass (ES+): Found 437.32317 (- 0.25 ppm), $C_{29}H_{45}O^{28}Si$ (M + H+) requires 437.32342.

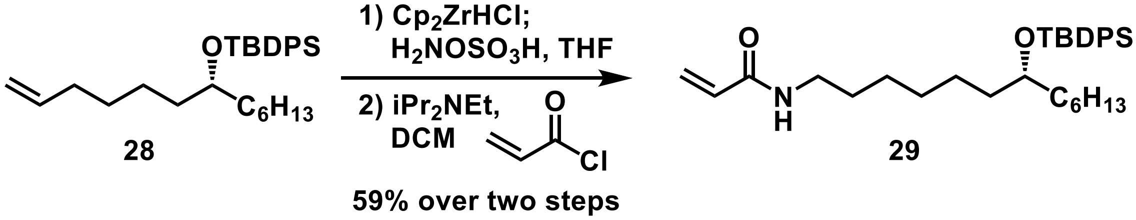

(R)-N-(7-((tert-butyldiphenylsilyl)oxy)tridecyl)acrylamide 29.[6] A 5 mL reaction tube containing 3A MS was flame-dried. Schwartz's reagent (Cp_2ZrHCl, 156.0 mg, 0.604 mmol, 1.5 eq.) was added and the reaction tube was purged thrice with argon. Alkene **28** (200.0 mg, 0.403 mmol, 1.0 eq.) was added, followed by THF (1.5 mL). The reaction tube was capped and the cloudy yellow reaction stirred at room temperature until the solution became homogenous (~35 minutes), at which point hydroxylamine-O-sulfonic acid (82.0 mg, 0.725 mmol, 1.8 eq.) was added in one portion. The now-colorless reaction continued to stir at RT for 30 minutes, then NaOH (1M in H_2O, 5 mL) was added followed by H_2O and the aqueous layer was extracted 3x with Et_2O. Combined organic layers were dried over anhydrous Na_2SO_4, filtered, and concentrated in vacuo to yield crude amine, which was purged thrice with argon. DCM (3 mL) was added and the solution was cooled to 0 °C. Diisopropylethylamine (0.175 mL, 1.01 mmol, 2.5 eq.) was added and the solution stirred 5 minutes. Acryloyl chloride (0.050 mL, 0.604 mmol, 1.5 eq.) was added dropwise, the reaction was warmed to room temperature and continued to stir four hours. The reaction was quenched with the addition of water (5 mL) and the aqueous layer was extracted 4x with DCM. Combined organic layers were washed once with brine, back-extracted once with DCM, dried over anhydrous Na_2SO_4, filtered, and concentrated *in vacuo*. Crude product was purified by silica column chromatography (0-40% EtOAc/hexanes), to yield the title compound as

a pale yellow oil (119.9 mg, 59% yield over two steps). **^{1}H NMR** (500 MHz, $CDCl_3$): δ 7.67 (dt, J = 8.0, 1.2 Hz, 4H), 7.43 – 7.38 (m, 2H), 7.38 – 7.33 (m, 4H), 6.27 (dd, J = 17.0, 1.4 Hz, 1H), 6.07 (dd, J = 17.0, 10.3 Hz, 1H), 5.63 (dd, J = 10.3, 1.4 Hz, 1H), 5.52 (s, 1H), 3.69 (p, J = 5.6 Hz, 1H), 3.28 (q, J = 7.2 Hz, 2H), 1.44 (dt, J = 14.6, 7.2 Hz, 2H), 1.38 (dt, J = 12.2, 6.3 Hz, 4H), 1.26 – 1.08 (m, 17H), 1.04 (s, 9H), 0.85 (t, J = 7.2 Hz, 3H). **^{13}C NMR** (126 MHz, $CDCl_3$): δ 165.57, 136.08, 134.92, 131.10, 129.50, 127.51, 126.32, 73.33, 39.73, 36.49, 36.33, 31.92, 29.61, 29.49, 29.45, 27.23, 26.97, 25.00, 24.88, 22.73, 19.57, 14.22. **$[\alpha]^{25}_D$** -0.97 (c 0.350, $CHCl_3$). **HRMS** Accurate Mass (ES+): Found 506.34492 (- 2.10 ppm), $C_{32}H_{48}O_2N^{28}Si$ (M + H+) requires 506.34598.

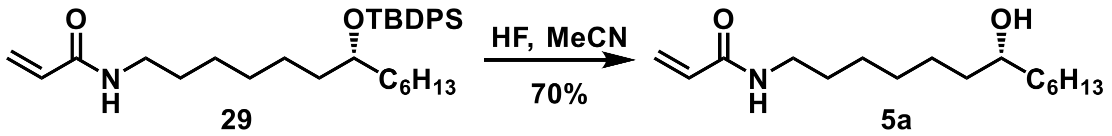

(R)-N-(7-hydroxytridecyl)acrylamide 5a. A 20 mL scintillation vial containing acrylamide **29** (119.9 mg, 0.236 mmol) was purged thrice with argon. Acetonitrile (2 mL) was added and the solution was cooled to 0 °C. Hydrofluoric acid (40% solution in H_2O, 1 mL) was then added and the reaction stirred for 16 hours, allowing it to warm to room temperature. The reaction was poured into a separatory funnel, diluted with water, and carefully neutralized with aqueous sodium bicarbonate. The aqueous layer was then extracted 4x with dichloromethane. Combined organic layers were washed once with brine, back-extracted once, and dried over anhydrous Na_2SO_4 before being filtered and concentrated *in vacuo*. Crude product was purified by silica column chromatography (10-70% EtOAc in hexanes), to yield the title compound as a white solid (44.7 mg, 70% yield). **^{1}H NMR** (600 MHz, $CDCl_3$): δ 6.27 (d, J = 17.0 Hz, 1H), 6.07 (ddd, J = 16.9, 10.3, 1.6 Hz, 1H), 5.62 (d, J = 10.3 Hz, 1H), 5.57 (s, 1H), 3.57 (br s, 1H), 3.35 – 3.30 (m, 2H), 1.64 (s, 2H), 1.57 – 1.50 (m, 2H), 1.47 – 1.24 (m, 22H), 0.88 (t, J = 6.2 Hz, 3H). **^{13}C NMR** (151 MHz, $CDCl_3$): δ 165.63, 131.06, 126.39, 72.06, 39.69, 37.70, 37.45, 31.98, 29.64, 29.51, 29.39, 26.98, 25.77, 25.64, 22.76, 14.23. **$[\alpha]^{25}_D$** -1.8 (c 0.415, $CHCl_3$). **HRMS** Accurate Mass (ES+): Found 268.22815 (- 0.19 ppm), $C_{16}H_{30}O_2N$ (M - H+) requires 268.22820.

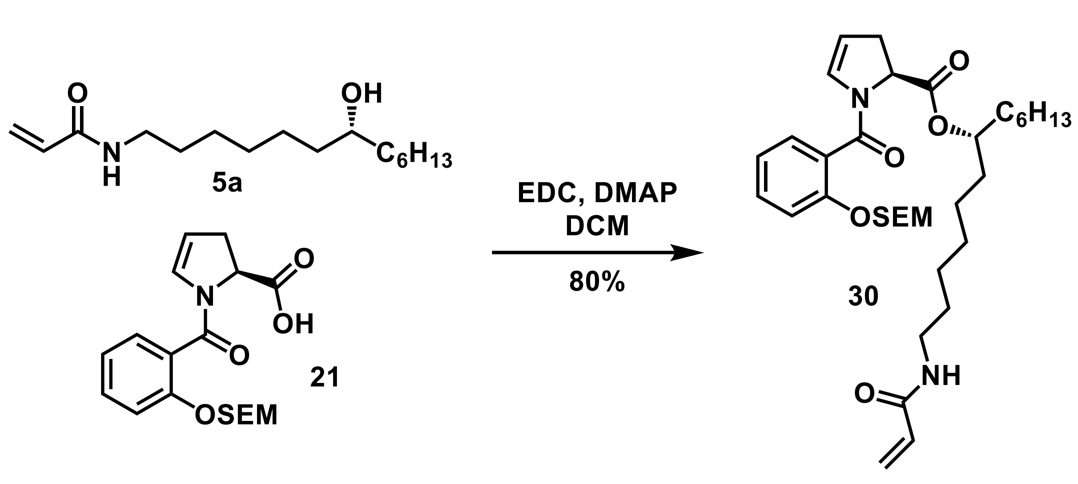

(R)-1-acrylamidotridecan-7-yl (S)-1-(2-((2-(trimethylsilyl)ethoxy)methoxy)benzoyl)-2,3-dihydro-1H-pyrrole-2-carboxylate 30. A 20 mL scintillation vial containing acid **21** was purged 3x with argon. DCM (1.5 mL) was added and the solution was cooled to 0 °C. EDC (36.0 mg, 0.19 mmol, 2.5 eq.) and DMAP (36.0 mg, 0.30 mmol, 4 eq.) were added and the reaction stirred 5 minutes at 0 °C. Acrylamide **5a** was dissolved in DCM (2 mL) and transferred to the reaction vial. The reaction was warmed to RT and stirred 24 hours, then was diluted with water and extracted 5x with DCM. Combined organic layers were washed once with brine, back-extracted once with DCM, dried over anhydrous Na_2SO_4, filtered, and concentrated *in vacuo*. Crude product was purified by silica column chromatography (0-40% EtOAc/hexanes), to yield the title compound as a yellow oil (36.4 mg, 80% yield). **^{1}H NMR** (500 MHz, $CDCl_3$): δ 7.37 – 7.29 (m, 2H), 7.19 (d, J = 8.3 Hz, 1H), 7.03 (t, J = 7.5 Hz, 1H), 6.57 (s, 1H), 6.16 (dt, J = 4.1, 2.0 Hz, 1H), 6.12 (dd, J = 17.0, 1.6 Hz, 1H), 5.80 (dd, J = 17.0, 10.3 Hz, 1H), 5.36 (dd, J = 10.3, 1.6 Hz, 1H), 5.24 – 5.18 (m, 2H), 5.05 (dt, J = 4.5, 2.5 Hz, 1H), 5.03 – 4.99 (m, 1H), 4.97 (dd, J = 11.6, 4.7 Hz, 1H), 3.72 (t, J = 8.10 Hz, 2H), 3.25 (ddt, J = 28.3, 13.2, 6.4 Hz, 2H), 3.13 (ddt, J = 16.5, 11.6, 2.3 Hz, 1H), 2.67 (ddt, J = 17.0, 4.7, 2.5 Hz, 1H), 1.99 (s, 1H), 1.62 – 1.46 (m, 6H), 1.42 – 1.18 (m, 16H), 0.92 (dd, J = 9.2, 7.5 Hz, 2H), 0.86 (t, J = 6.9 Hz, 3H), -0.02 (s, 9H). **^{13}C NMR** (126 MHz, $CDCl_3$): δ 170.64, 165.73, 165.34, 153.75, 131.38, 131.30, 130.99, 128.87, 125.77, 125.29, 122.08, 115.25, 108.73, 93.32, 75.20, 66.68, 58.03, 39.27, 34.57, 34.38, 34.08, 31.84, 29.27, 28.63, 28.33, 26.42, 25.50, 24.56, 22.68, 18.16, 14.17, -1.29. **$[\alpha]^{25}_D$** -39.6 (c 0.985, $CHCl_3$). **HRMS** Accurate Mass (ES+): Found 615.38291 (+ 0.84 ppm), $C_{34}H_{55}O_6N_2{}^{28}Si$ (M + H+) requires 615.38239.

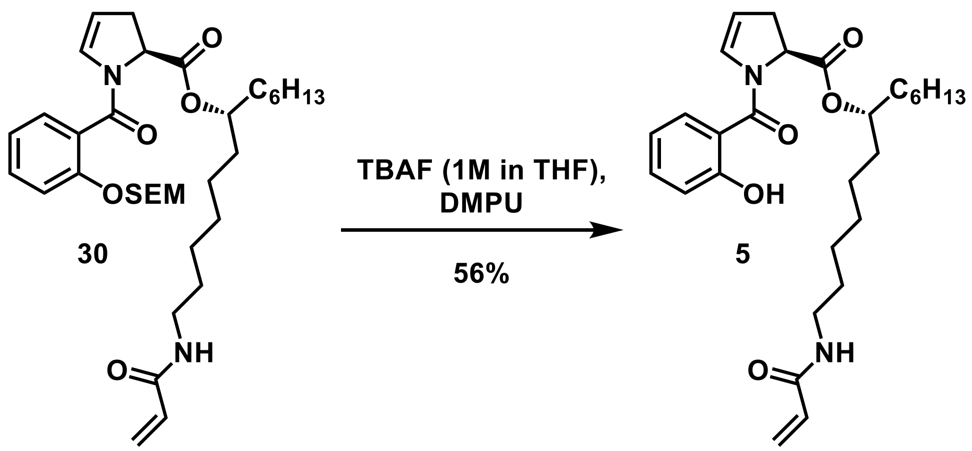

(R)-1-acrylamidotridecan-7-yl (S)-1-(2-hydroxybenzoyl)-2,3-dihydro-1H-pyrrole-2-carboxylate 5. A 20 mL scintillation vial containing silyl ether **30** (17.2 mg, 0.0280 mmol, 1.0 eq.) was purged thrice with argon. DMPU (0.56 mL) was added, followed by TBAF (1M in THF, 0.56 mL, 0.559 mmol, 20 eq.) dropwise, turning the reaction golden brown. The reaction was stirred at room temperature for 2 hours, then was quenched with saturated aqueous NH_4Cl (6 mL). The aqueous layer was extracted 3x with Et_2O, washed 5x with saturated aqueous NH_4Cl and 5x with brine, and dried over Na_2SO_4, then was filtered and concentrated *in vacuo*. Crude product was purified by silica column chromatography (10-55% EtOAc/hexanes) followed by HPLC, yielding the title compound as a white solid (7.6 mg, 56% yield). **^{1}H NMR** (600 MHz, $CDCl_3$): δ

9.88 (s, 1H), 7.42 (d, J = 7.8 Hz, 1H), 7.37 (t, J = 7.8 Hz, 1H), 6.99 (d, J = 8.3 Hz, 1H), 6.89 (t, J = 7.5 Hz, 1H), 6.80 (s, 1H), 6.24 (d, J = 17.0 Hz, 1H), 6.05 (dd, J = 17.0, 10.3 Hz, 1H), 5.85 (s, 1H), 5.57 (d, J = 11.4 Hz, 1H), 5.28 (d, J = 4.1 Hz, 1H), 5.00 (dd, J = 11.2, 4.9 Hz, 2H), 3.30 (ddq, J = 26.3, 13.3, 6.7 Hz, 2H), 3.12 (dd, J = 16.7, 11.5 Hz, 1H), 2.69 (d, J = 17.1 Hz, 1H), 1.69 (s, 7H), 1.59 – 1.47 (m, 8H), 1.37 – 1.18 (m, 24H), 0.86 (t, J = 7.1 Hz, 4H). **^{13}C NMR** (151 MHz, CDCl3): δ 171.06, 167.50, 165.71, 158.99, 133.60, 131.19, 130.99, 128.41, 126.09, 119.14, 118.05, 117.16, 110.89, 75.98, 59.60, 39.64, 34.39, 34.26, 33.63, 31.83, 29.85, 29.24, 28.91, 26.86, 25.45, 24.92, 22.69, 14.20. **$[\alpha]^{25}_D$** -13.419 (c 0.313, $CHCl_3$). **HRMS** Accurate Mass (ES+): Found 485.30071 (- 0.29 ppm), $C_{28}H_{41}O_5N_2$ (M + H+) requires 485.30100.

2. Biology

Table S1. Primers used in generation of the novel PA14 efflux deletion strain

Primers Name	Sequence
pEX18gm Gibson Assembly universal F	ggtcatagctgtttcctgtgtg
pEX18gm Gibson Assembly Universal R	caacgtcgtgactgggaaaac
mexCD-oprJ KO GA F	ccagtcacgacgttgGCTTCCAGGTAGGACTGC
mexCD-oprJ KO GA R	ggaaacagctatgaccCACCATGGGAGAACTGGC
mexCD-oprJ outside seq F	GATGAACAGTTCGGTGAACACGG
mexCD-oprJ outside seq R	CAGGACCAGCGTCTGCTCAAG
mexCD-oprJ inside seq F	CCATCCTGGTGGTCTTCCTG
mexCD-oprJ inside seq R	GATACGCCGAATGCAGGTTTG
mexEF-OprN KO GA F	ccagtcacgacgttgCGTCGACGAGGAACTGGAG
mexEF-OprN KO GA R	ggaaacagctatgaccCCTTGCAACAGCTCAATCAC
mexEF-OprN outside seq F	CAGCTGACCCTCGACGACTAC
mexEF-OprN outside seq R	CCAACCTGCAAGTCGACCTG
mexEF-OprN inside seq F	GATCGTCGAGTTCGCCAAGG
mexEF-OprN inside seq R	GACCAACTGGTTCAGGGTCG
opmH KO GA F	ccagtcacgacgttgCCTTGCGCGAGTTCGGGA
opmH KO GA R	ggaaacagctatgaccGACCTGCTGATCCTCAACTTTCCG
ompH outside seq F	GAGGGAGATTTCCCGCACC
ompH outside seq R	GATCCCGACTACCTGGTCTAC
ompH inside seq F	CTGGTGACCCTGACCAACC
ompH inside seq R	GCTCGACGTCGGTATTCAC
mexXY KO GA F	ccagtcacgacgttgCTTGAGGTAGAGGATCTCCAG
mexXY KO GA R	ggaaacagctatgaccGTTGTTCCTCACCGATCTG

mexXY outside seq F	CGATTGCAGATAGATGCTGG
mexXY outside seq R	GTGATCGACATCGACGATCC
mexXY inside seq F	GCAGATCGATCCGATCTACG
mexXY inside seq R	GTAGCGTTCTCCGTCACTG
mexGHI - opmD KO GA F	ccagtcacgacgttgCCACATATGGCAAGTCCTGC
mexGHI - opmD KO GA R	ggaaacagctatgaccGAAGGCGAGCAACCTGGC
mexGHI - opmD outside seq F	CTAAGCGGTCATCCGCACTAC
mexGHI - opmD outside seq R	CGAAGACTTCTACAGCTACCTG
mexGHI - opmD inside seq F	CAACCGCTTCGGCATGGAAG
mexGHI - opmD inside seq R	GTTCATCGTCGCGTAGCCCAG
mexJK KO GA F	ccagtcacgacgttgCCATCTCGTCGATCACCTGC
mexJK KO GA R	ggaaacagctatgaccGGACGTGCGCATCGAACTG
mexJK outside seq F	CTTGACCAGCATGAAGAAGTG
mexJK outside seq R	GACGCTGTCATGTTCCGACTC
mexJK inside seq F	GAGCTGCTGCTGGACATCAAG
mexJK inside seq R	CTCGGCAGCAACGACAGGTC
oprD KO GA F	ccagtcacgacgttgCTCGATGGCAACCAACCCTTG
oprD KO GA R	ggaaacagctatgaccGCAGGCAATGAACGCGGC
oprD outside seq F	CAAACGCATTCGCCACAGAC
oprD outside seq R	GGCCATCGATGATGAGGAGTC
oprD inside seq F	GATGCCTTCGGCTACCTCGG
oprD inside seq R	CGCATCCAGAGTGTAGGCTGC
mexAB KO GA F	ccagtcacgacgttgCATCACCGGCAACCTGAC
mexAB KO GA R	ggaaacagctatgaccGGACGAAGAGAAGCTGCTC
mexAB outside seq F	CATCAGGTCGGGATTCACG
mexAB outside seq R	GGAAGAGTTCCTCGACGAC
mexAB inside seq F	CATGTTCGTTCACGCGCAG
mexAB inside seq R	CATCTGCTGCTCGATCACCTG

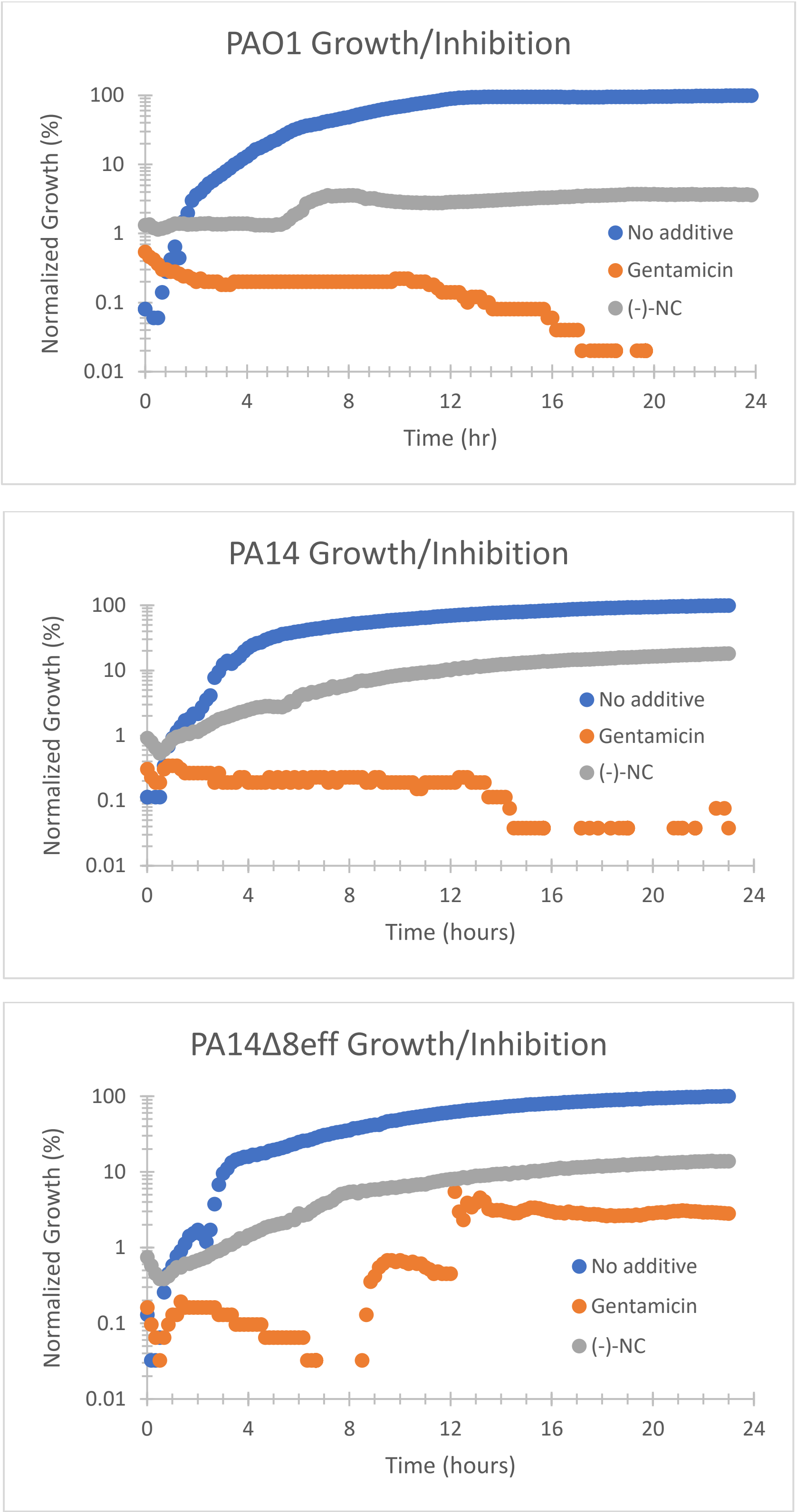

Figure S1. Normalized growth and inhibition curves for all *PA* strains tested. Gentamicin and dehydroxypromysalin **(-)-NC** were tested at 100 µM for all strains.

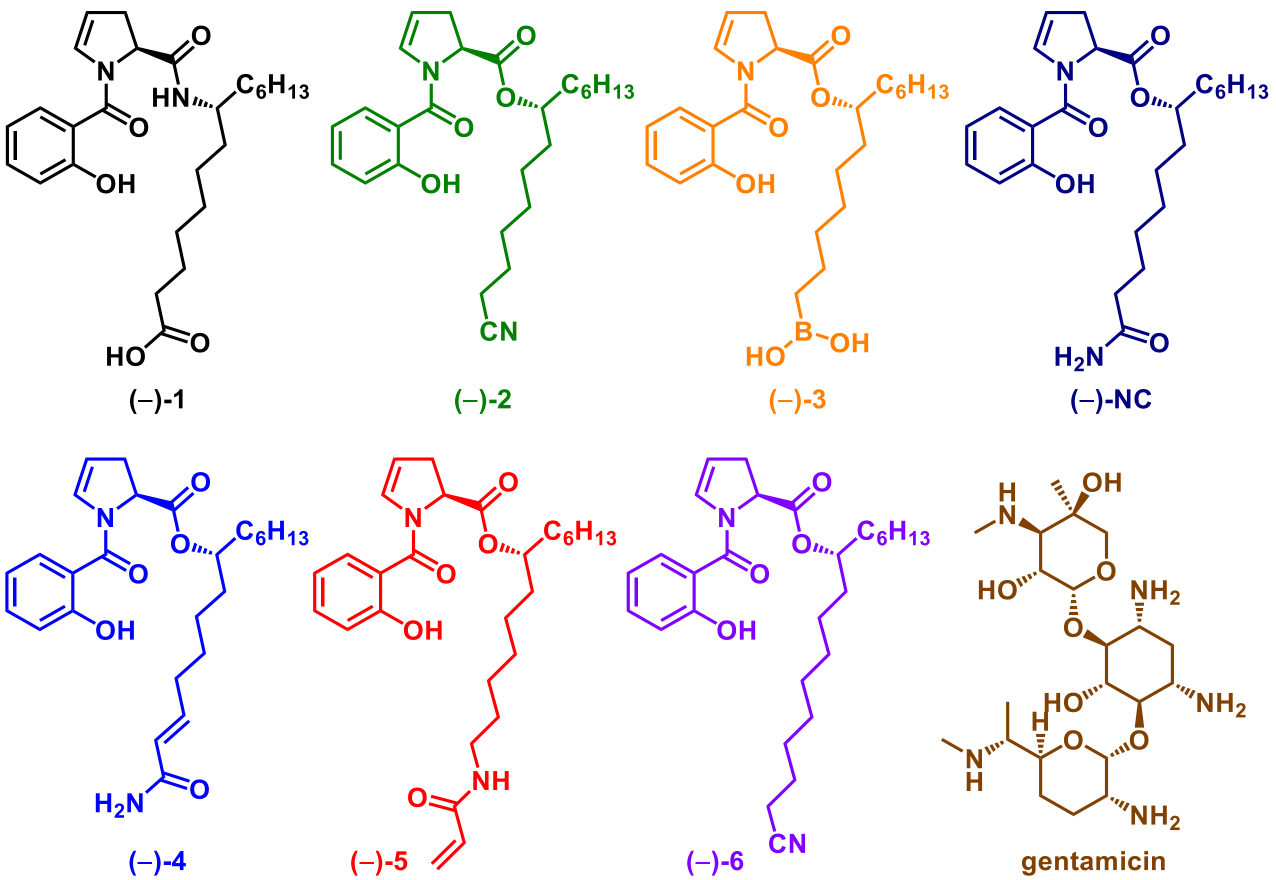

Figure S2. Six compounds tested for biological activity against *Pseudomonas aeruginosa* PA14, PA01, and PA14 efflux knockout strain. Gentamicin and promysalin analogue **NC** served as positive controls.

PA14		
Analogue	IC_{50}	R^2
5	5.58	0.938
6	3.67	0.970
4	5.24	0.971
2	1.58	0.977
3	21.6	0.962
1	>250	N/A
NC	0.516	0.974
gentamicin	6.16	0.981

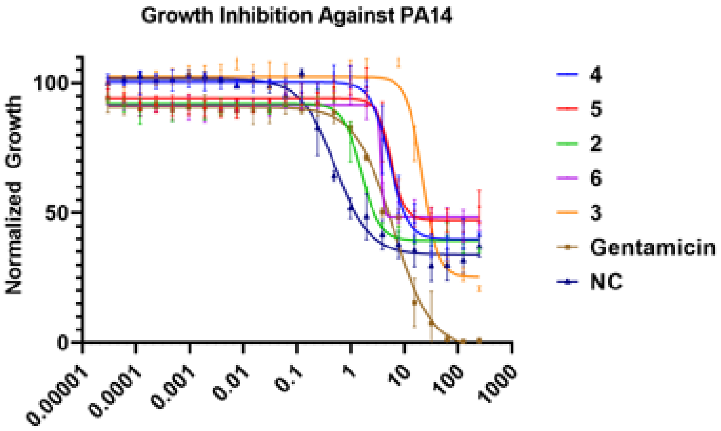

PAO1		
Analogue	IC_{50}	R^2
5	7.69	0.917
6	2.56	0.928
4	15.2	0.914
2	2.04	0.878
3	42.2	0.670
1	>250	N/A
NC	3.29	0.920
gentamicin	4.90	0.992

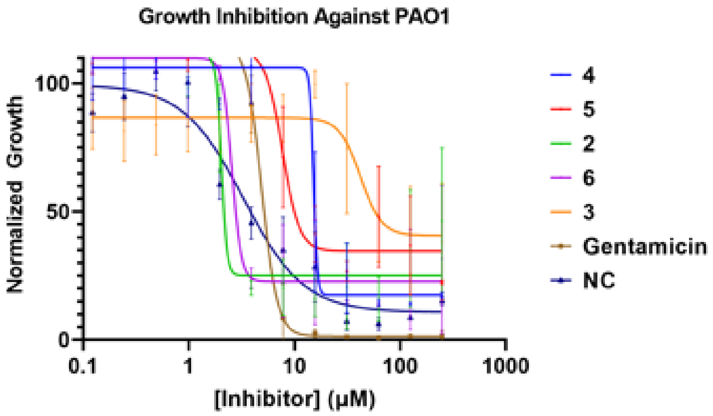

PA14 efflux		
Analogue	IC_{50}	R^2
5	0.388	0.950
6	1.28	0.974
4	0.00299	0.920
2	0.267	0.977
3	N.T	N/A
1	N.T	N/A
NC	7.44E-05	0.942
gentamicin	3.50	0.988

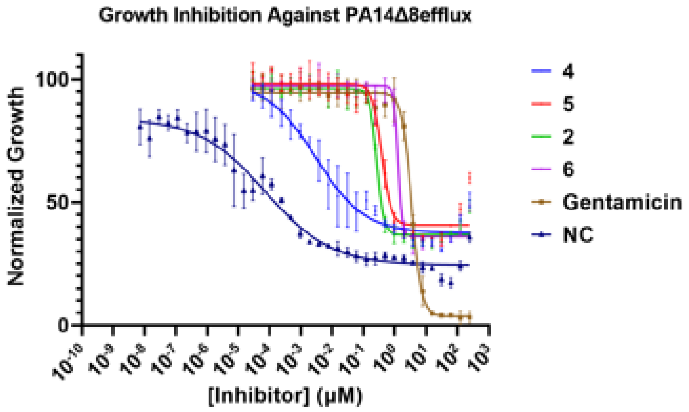

Table S2. IC_{50} values (μM) for all active compounds tested.

Figure S3. IC_{50} curves (μM) for all active compounds tested. Error bars represent ± 1 SEM. Analogue **1** was omitted from the curves because its IC_{50} was not below 250 μM.

3. References

1. Steele, A. D.; Knouse, K. W.; Keohane, C. E.; Wuest, W. M. Total Synthesis and Biological Investigation of (−)-Promysalin. *J. Am. Chem. Soc.* **2015**, *137* (23), 7314–7317. https://doi.org/10.1021/jacs.5b04767.

2. Steele, A. D.; Keohane, C. E.; Knouse, K. W.; Rossiter, S. E.; Williams, S. J.; Wuest, W. M. Diverted Total Synthesis of Promysalin Analogs Demonstrates That an Iron-Binding Motif Is Responsible for Its Narrow-Spectrum Antibacterial Activity. *J. Am. Chem. Soc.* **2016**, *138* (18), 5833–5836. https://doi.org/10.1021/jacs.6b03373.

3. Kubizna, P.; Špánik, I.; Kožíšek, J.; Szolcsányi, P. Synthesis of 2,6-Disubstituted Piperidine Alkaloids from Ladybird Beetles Calvia 10-Guttata and Calvia 14-Guttata. *Tetrahedron* **2010**, *66* (13), 2351–2355. https://doi.org/10.1016/j.tet.2010.01.106.

4. Abel, G.; Nguyen, K.; Viamajala, S.; Varanasi, S. Cross-Metathesis Approach to Produce Precursors of Nylon 12 and Nylon 13 from Methyl Oleate. *RSC Advances* **2012**, 7.

5. Montes Vidal, D.; von Rymon-Lipinski, A.-L.; Ravella, S.; Groenhagen, U.; Herrmann, J.; Zaburannyi, N.; Zarbin, P. H. G.; Varadarajan, A. R.; Ahrens, C. H.; Weisskopf, L.; Müller, R.; Schulz, S. Long-Chain Alkyl Cyanides: Unprecedented Volatile Compounds Released by Pseudomonas and Micromonospora Bacteria. *Angewandte Chemie International Edition* **2017**, *56* (15), 4342–4346. https://doi.org/10.1002/anie.201611940.

6. Strom, A. E.; Hartwig, J. F. One-Pot Anti-Markovnikov Hydroamination of Unactivated Alkenes by Hydrozirconation and Amination. *J Org Chem* **2013**, *78* (17), 8909–8914. https://doi.org/10.1021/jo401498w.

7. Schweizer, H. P.; Hoang, T. T. An Improved System for Gene Replacement and XylE Fusion Analysis in Pseudomonas Aeruginosa. *Gene* **1995**, *158* (1), 15–22. https://doi.org/10.1016/0378-1119(95)00055-b.

8. Hmelo, L. R.; Borlee, B. R.; Almblad, H.; Love, M. E.; Randall, T. E.; Tseng, B. S.; Lin, C.; Irie, Y.; Storek, K. M.; Yang, J. J.; Siehnel, R. J.; Howell, P. L.; Singh, P. K.; Tolker-Nielsen, T.; Parsek, M. R.; Schweizer, H. P.; Harrison, J. J. Precision-Engineering the Pseudomonas Aeruginosa Genome with Two-Step Allelic Exchange. *Nat Protoc* **2015**, *10* (11), 1820–1841. https://doi.org/10.1038/nprot.2015.115.

9. Avissar, Y. J.; Beale, S. I. Identification of the Enzymatic Basis for Delta-Aminolevulinic Acid Auxotrophy in a HemA Mutant of Escherichia Coli. *J Bacteriol* **1989**, *171* (6), 2919–2924. https://doi.org/10.1128/jb.171.6.2919-2924.1989.

4. Appendix: Spectral Data

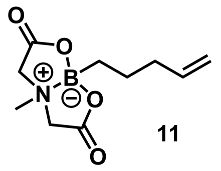

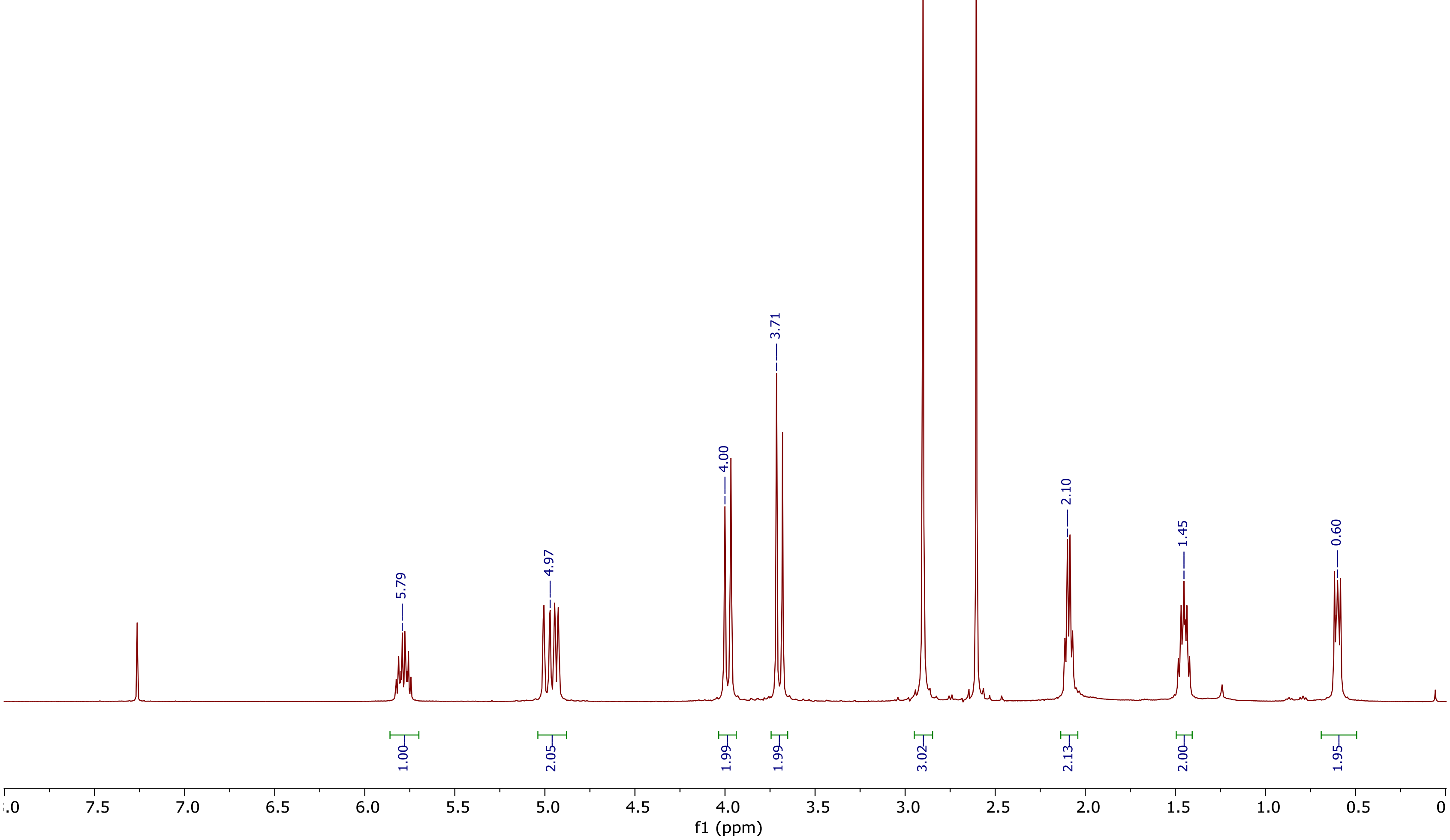

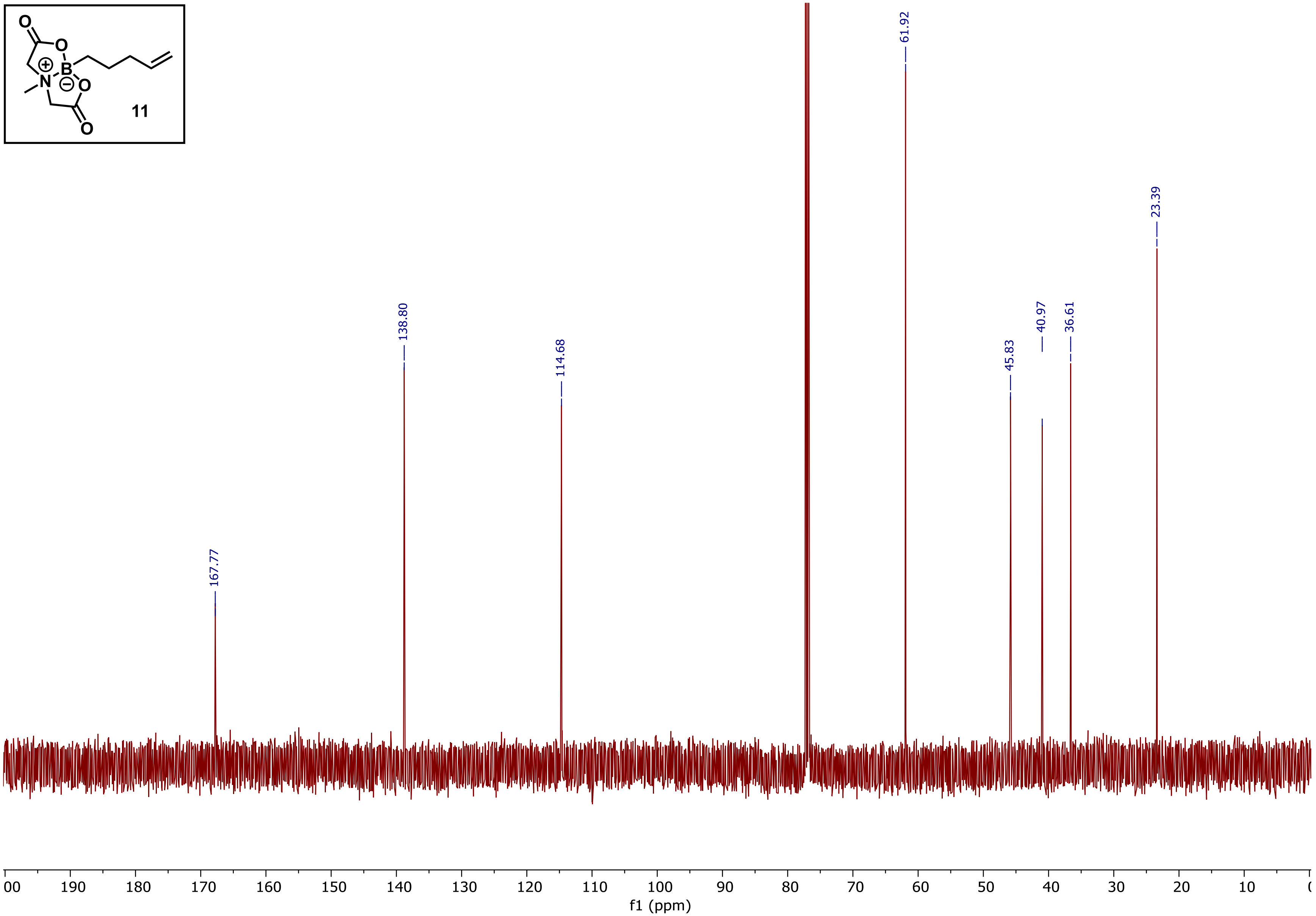
11
167.77
138.80
114.68
61.92
45.83
40.97
36.61
23.39
00 190 180 170 160 150 140 130 120 110 100 90 80 70 60 50 40 30 20 10
f1 (ppm)

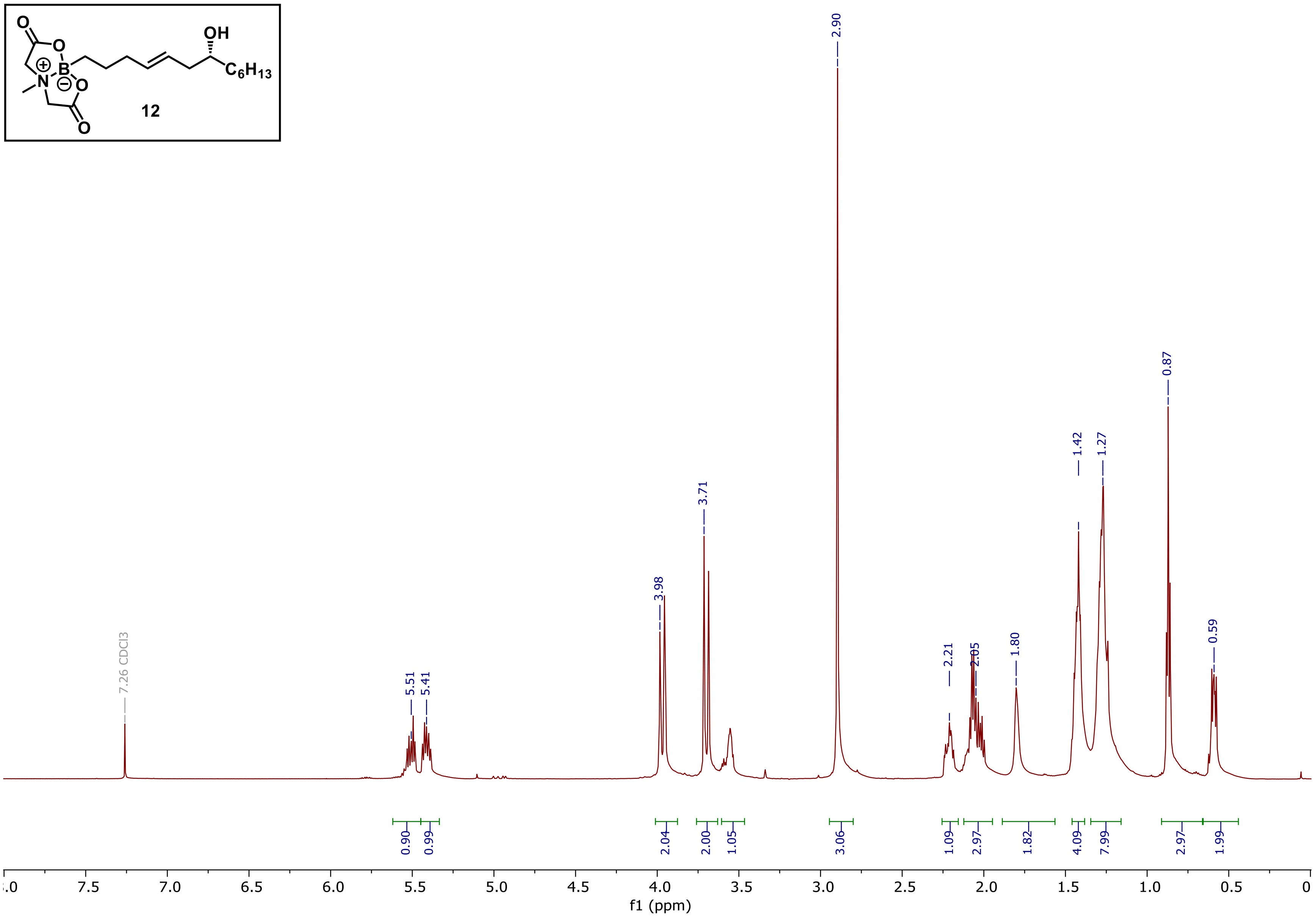
OH
C6H13
12
7.26 CDCl3
5.51
5.41
3.98
3.71
2.90
2.21
2.05
1.80
1.42
1.27
0.87
0.59
0.90
0.99
2.04
2.00
1.05
3.06
1.09
2.97
1.82
4.09
7.99
2.97
1.99
7.5
7.0
6.5
6.0
5.5
5.0
4.5
4.0
3.5
3.0
2.5
2.0
1.5
1.0
0.5
0
f1 (ppm)

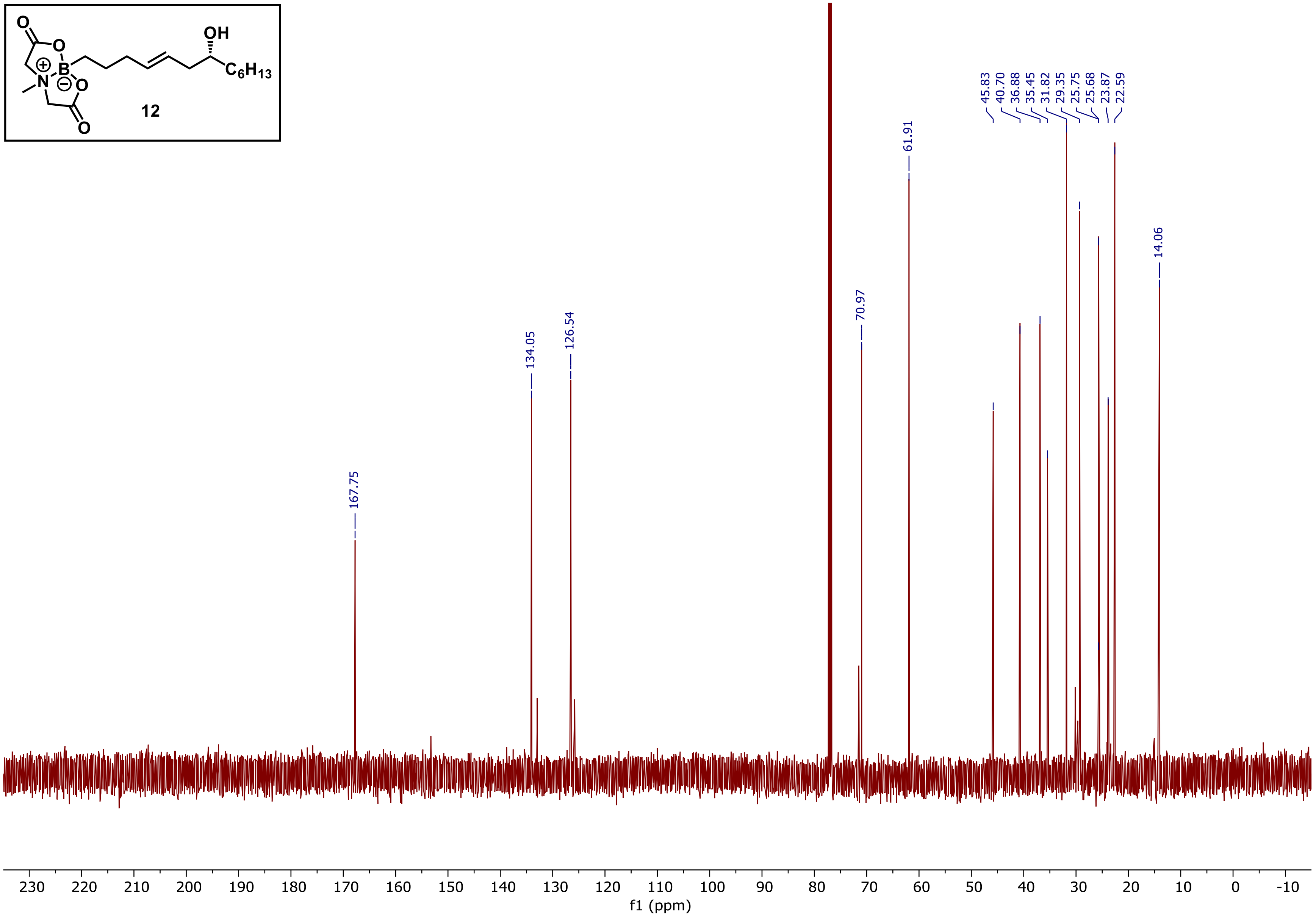
OH
C6H13
12
167.75
134.05
126.54
70.97
61.91
45.83
40.70
36.88
35.45
31.82
29.35
25.75
25.68
23.87
22.59
14.06
230 220 210 200 190 180 170 160 150 140 130 120 110 100 90 80 70 60 50 40 30 20 10 0 -10
f1 (ppm)

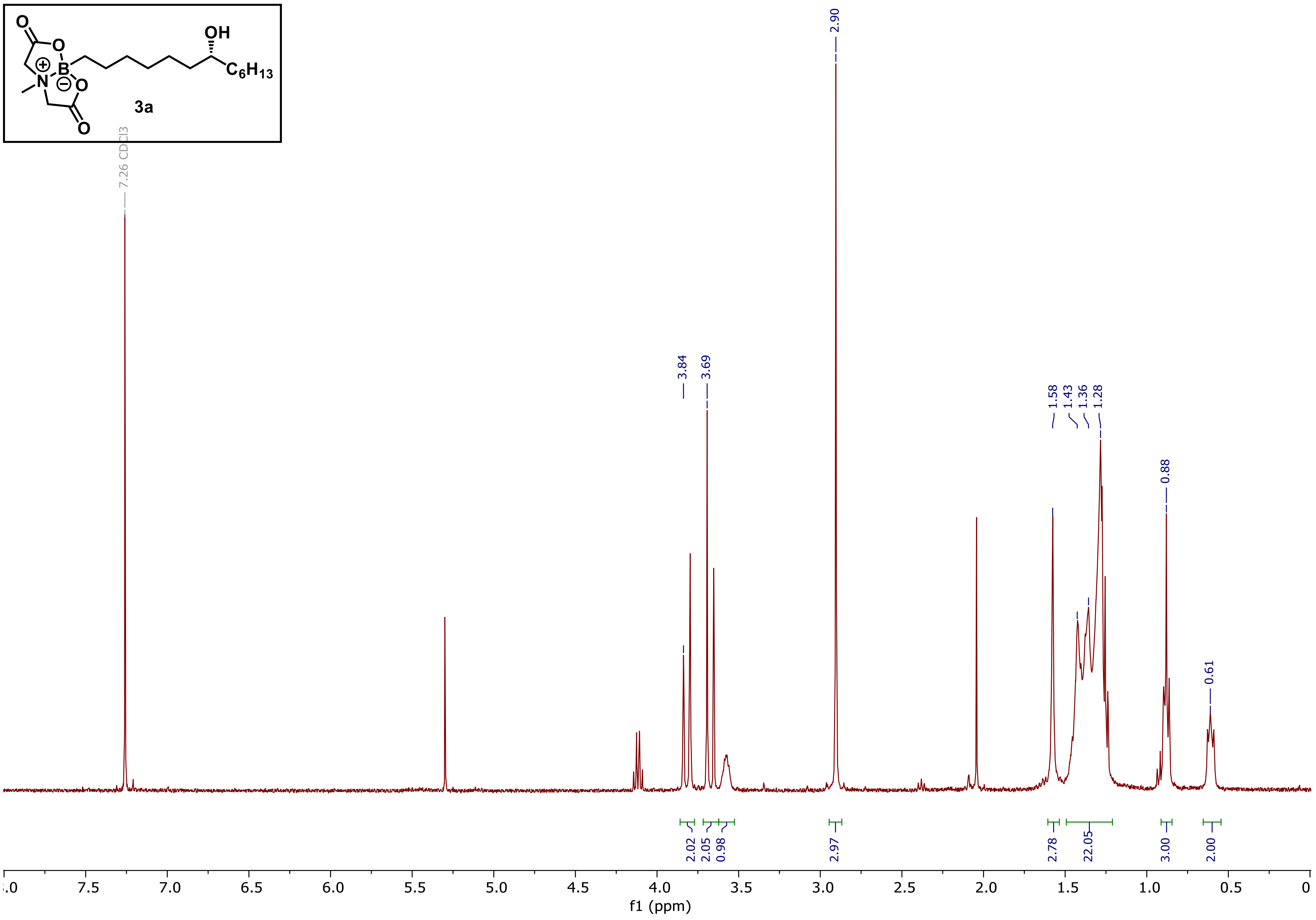

OH
C_6H_{13}
3a
7.26 CDCl3
2.90
3.84
3.69
1.58
1.43
1.36
1.28
0.88
0.61
2.02
2.05
0.98
2.97
2.78
22.05
3.00
2.00
7.5 7.0 6.5 6.0 5.5 5.0 4.5 4.0 3.5 3.0 2.5 2.0 1.5 1.0 0.5 0
f1 (ppm)

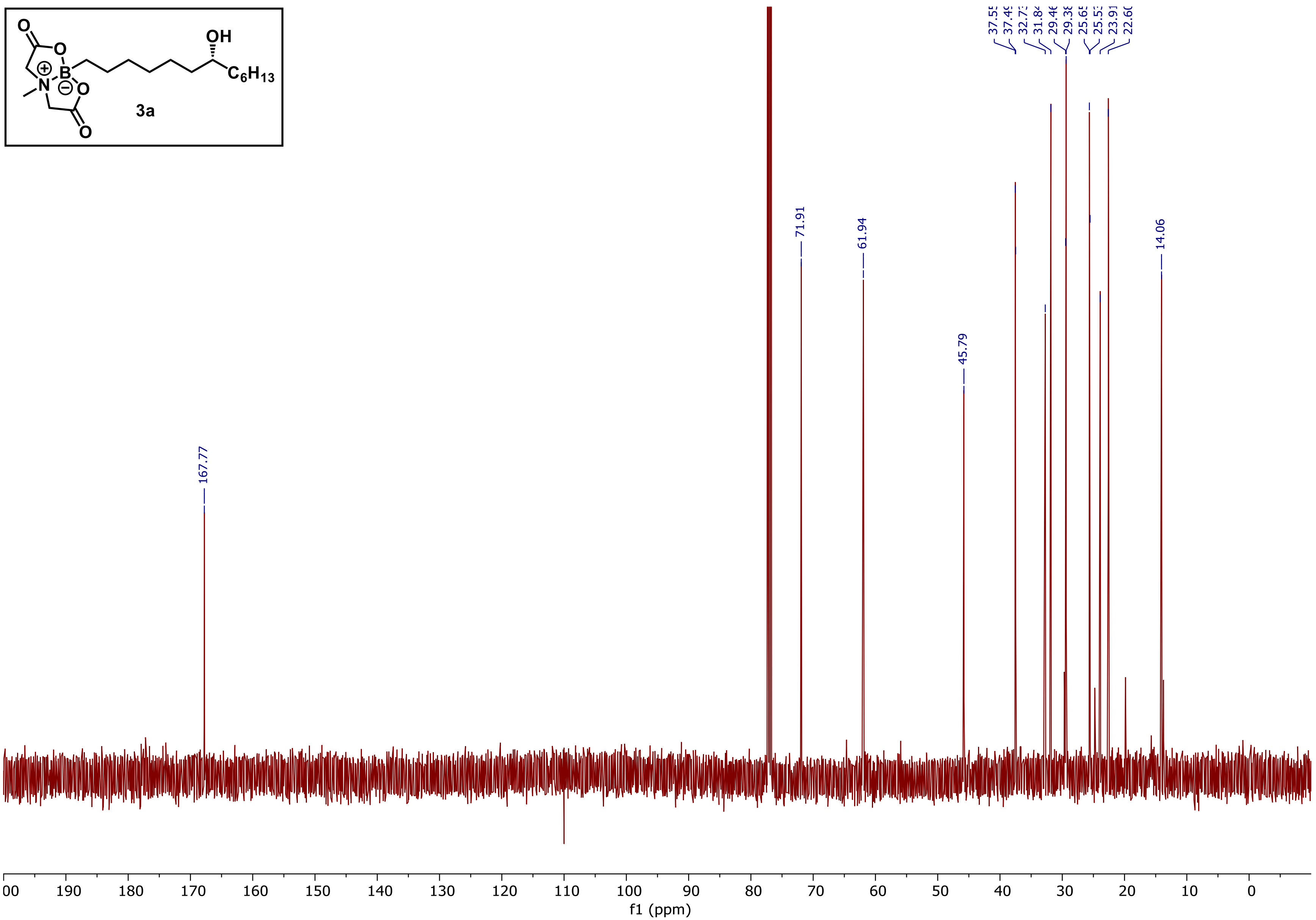
OH
C_6H_{13}
3a
167.77
71.91
61.94
45.79
14.06
00 190 180 170 160 150 140 130 120 110 100 90 80 70 60 50 40 30 20 10 0
f1 (ppm)

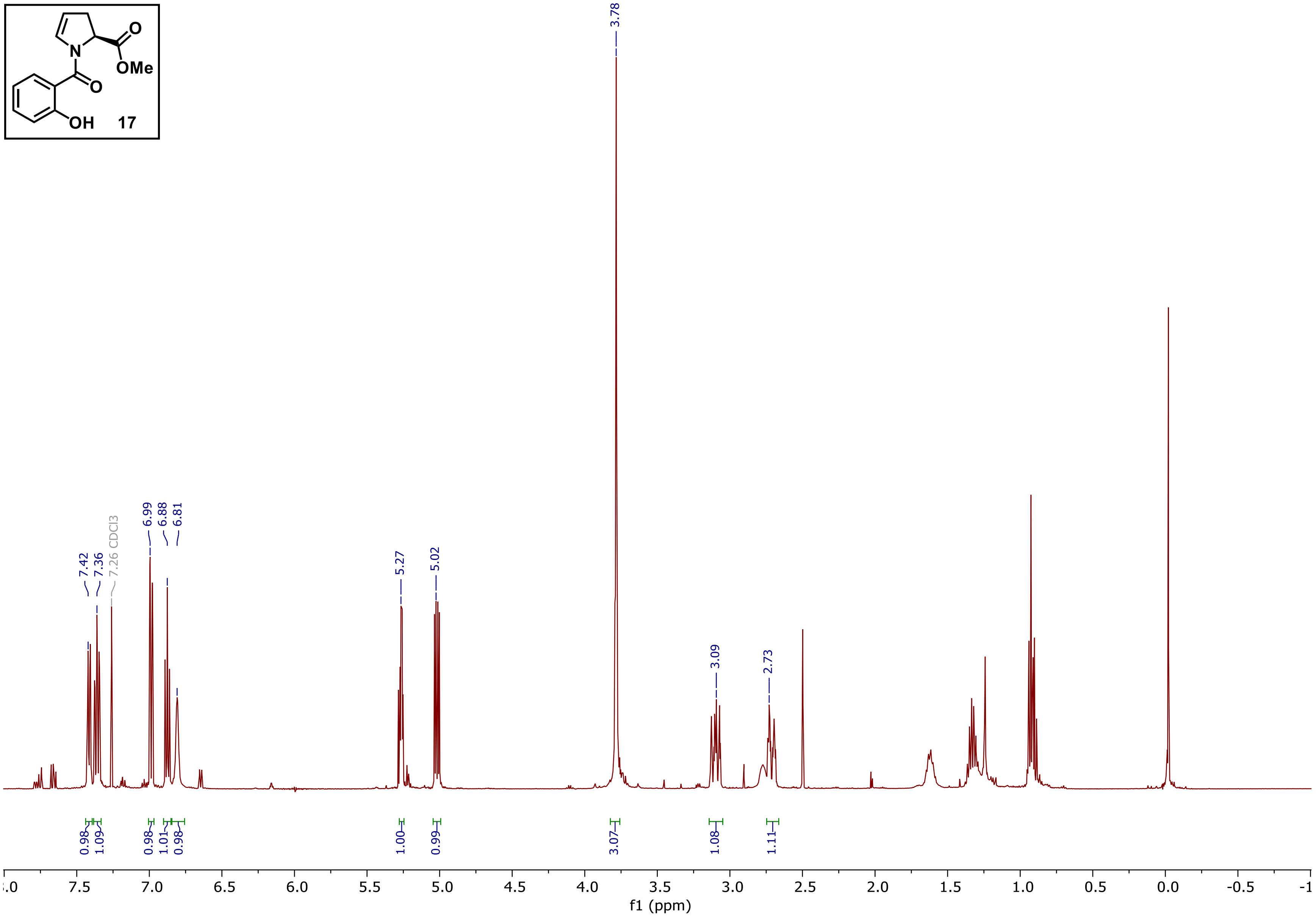

N
O
OMe
O
OH
17
7.42
7.36
7.26 CDCl3
6.99
6.88
6.81
5.27
5.02
3.78
3.09
2.73
0.98
1.09
0.98
1.01
0.98
1.00
0.99
3.07
1.08
1.11
7.5
7.0
6.5
6.0
5.5
5.0
4.5
4.0
3.5
3.0
2.5
2.0
1.5
1.0
0.5
0.0
-0.5
f1 (ppm)

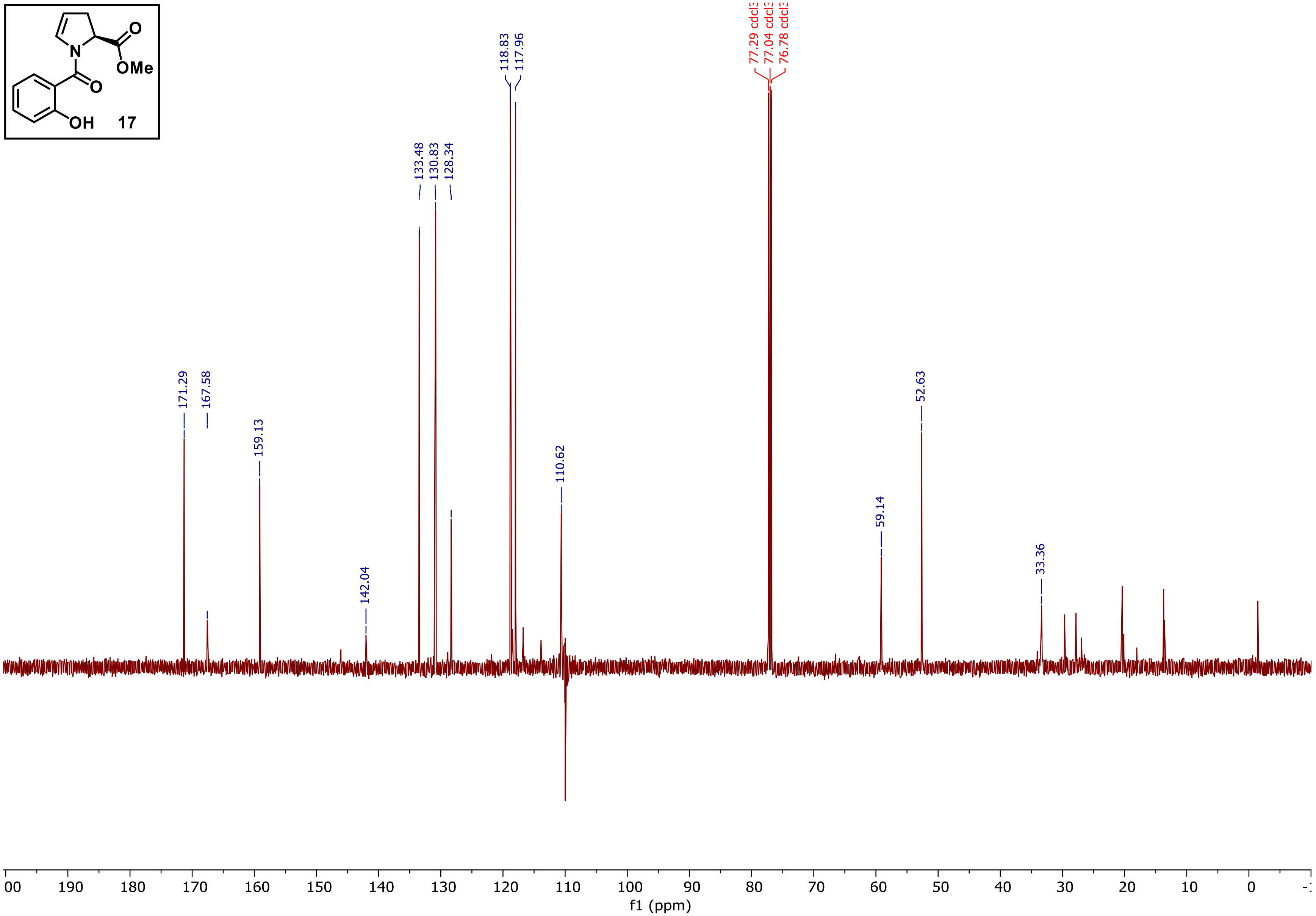
N
O
OMe
O
OH
17
171.29
167.58
159.13
142.04
133.48
130.83
128.34
118.83
117.96
110.62
77.29 cdcl3
77.04 cdcl3
76.78 cdcl3
59.14
52.63
33.36
f1 (ppm)

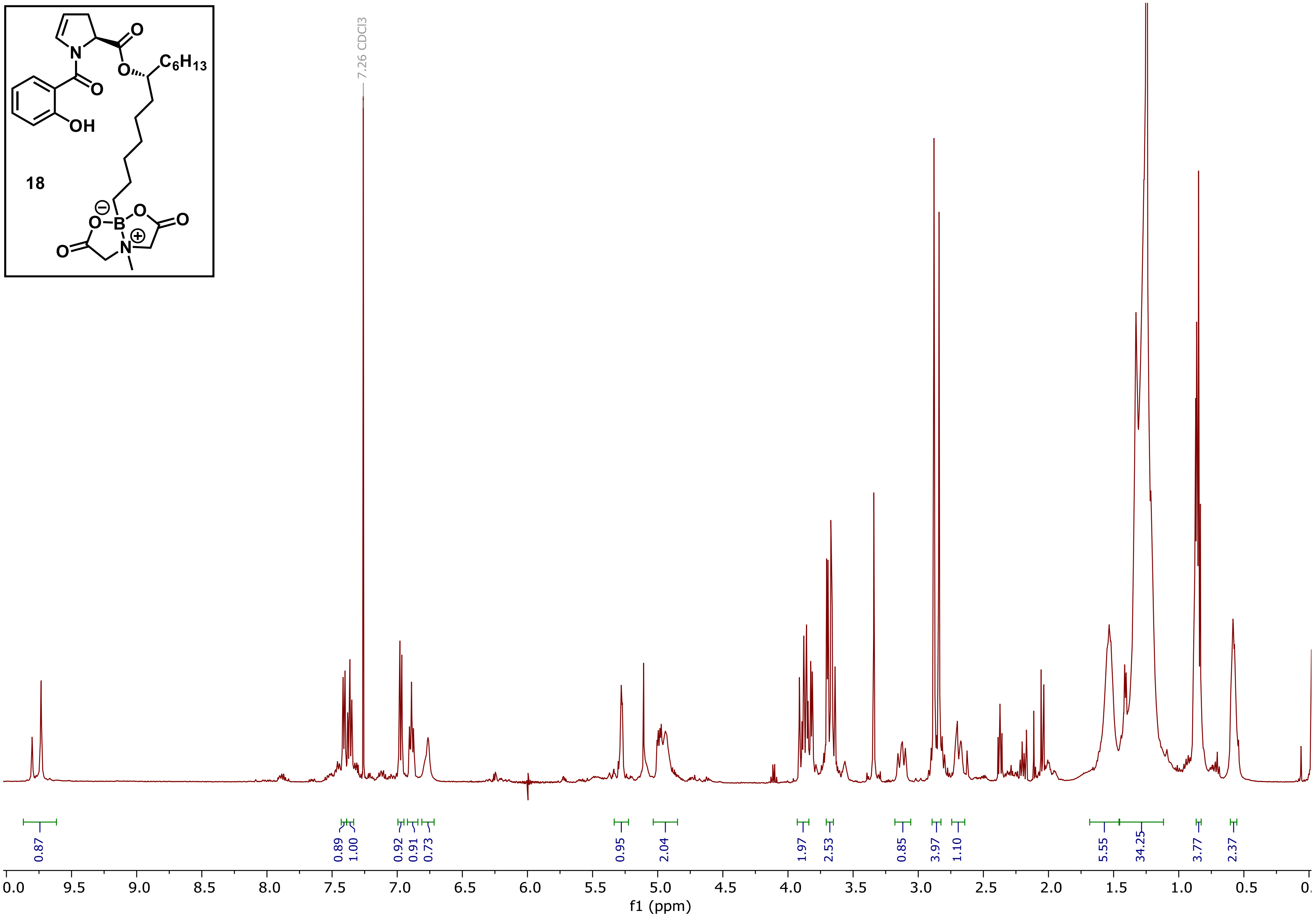

18
7.26 CDCl3
0.87
0.89
1.00
0.92
0.91
0.73
0.95
2.04
1.97
2.53
0.85
3.97
1.10
5.55
34.25
3.77
2.37
f1 (ppm)

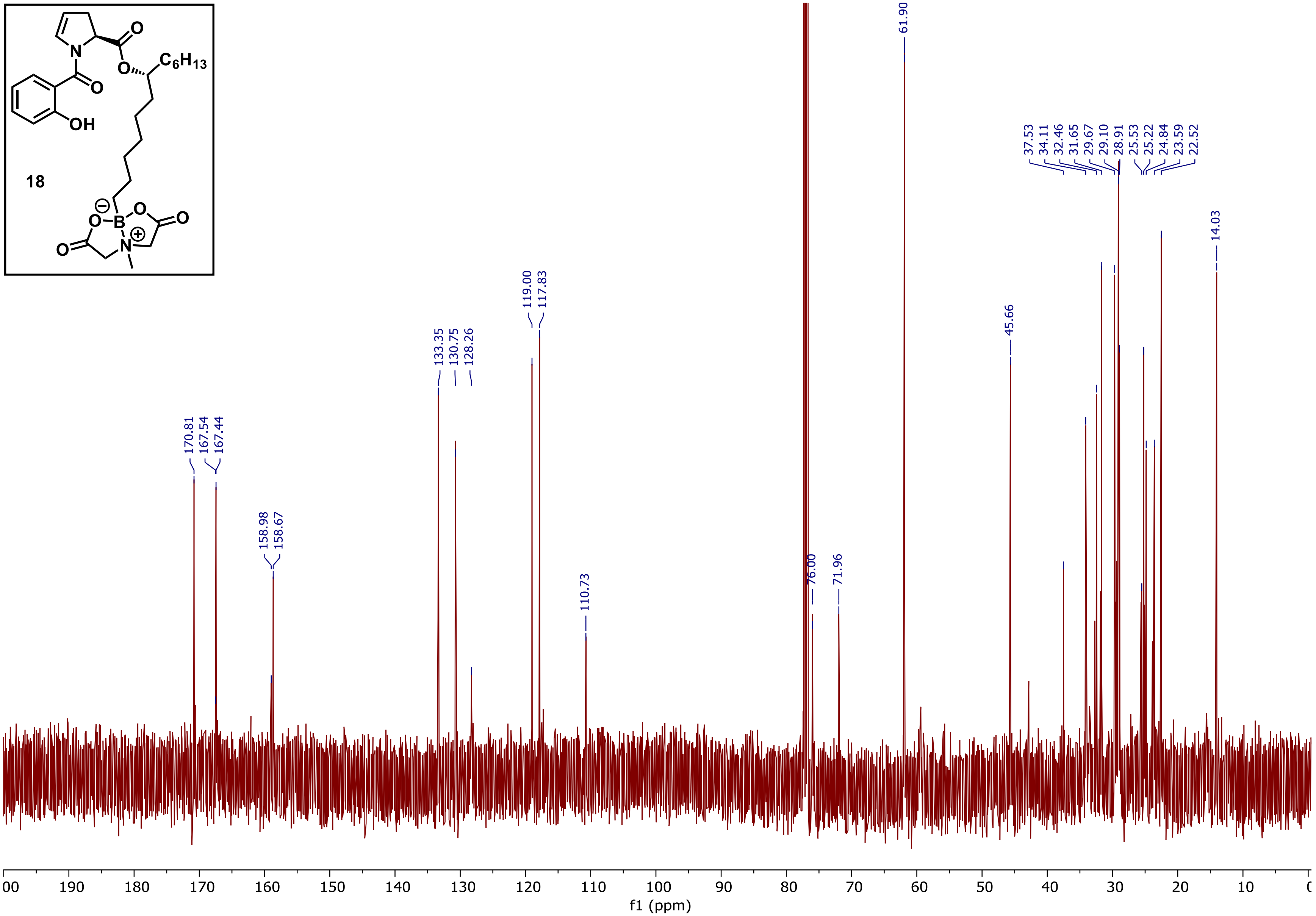

18
OH
O
N
O
C6H13
O
B
N
170.81
167.54
167.44
158.98
158.67
133.35
130.75
128.26
119.00
117.83
110.73
76.00
71.96
61.90
45.66
37.53
34.11
32.46
31.65
29.67
29.10
28.91
25.53
25.22
24.84
23.59
22.52
14.03
f1 (ppm)

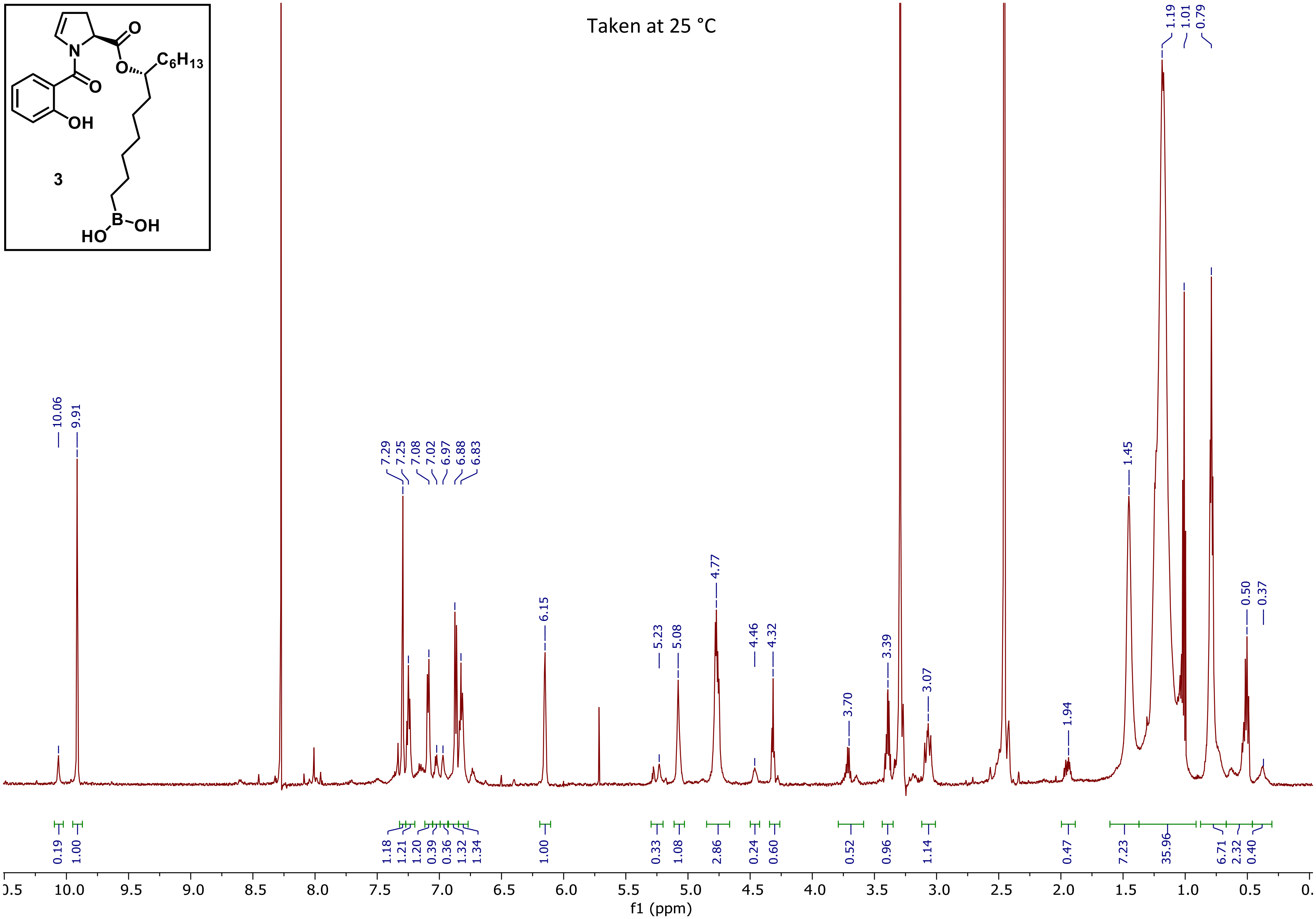
Taken at 25 °C
3
f1 (ppm)

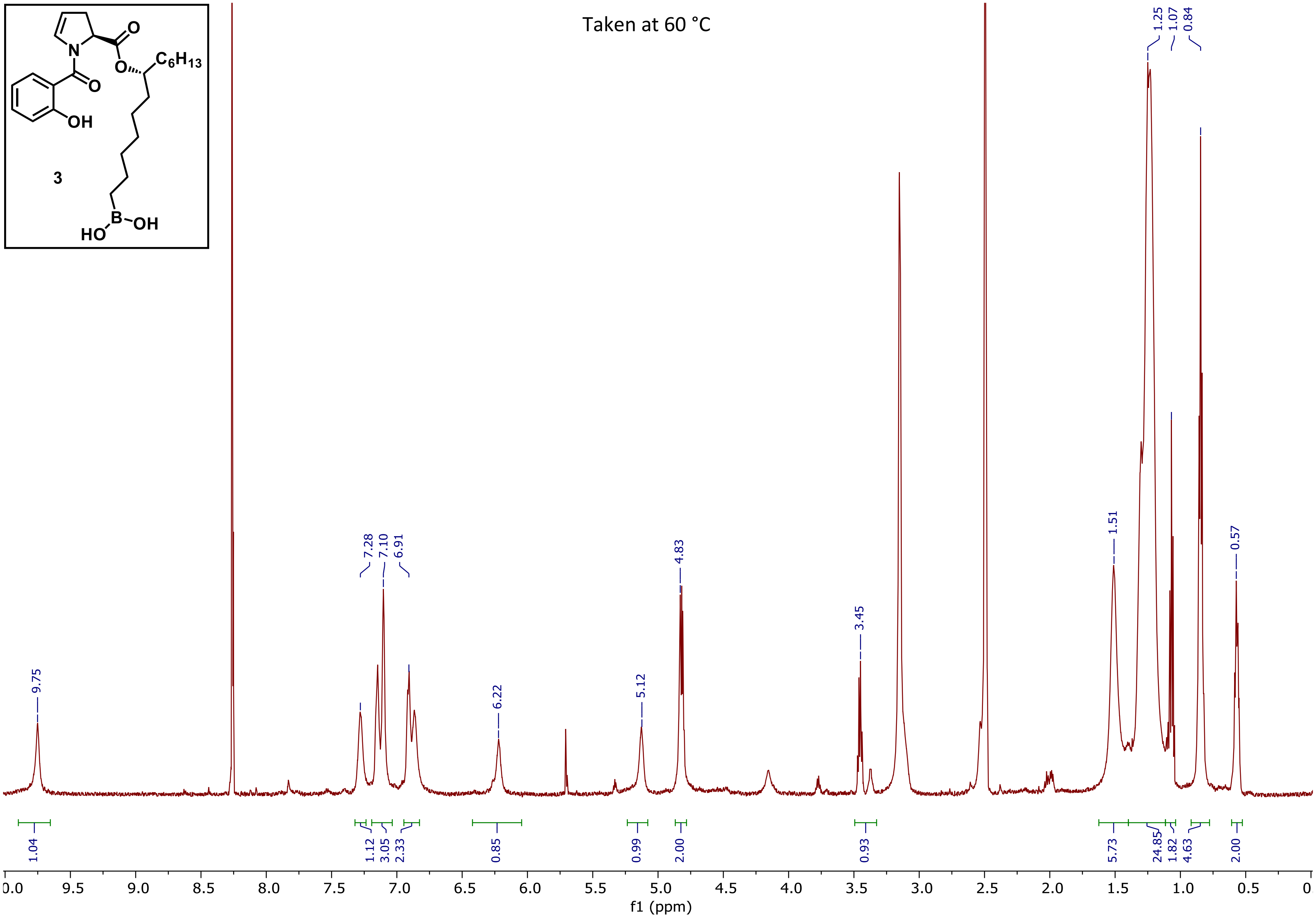

3
Taken at 60 °C
9.75
7.28
7.10
6.91
6.22
5.12
4.83
3.45
1.51
1.25
1.07
0.84
0.57
1.04
1.12
3.05
2.33
0.85
0.99
2.00
0.93
5.73
24.85
1.82
4.63
2.00
f1 (ppm)

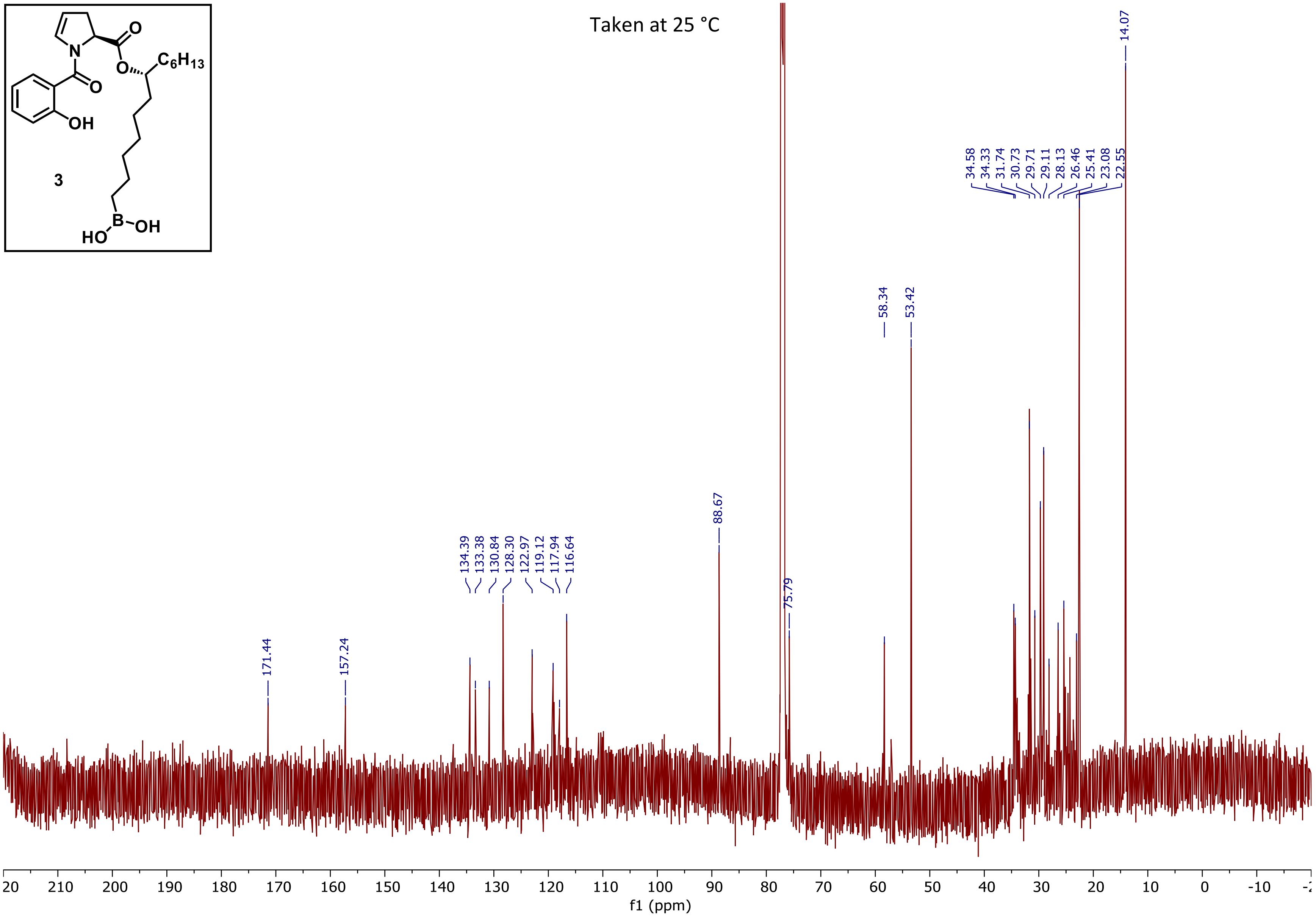
3
Taken at 25 °C
171.44
157.24
134.39
133.38
130.84
128.30
122.97
119.12
117.94
116.64
88.67
75.79
58.34
53.42
34.58
34.33
31.74
30.73
29.71
29.11
28.13
26.46
25.41
23.08
22.55
14.07
f1 (ppm)

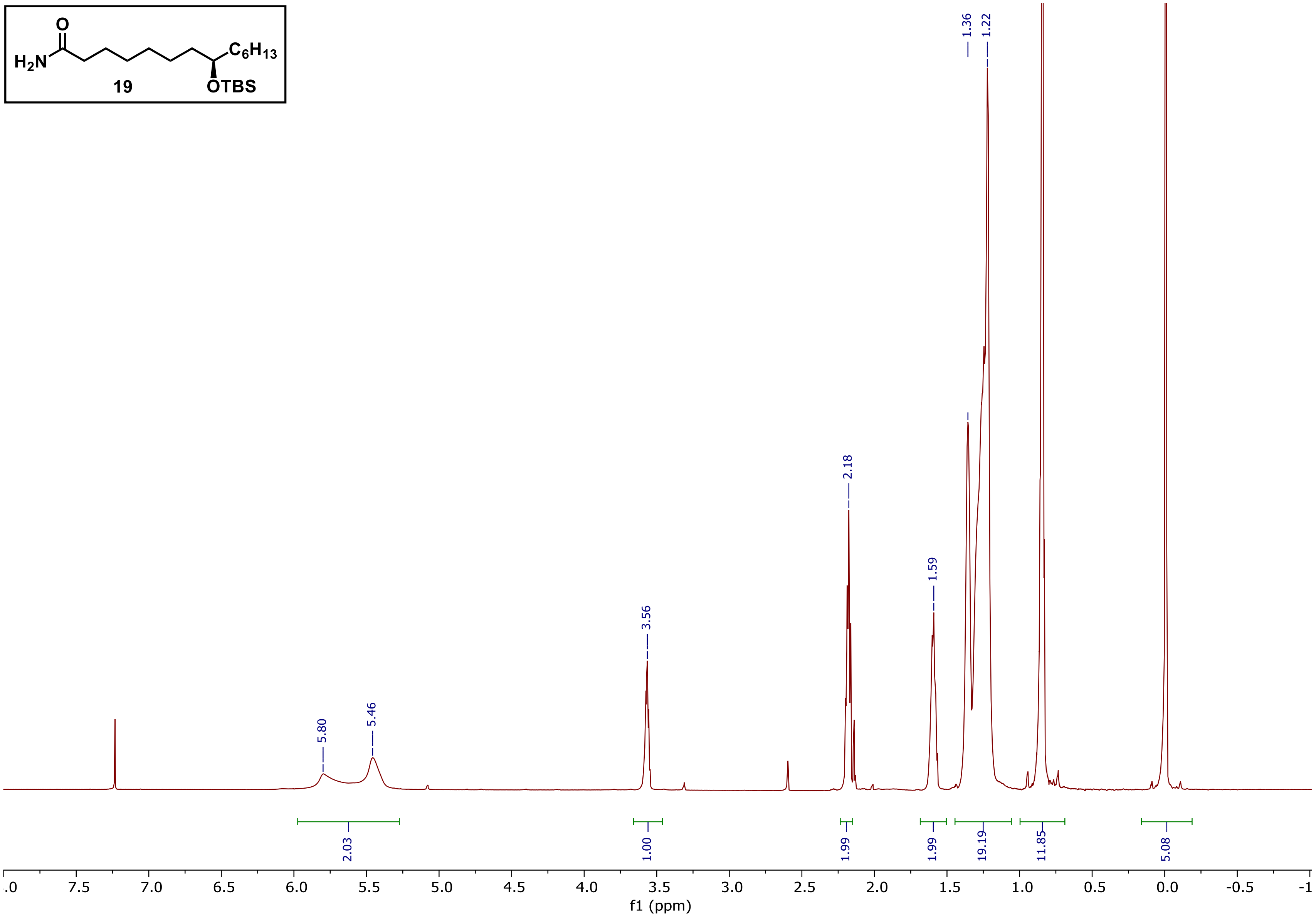

O
H2N
C6H13
19
OTBS
5.80
5.46
3.56
2.18
1.59
1.36
1.22
2.03
1.00
1.99
1.99
19.19
11.85
5.08
.0
7.5
7.0
6.5
6.0
5.5
5.0
4.5
4.0
3.5
3.0
2.5
2.0
1.5
1.0
0.5
0.0
-0.5
-1
f1 (ppm)

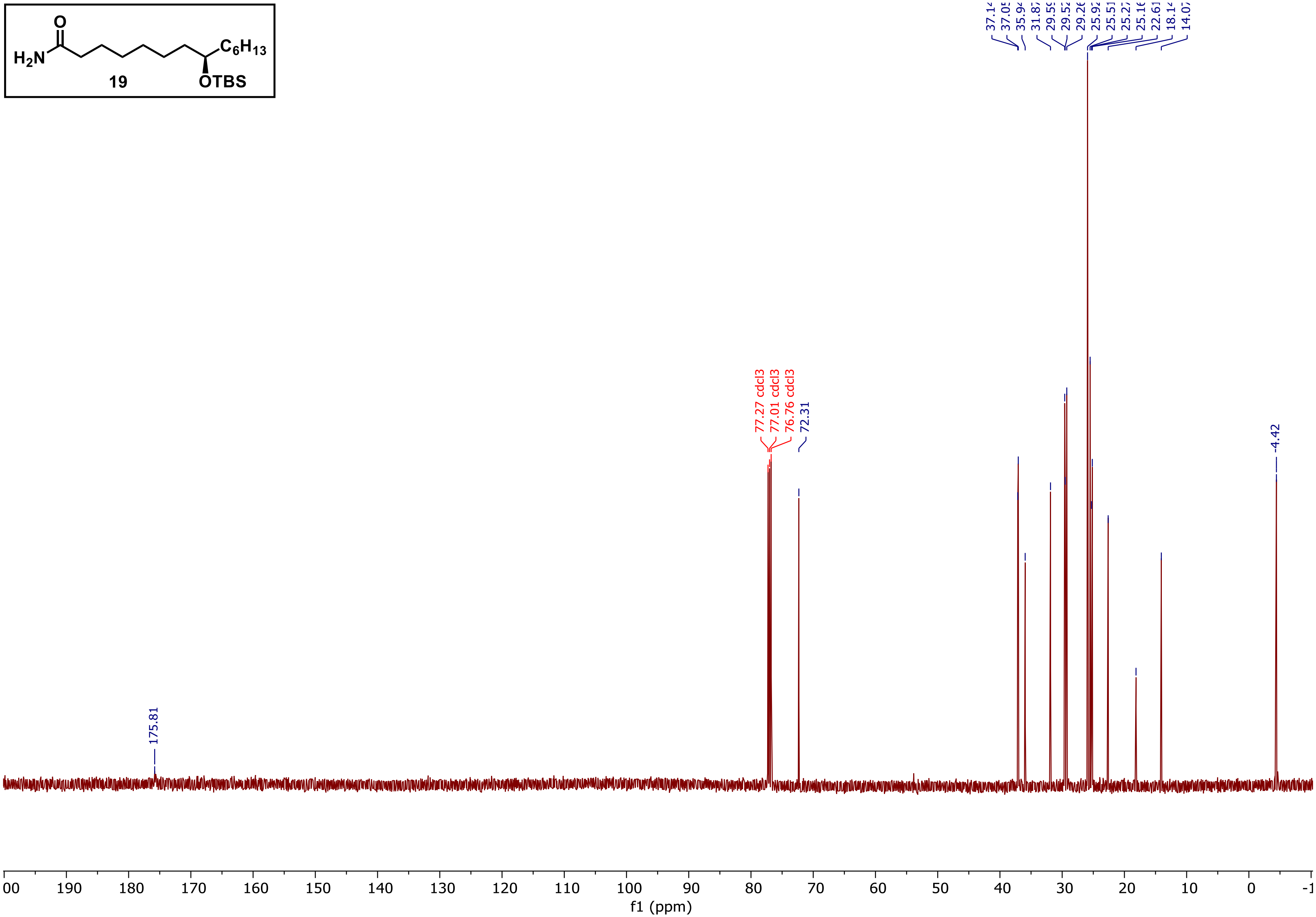
H2N
O
C6H13
19
OTBS
37.1
37.05
35.9
31.8
29.5
29.52
29.2
25.92
25.51
25.2
25.1
22.61
18.1
14.0
77.27 cdcl3
77.01 cdcl3
76.76 cdcl3
72.31
175.81
-4.42
f1 (ppm)

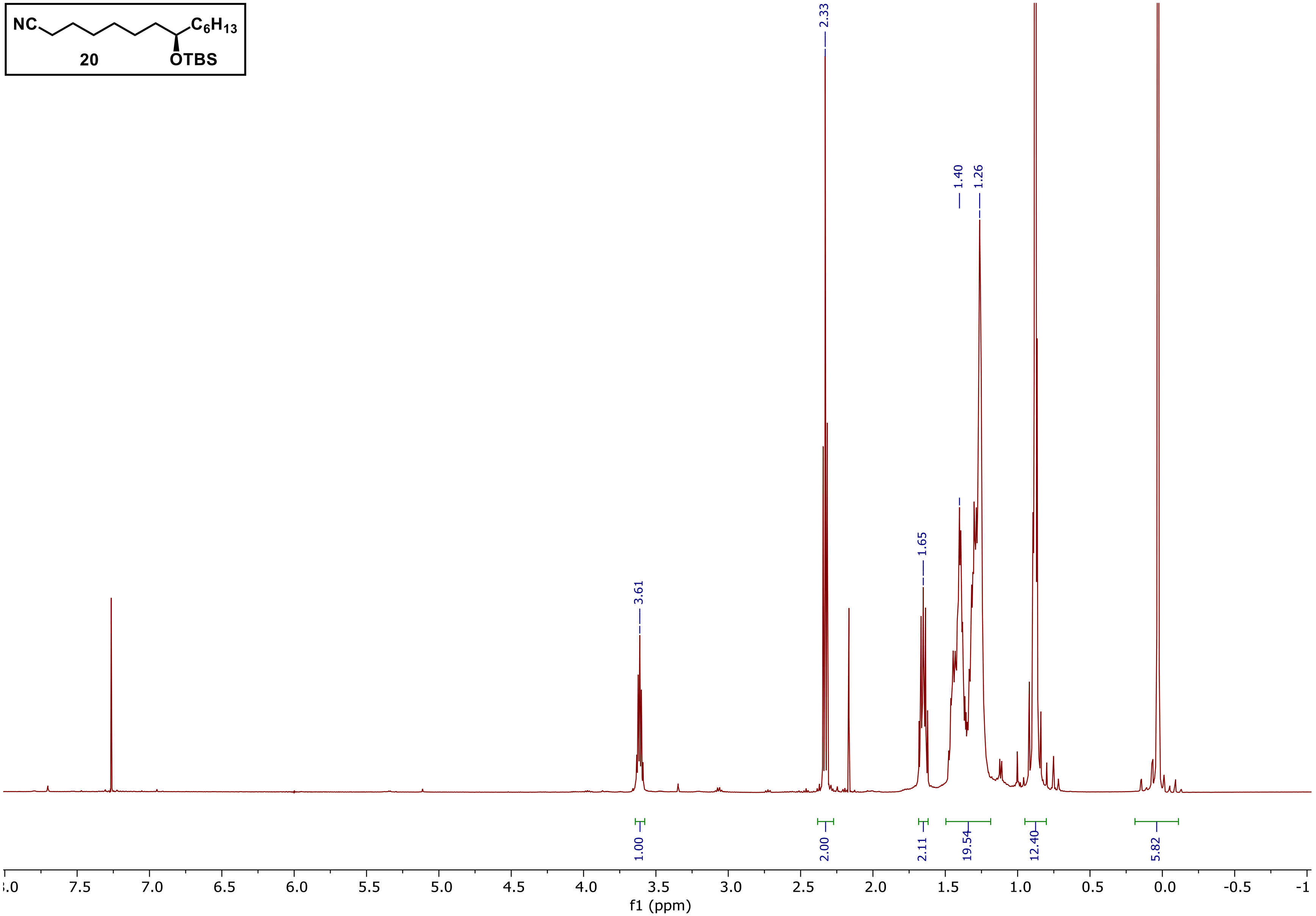
NC
C6H13
20
OTBS
2.33
1.40
1.26
1.65
3.61
1.00
2.00
2.11
19.54
12.40
5.82
f1 (ppm)

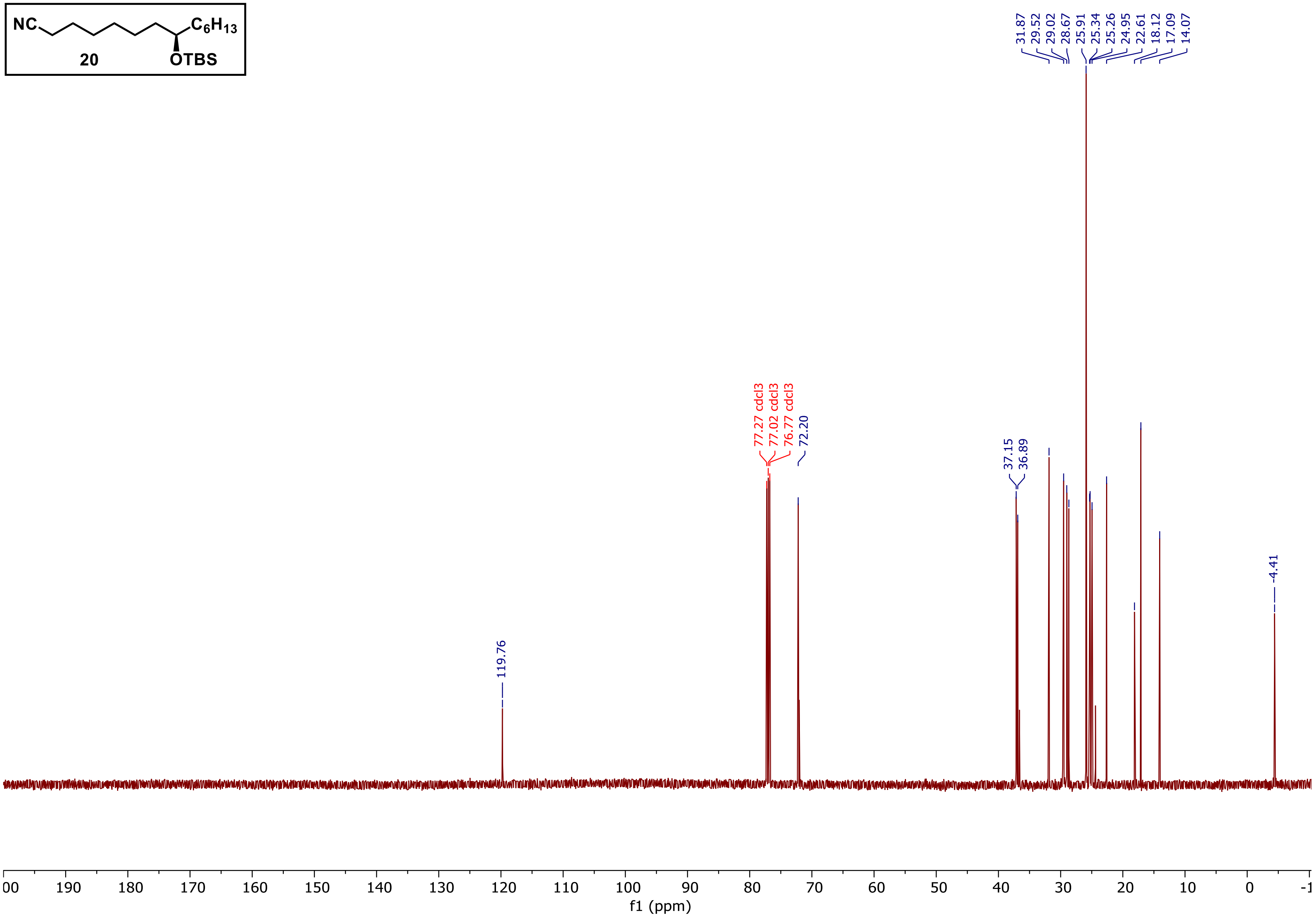
NC
C6H13
20
OTBS
31.87
29.52
29.02
28.67
25.91
25.34
25.26
24.95
22.61
18.12
17.09
14.07
77.27 cdcl3
77.02 cdcl3
76.77 cdcl3
72.20
37.15
36.89
119.76
-4.41
f1 (ppm)

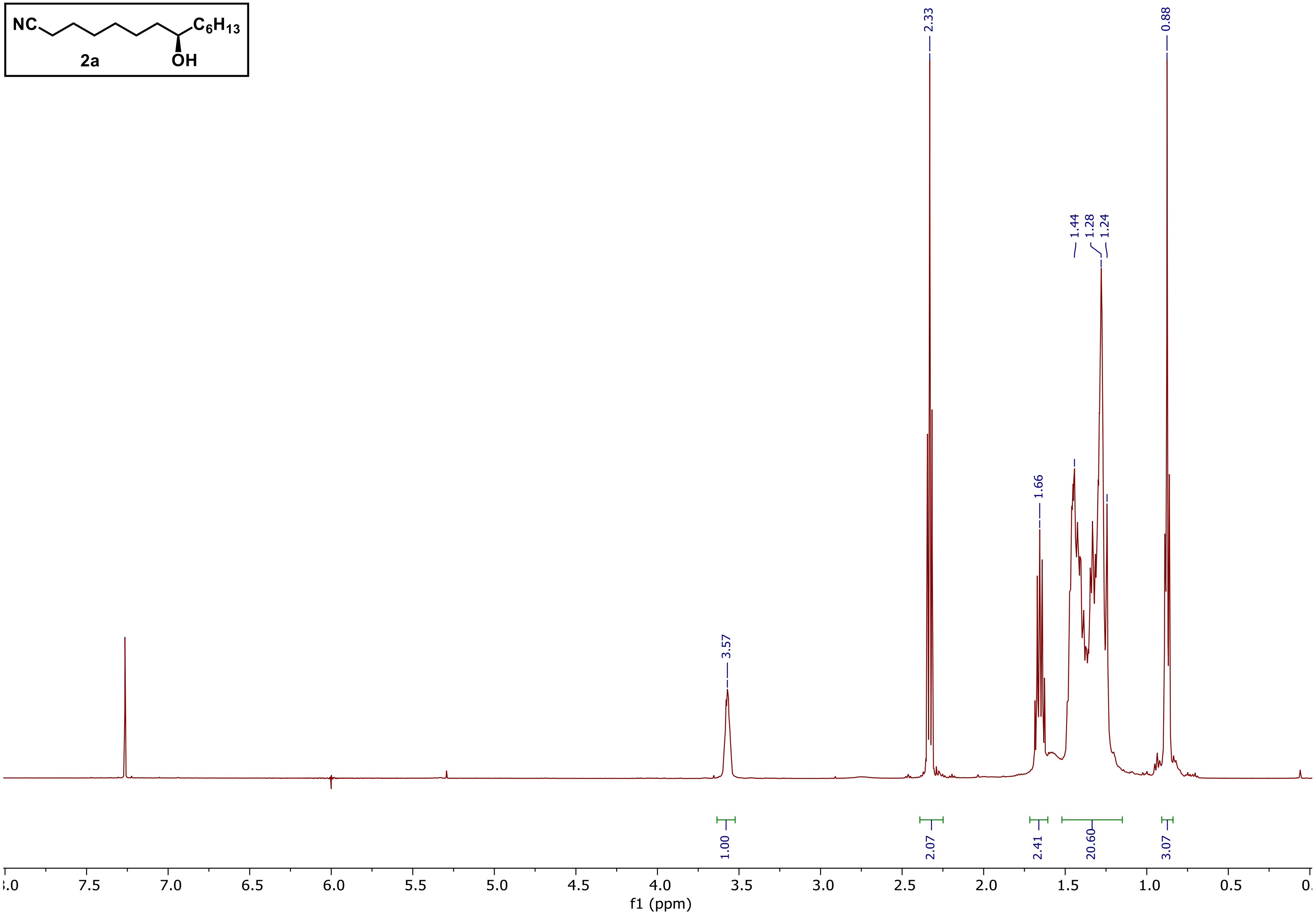
NC
C6H13
OH
2a
2.33
0.88
1.44
1.28
1.24
1.66
3.57
1.00
2.07
2.41
20.60
3.07
f1 (ppm)

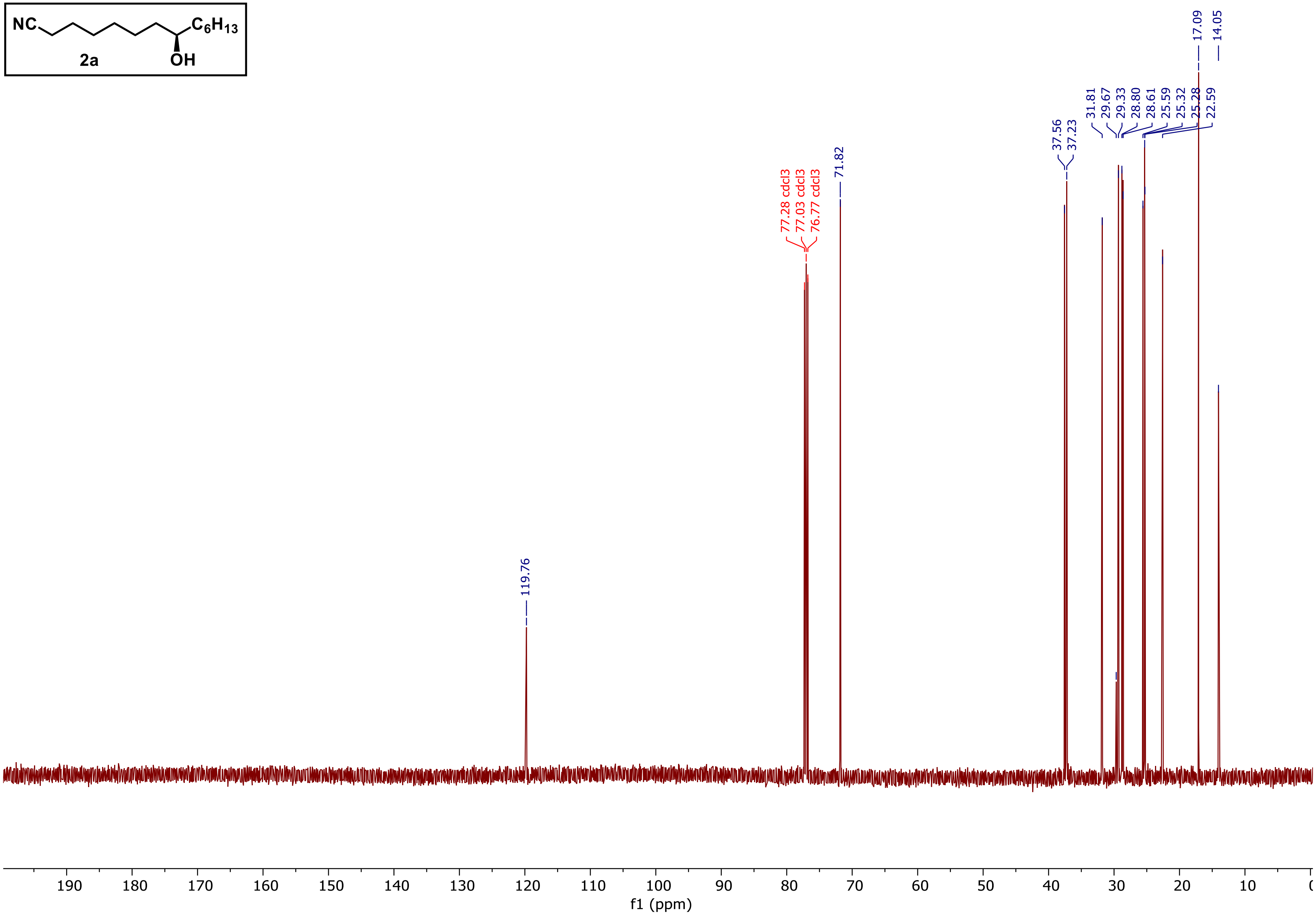
NC
C6H13
OH
2a
119.76
77.28 cdcl3
77.03 cdcl3
76.77 cdcl3
71.82
37.56
37.23
31.81
29.67
29.33
28.80
28.61
25.59
25.32
25.28
22.59
17.09
14.05
190 180 170 160 150 140 130 120 110 100 90 80 70 60 50 40 30 20 10
f1 (ppm)

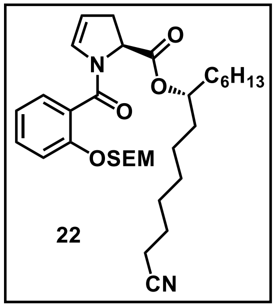

O
N
O
O
C6H13
OSEM
22
CN

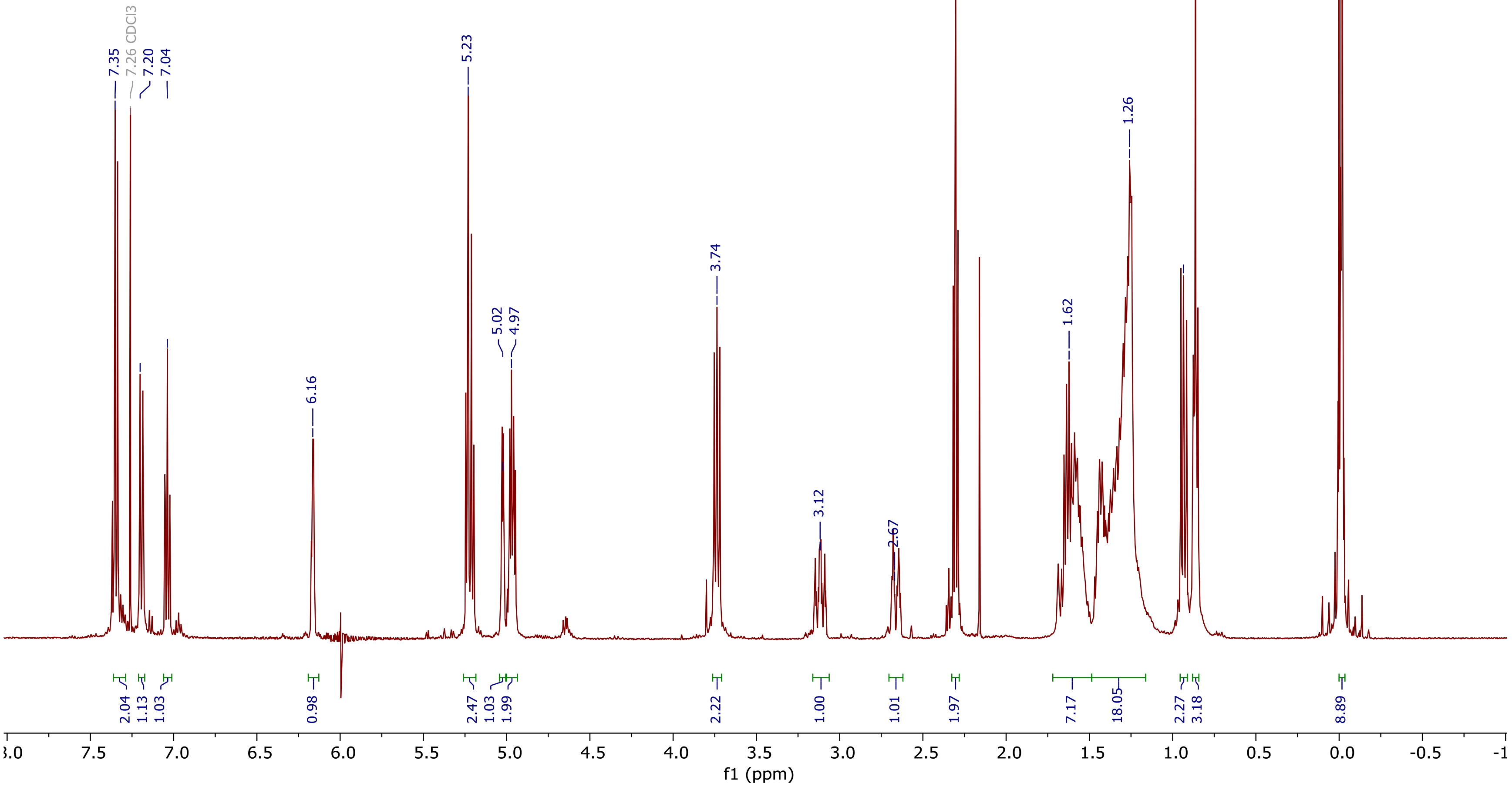

7.35
7.26 CDCl3
7.20
7.04
6.16
5.23
5.02
4.97
3.74
3.12
2.67
2.30
1.62
1.26
0.94
0.86
2.04
1.13
1.03
0.98
2.47
1.03
1.99
2.22
1.00
1.01
1.97
7.17
18.05
2.27
3.18
8.89
f1 (ppm)

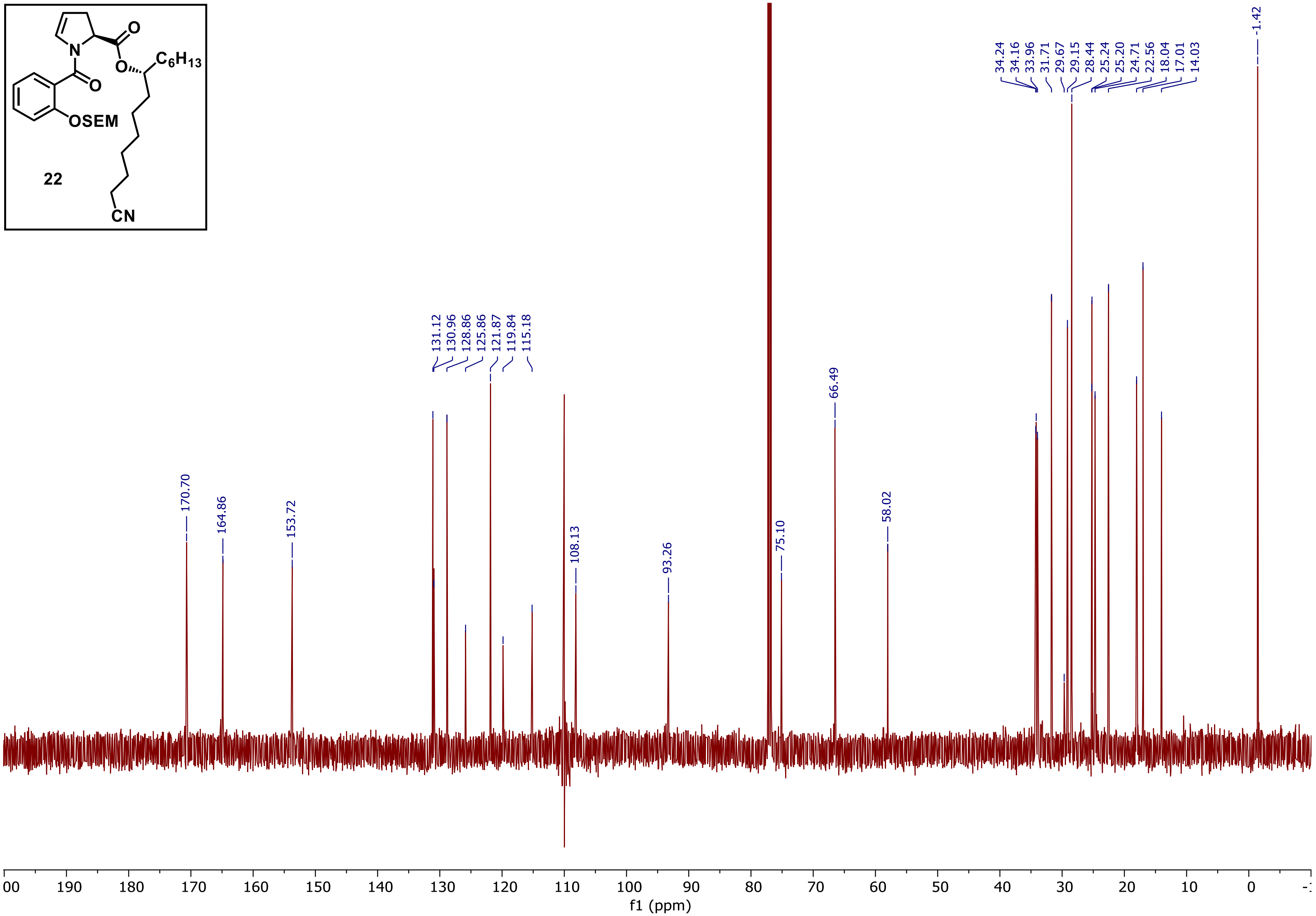

22
OSEM
CN
C6H13
170.70
164.86
153.72
131.12
130.96
128.86
125.86
121.87
119.84
115.18
108.13
93.26
75.10
66.49
58.02
34.24
34.16
33.96
31.71
29.67
29.15
28.44
25.24
25.20
24.71
22.56
18.04
17.01
14.03
-1.42
f1 (ppm)

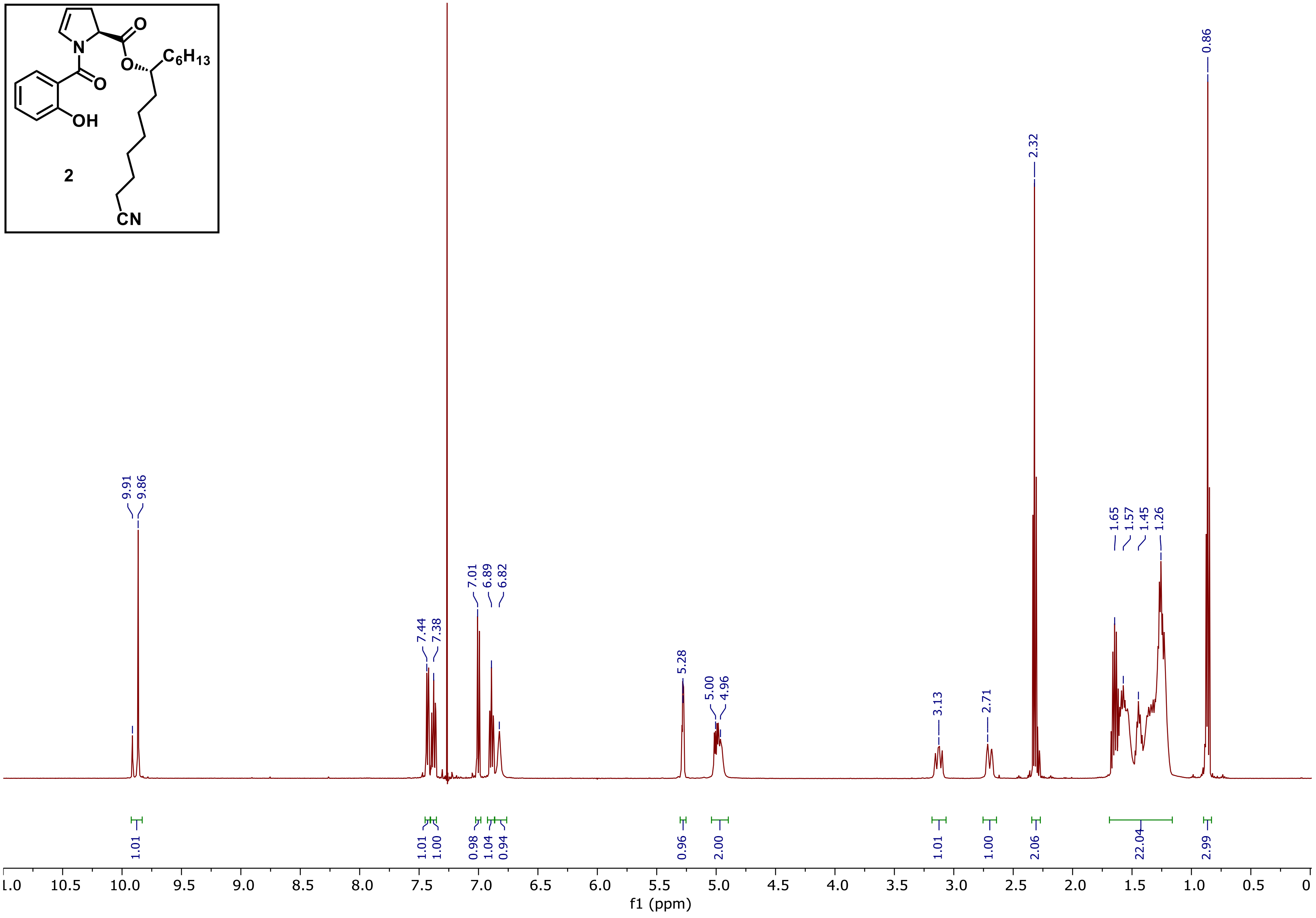
2
OH
CN
C_6H_{13}
9.91
9.86
7.44
7.38
7.01
6.89
6.82
5.28
5.00
4.96
3.13
2.71
2.32
1.65
1.57
1.45
1.26
0.86
1.01
1.01
1.00
0.98
1.04
0.94
0.96
2.00
1.01
1.00
2.06
22.04
2.99
f1 (ppm)

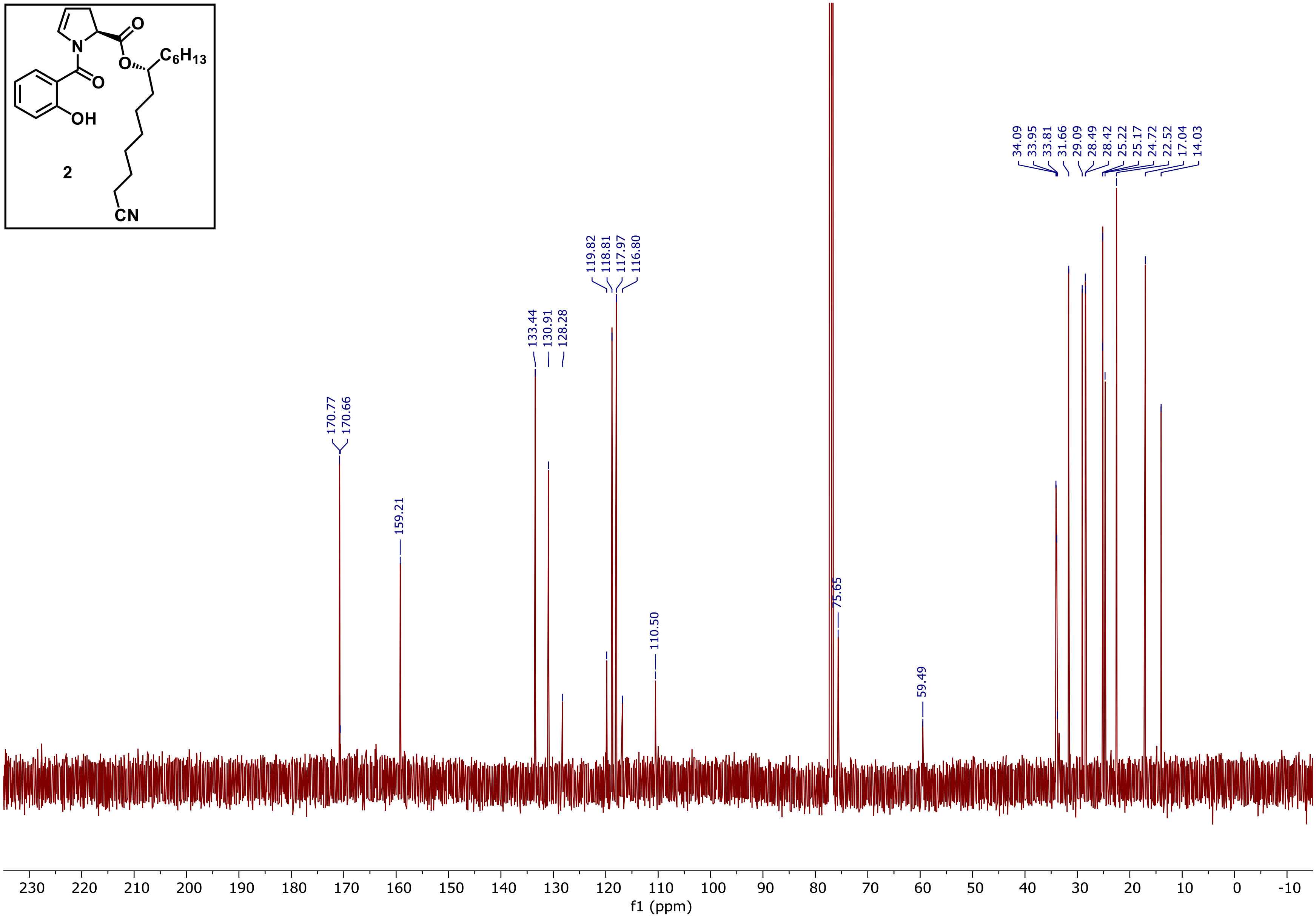

O
N
O
C_6H_{13}
O
OH
CN
2
170.77
170.66
159.21
133.44
130.91
128.28
119.82
118.81
117.97
116.80
110.50
75.65
59.49
34.09
33.95
33.81
31.66
29.09
28.49
28.42
25.22
25.17
24.72
22.52
17.04
14.03
230 220 210 200 190 180 170 160 150 140 130 120 110 100 90 80 70 60 50 40 30 20 10 0 -10
f1 (ppm)

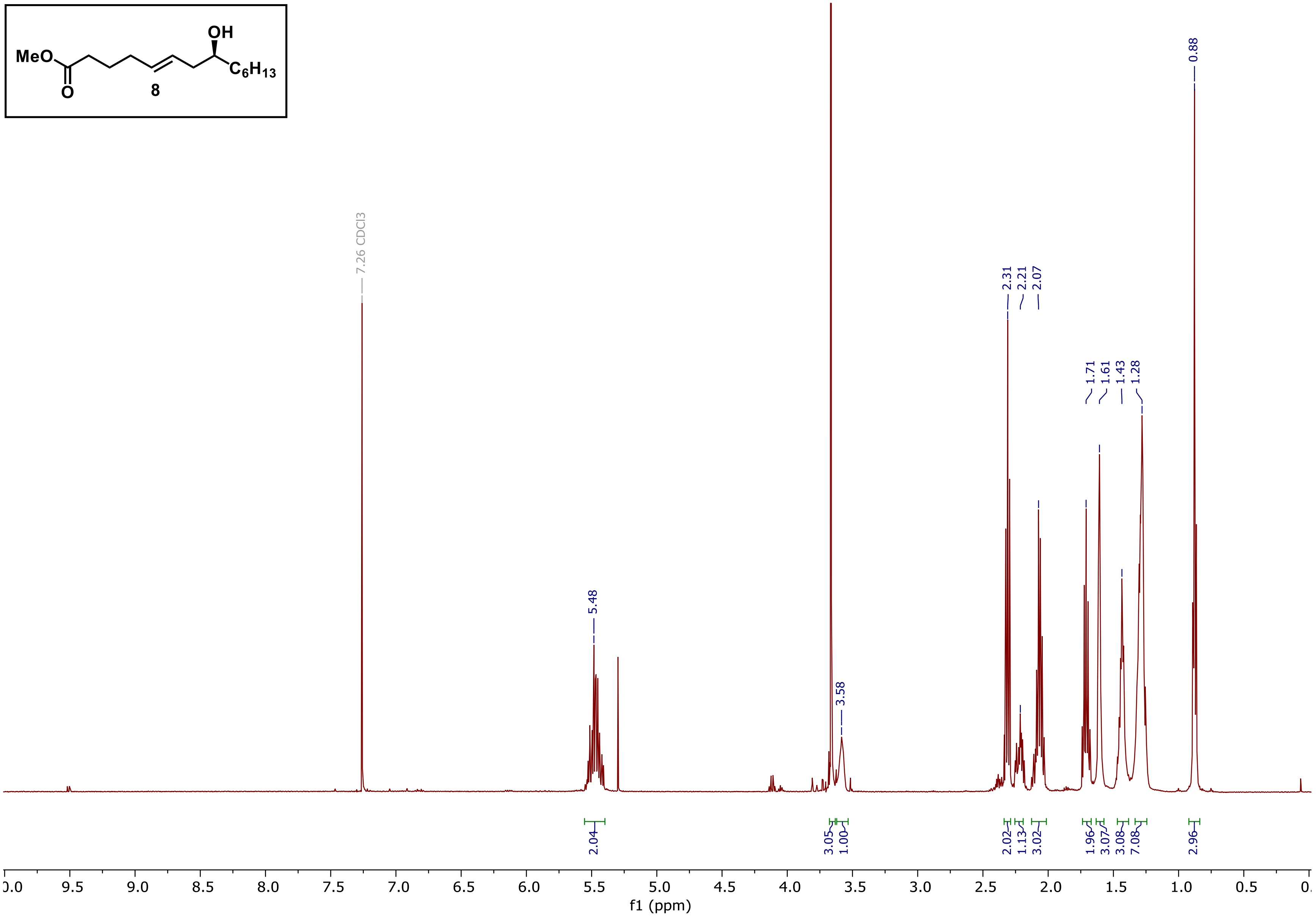
MeO
OH
O
8
C6H13
7.26 CDCl3
5.48
3.58
2.31
2.21
2.07
1.71
1.61
1.43
1.28
0.88
2.04
3.05
1.00
2.02
1.13
3.02
1.96
3.07
3.08
7.08
2.96
f1 (ppm)

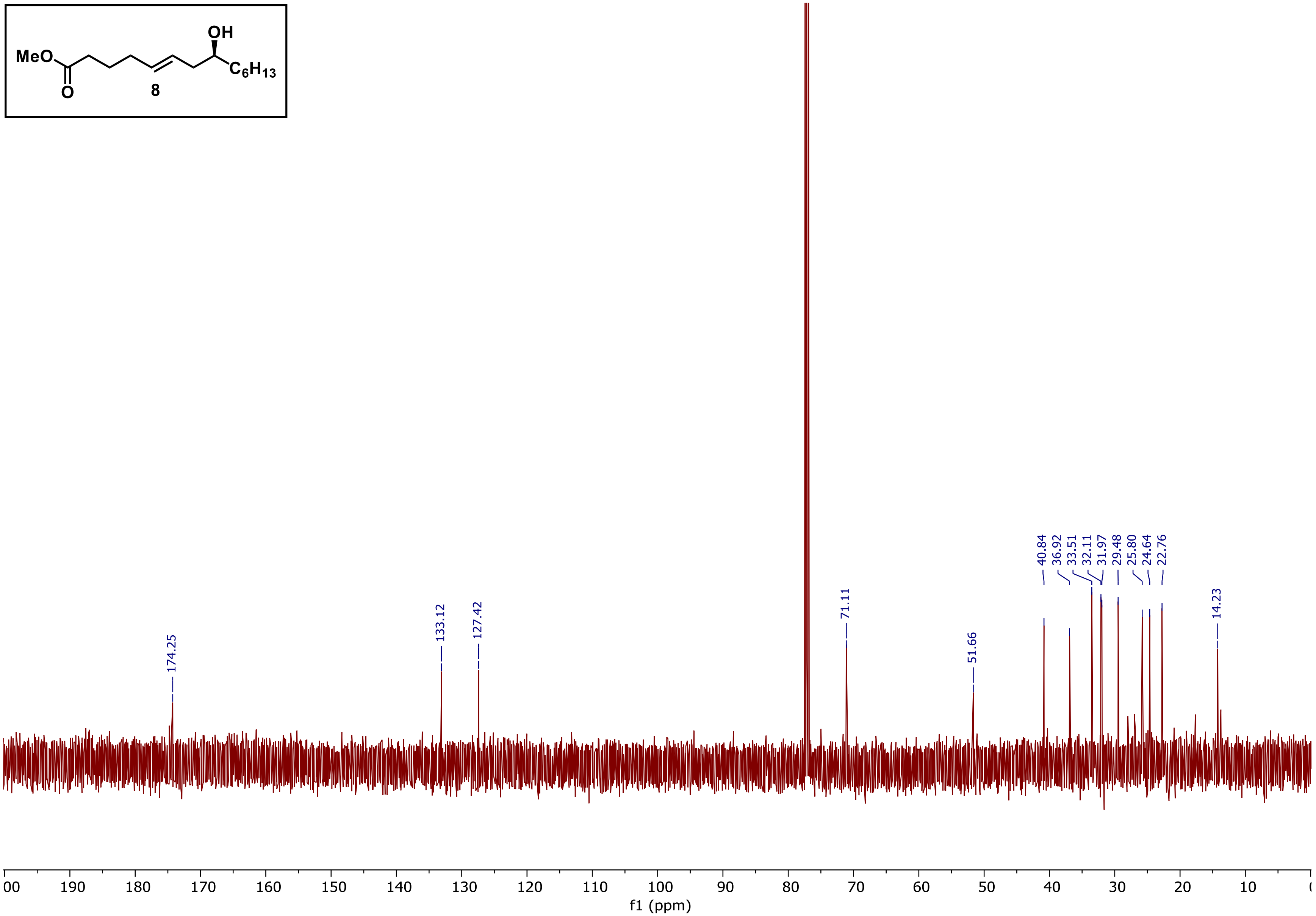

OH
MeO
O
C6H13
8
174.25
133.12
127.42
71.11
51.66
40.84
36.92
33.51
32.11
31.97
29.48
25.80
24.64
22.76
14.23
f1 (ppm)

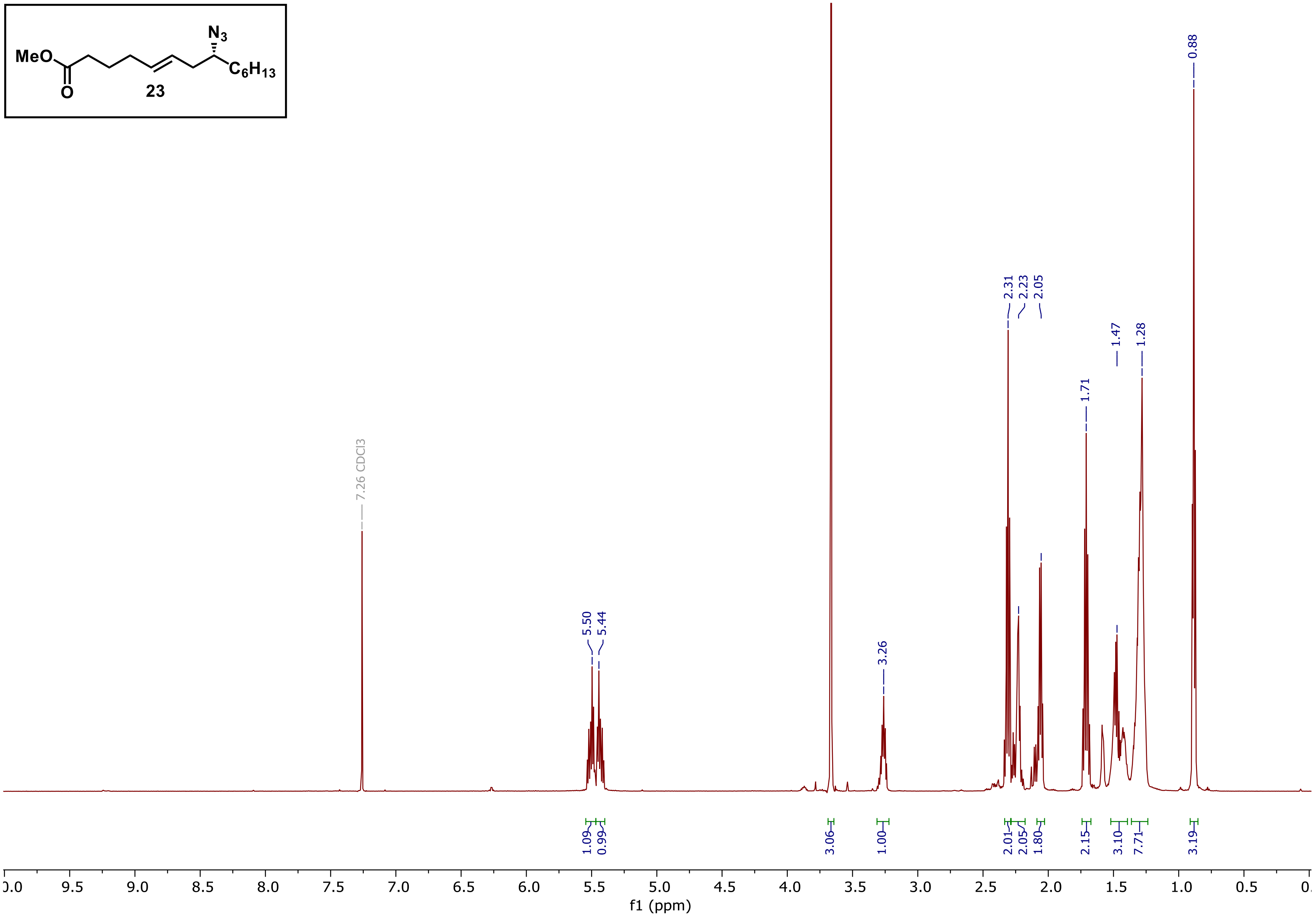
MeO
O
N3
C6H13
23
7.26 CDCl3
5.50
5.44
3.26
2.31
2.23
2.05
1.71
1.47
1.28
0.88
1.09
0.99
3.06
1.00
2.01
2.05
1.80
2.15
3.10
7.71
3.19
f1 (ppm)

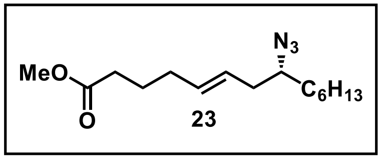
MeO
O
N3
C6H13
23

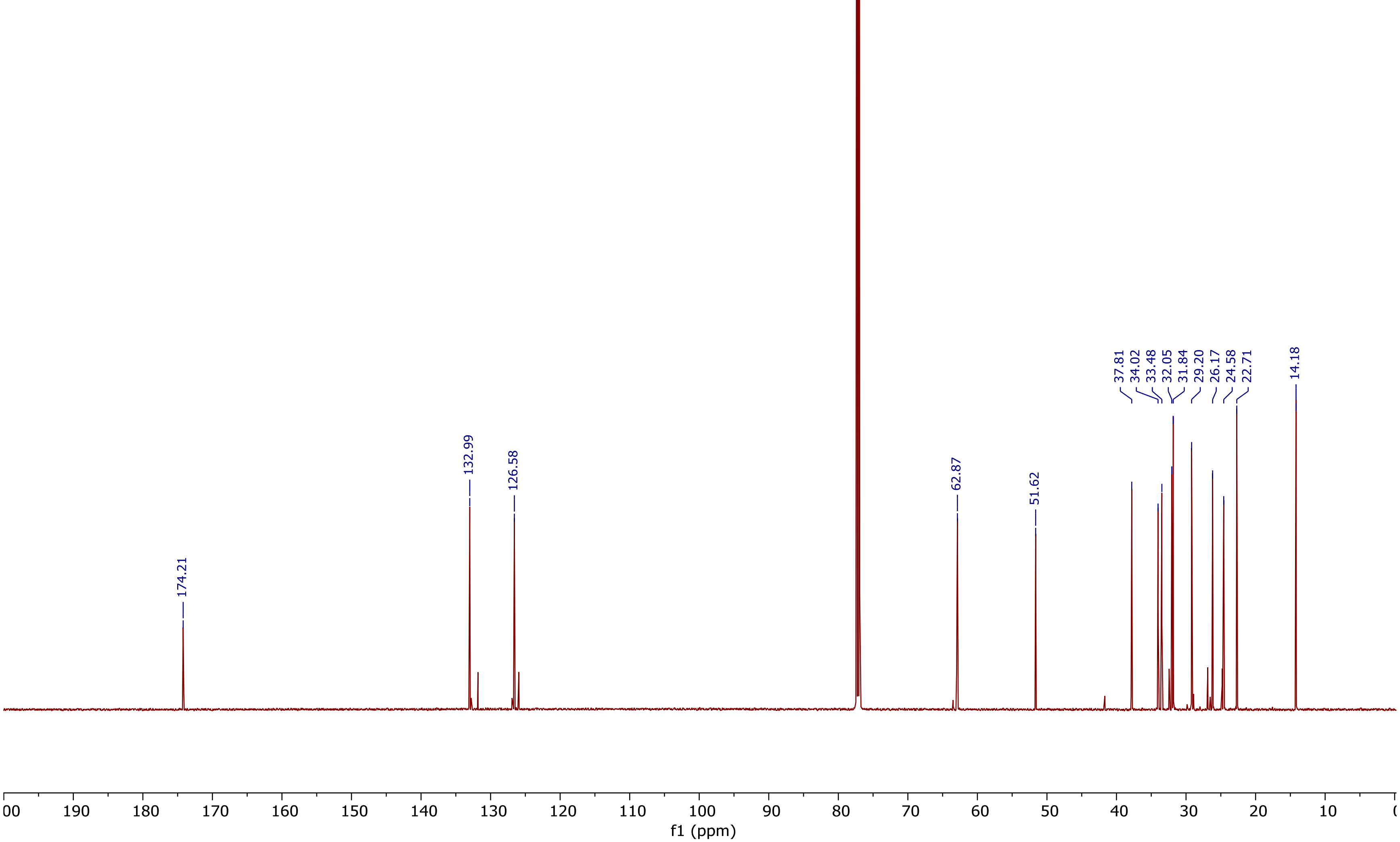
174.21
132.99
126.58
62.87
51.62
37.81
34.02
33.48
32.05
31.84
29.20
26.17
24.58
22.71
14.18
190 180 170 160 150 140 130 120 110 100 90 80 70 60 50 40 30 20 10
f1 (ppm)

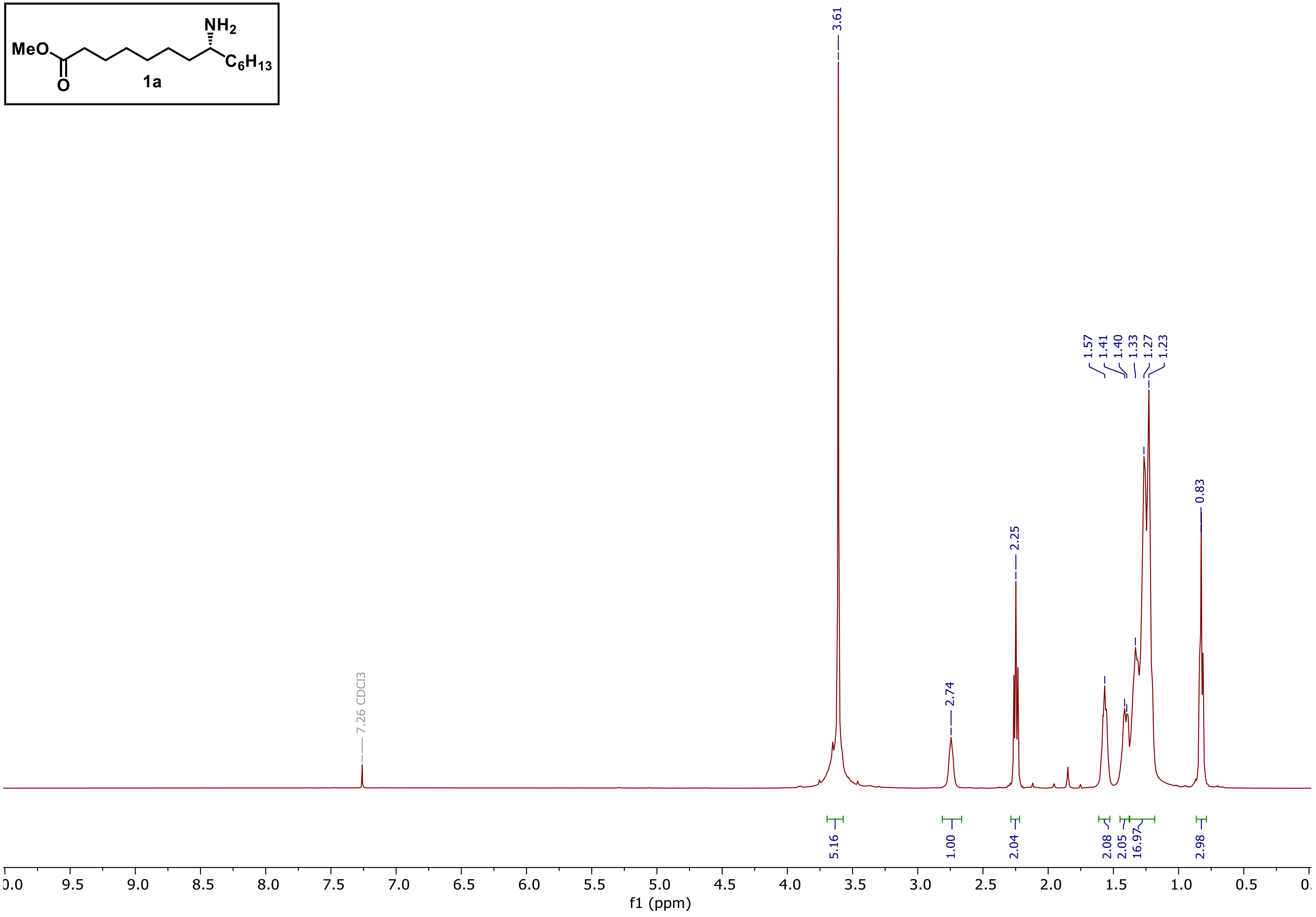
MeO
NH2
C6H13
O
1a
3.61
2.74
2.25
1.57
1.41
1.40
1.33
1.27
1.23
0.83
7.26 CDCl3
5.16
1.00
2.04
2.08
2.05
16.97
2.98
f1 (ppm)

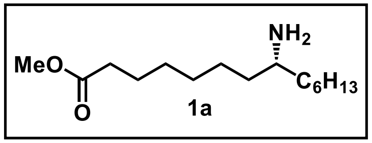
MeO
O
NH2
C6H13
1a

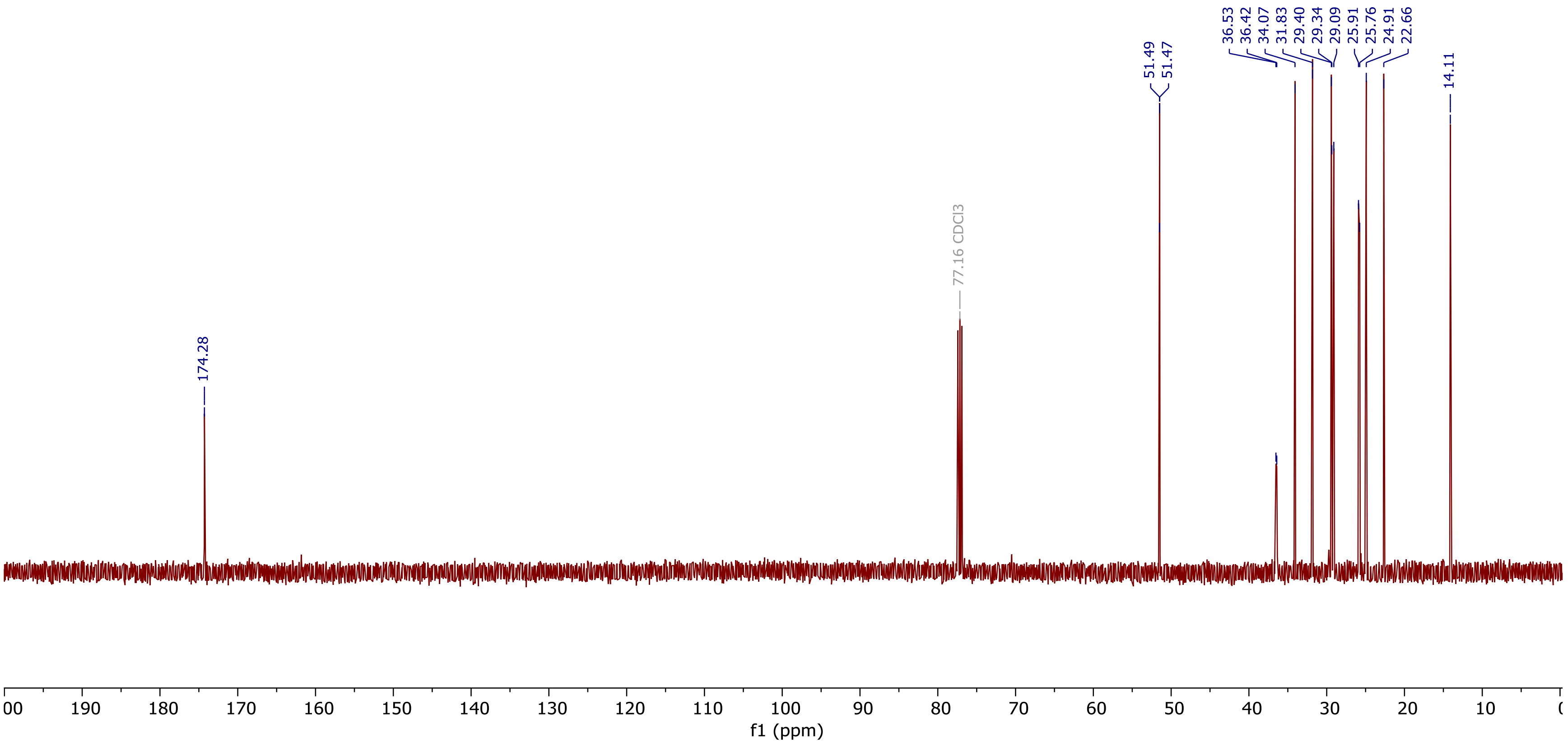
174.28
77.16 CDCl3
51.49
51.47
36.53
36.42
34.07
31.83
29.40
29.34
29.09
25.91
25.76
24.91
22.66
14.11
190
180
170
160
150
140
130
120
110
100
90
80
70
60
50
40
30
20
10
f1 (ppm)

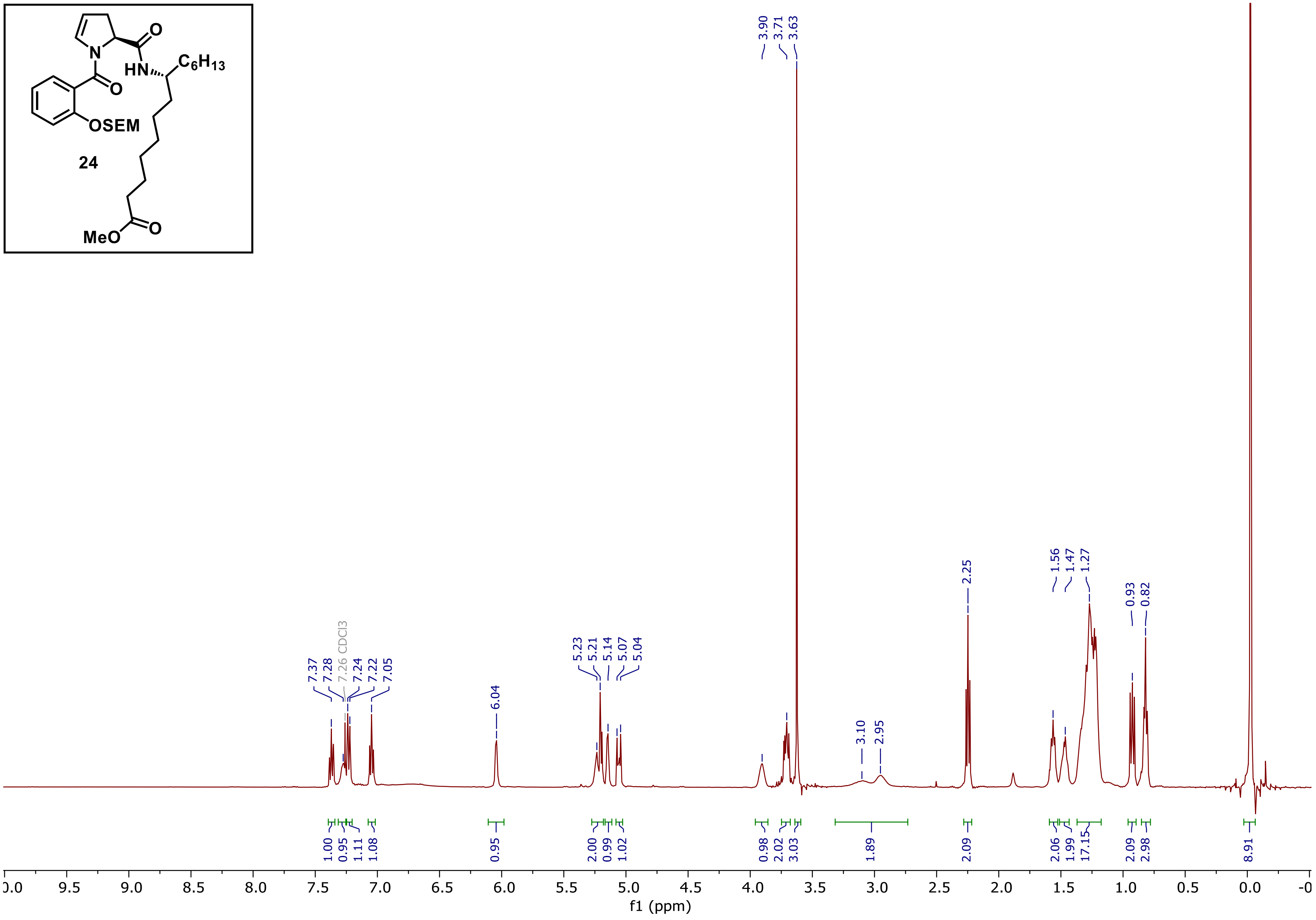

OSEM
24
HN
C6H13
MeO
7.37
7.28
7.26 CDCl3
7.24
7.22
7.05
6.04
5.23
5.21
5.14
5.07
5.04
3.90
3.71
3.63
3.10
2.95
2.25
1.56
1.47
1.27
0.93
0.82
1.00
0.95
1.11
1.08
0.95
2.00
0.99
1.02
0.98
2.02
3.03
1.89
2.09
2.06
1.99
17.15
2.09
2.98
8.91
f1 (ppm)

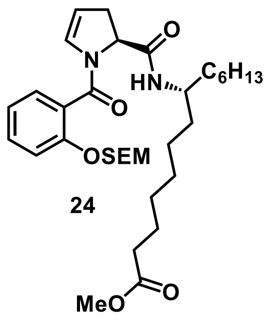

24

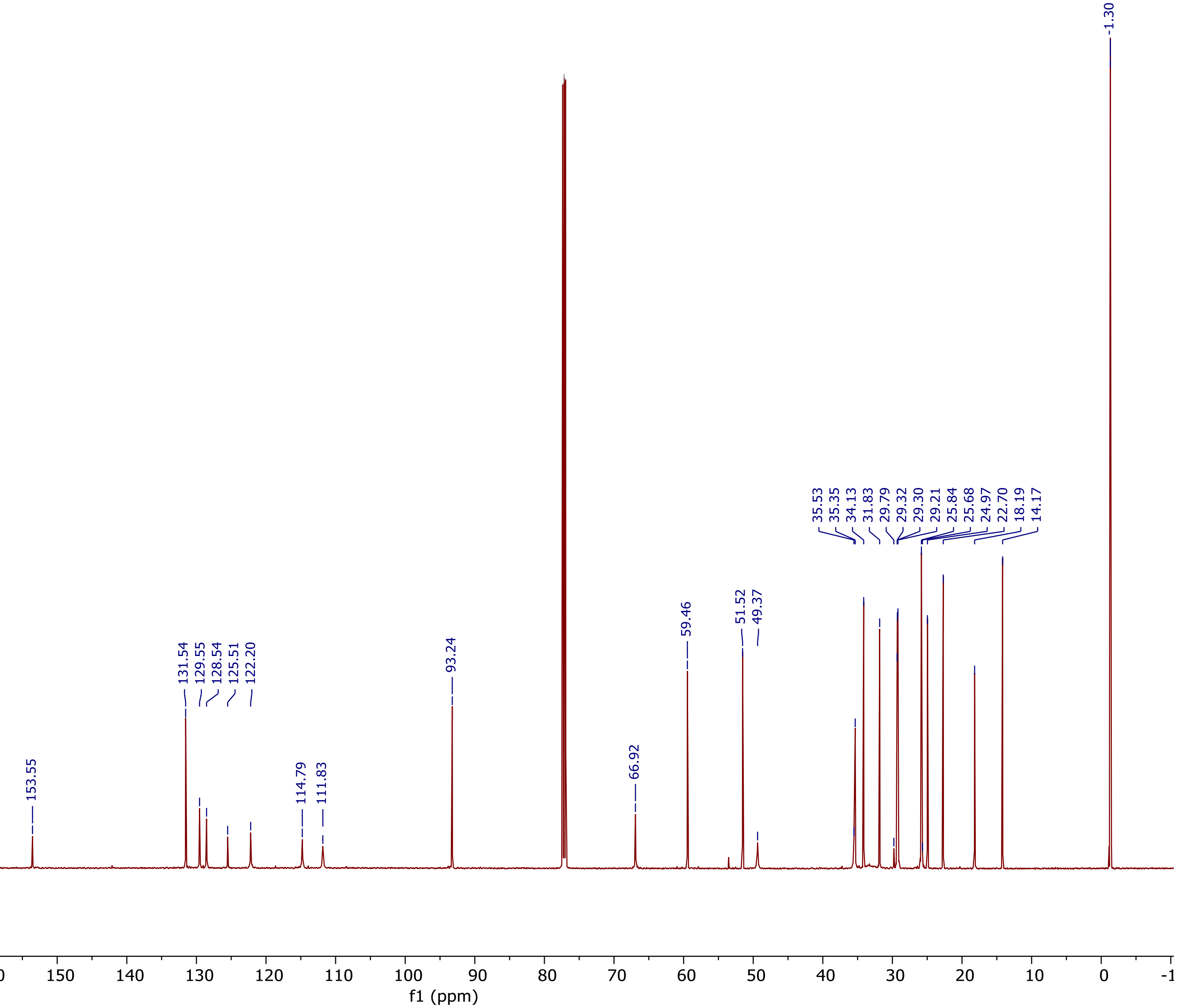

174.26
169.78
166.04
153.55
131.54
129.55
128.54
125.51
122.20
114.79
111.83
93.24
66.92
59.46
51.52
49.37
35.53
35.35
34.13
31.83
29.79
29.32
29.30
29.21
25.84
25.68
24.97
22.70
18.19
14.17
-1.30
00
190
180
170
160
150
140
130
120
110
100
90
80
70
60
50
40
30
20
10
0
-1
f1 (ppm)

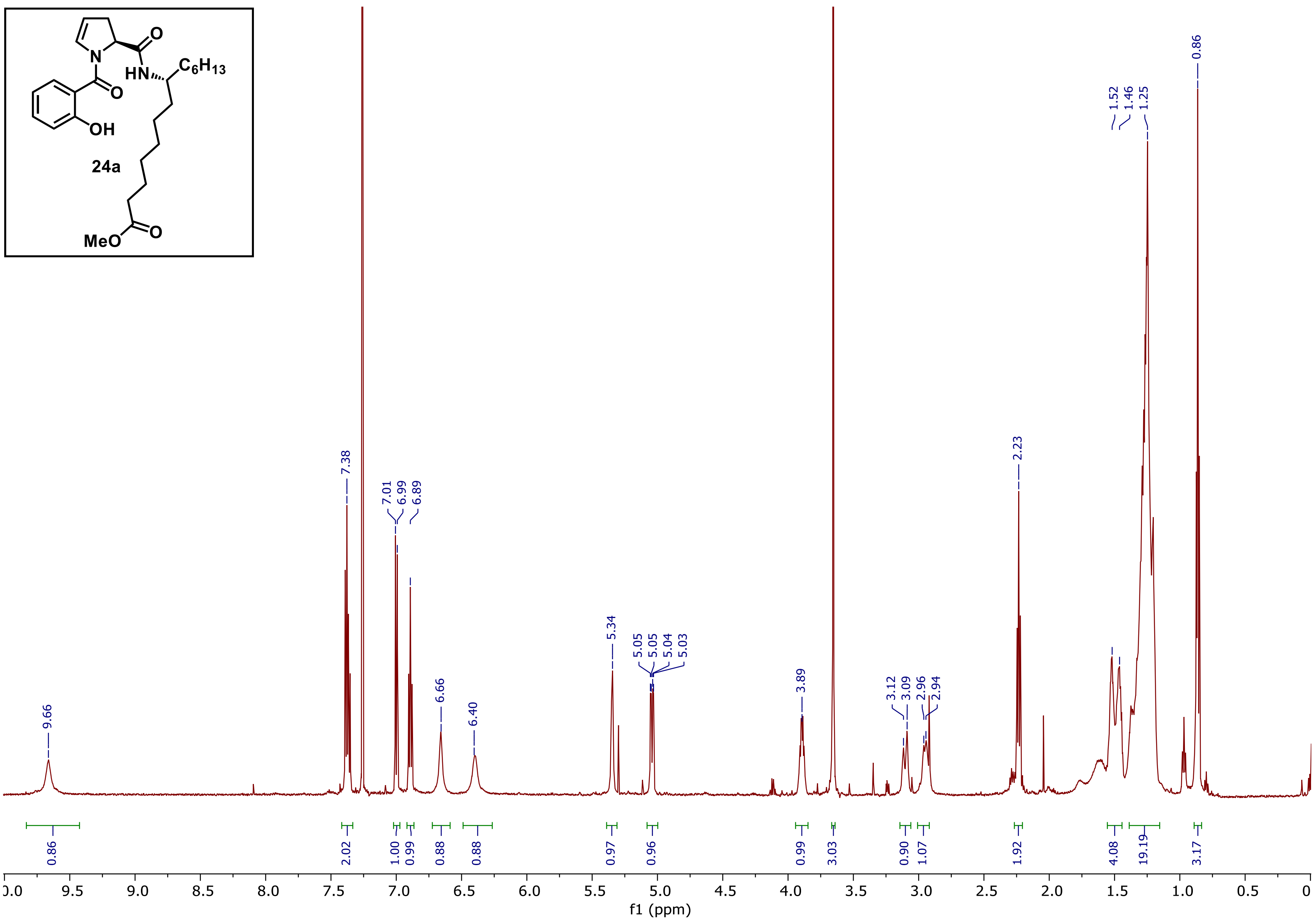

24a
MeO
OH
HN
N
O
C6H13
9.66
7.38
7.01
6.99
6.89
6.66
6.40
5.34
5.05
5.05
5.04
5.03
3.89
3.12
3.09
2.96
2.94
2.23
1.52
1.46
1.25
0.86
0.86
2.02
1.00
0.99
0.88
0.88
0.97
0.96
0.99
3.03
0.90
1.07
1.92
4.08
19.19
3.17
f1 (ppm)

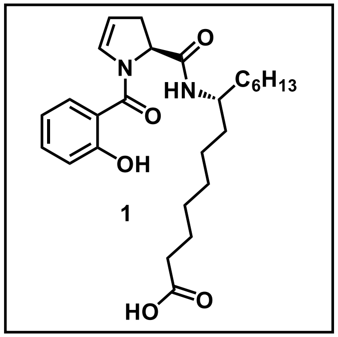

O
N
HN
C6H13
O
OH
HO
O

1

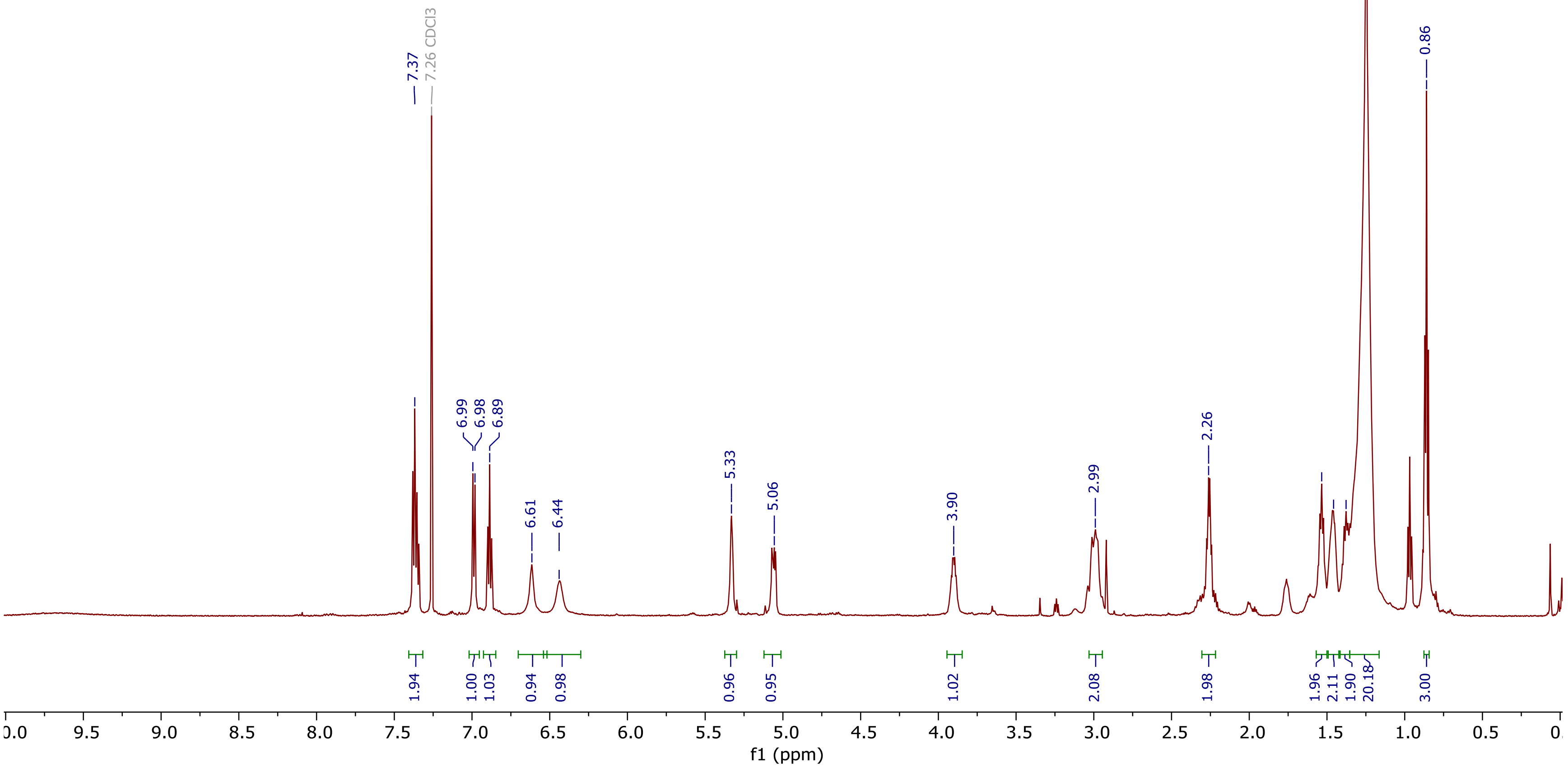

7.37
7.26 CDCl3
6.99
6.98
6.89
6.61
6.44
5.33
5.06
3.90
2.99
2.26
1.53
1.46
1.38
1.25
0.86
1.94
1.00
1.03
0.94
0.98
0.96
0.95
1.02
2.08
1.98
1.96
2.11
1.90
20.18
3.00
9.5
9.0
8.5
8.0
7.5
7.0
6.5
6.0
5.5
5.0
4.5
4.0
3.5
3.0
2.5
2.0
1.5
1.0
0.5
f1 (ppm)

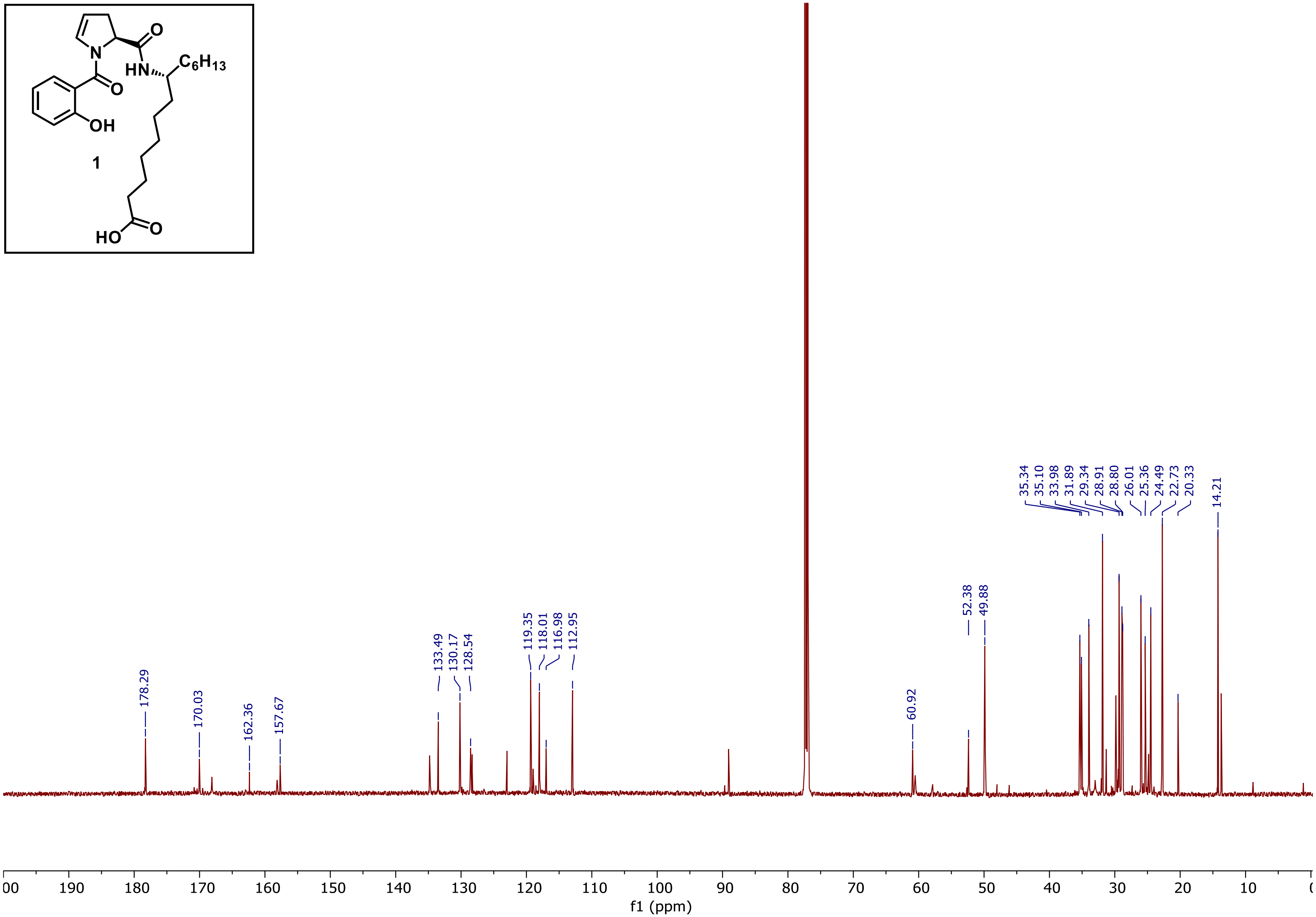

178.29
170.03
162.36
157.67
133.49
130.17
128.54
119.35
118.01
116.98
112.95
60.92
52.38
49.88
35.34
35.10
33.98
31.89
29.34
28.91
28.80
26.01
25.36
24.49
22.73
20.33
14.21
f1 (ppm)

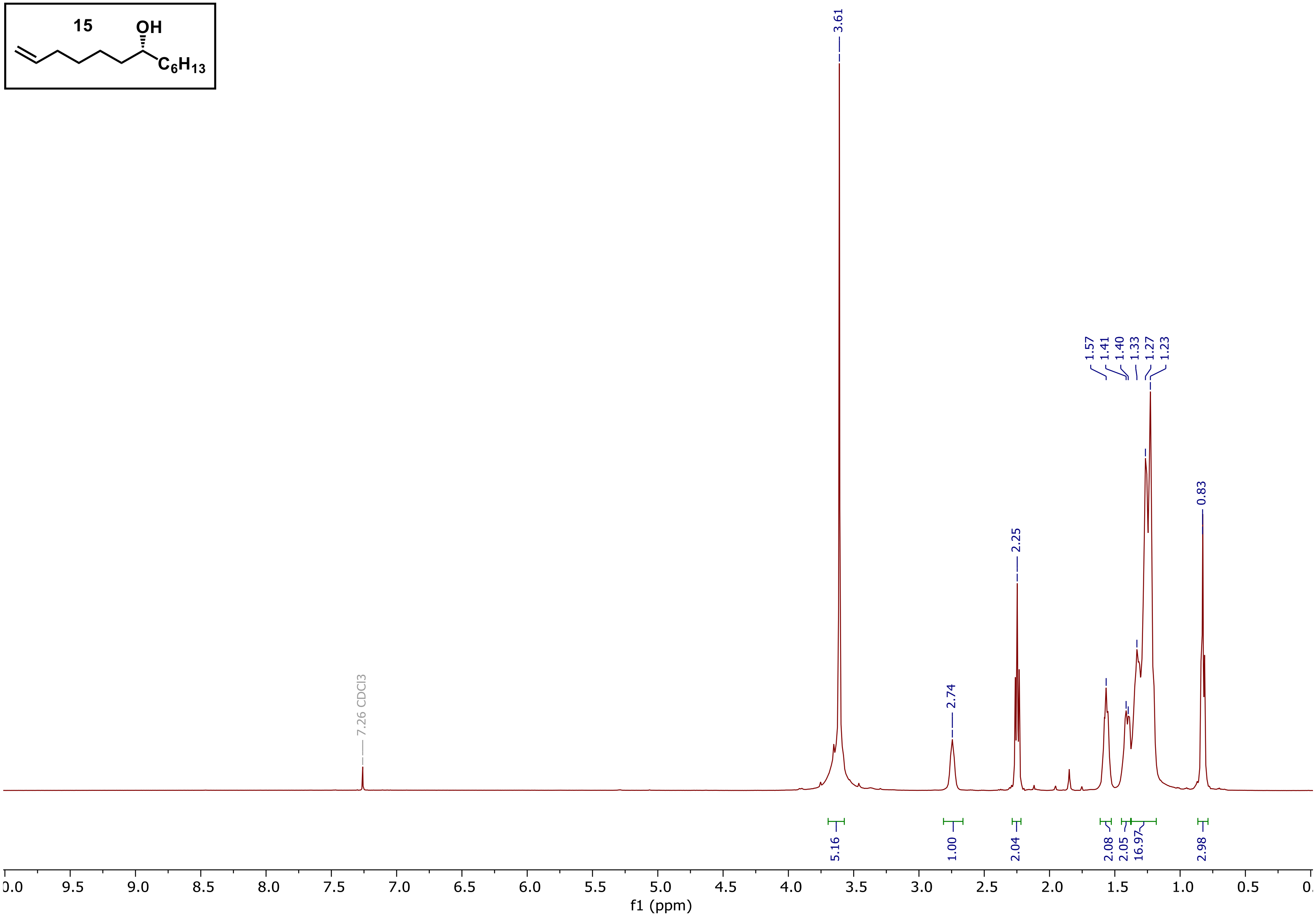

15
OH
C6H13
3.61
2.74
2.25
1.57
1.41
1.40
1.33
1.27
1.23
0.83
7.26 CDCl3
5.16
1.00
2.04
2.08
2.05
16.97
2.98
10.0
9.5
9.0
8.5
8.0
7.5
7.0
6.5
6.0
5.5
5.0
4.5
4.0
3.5
3.0
2.5
2.0
1.5
1.0
0.5
0.0
f1 (ppm)

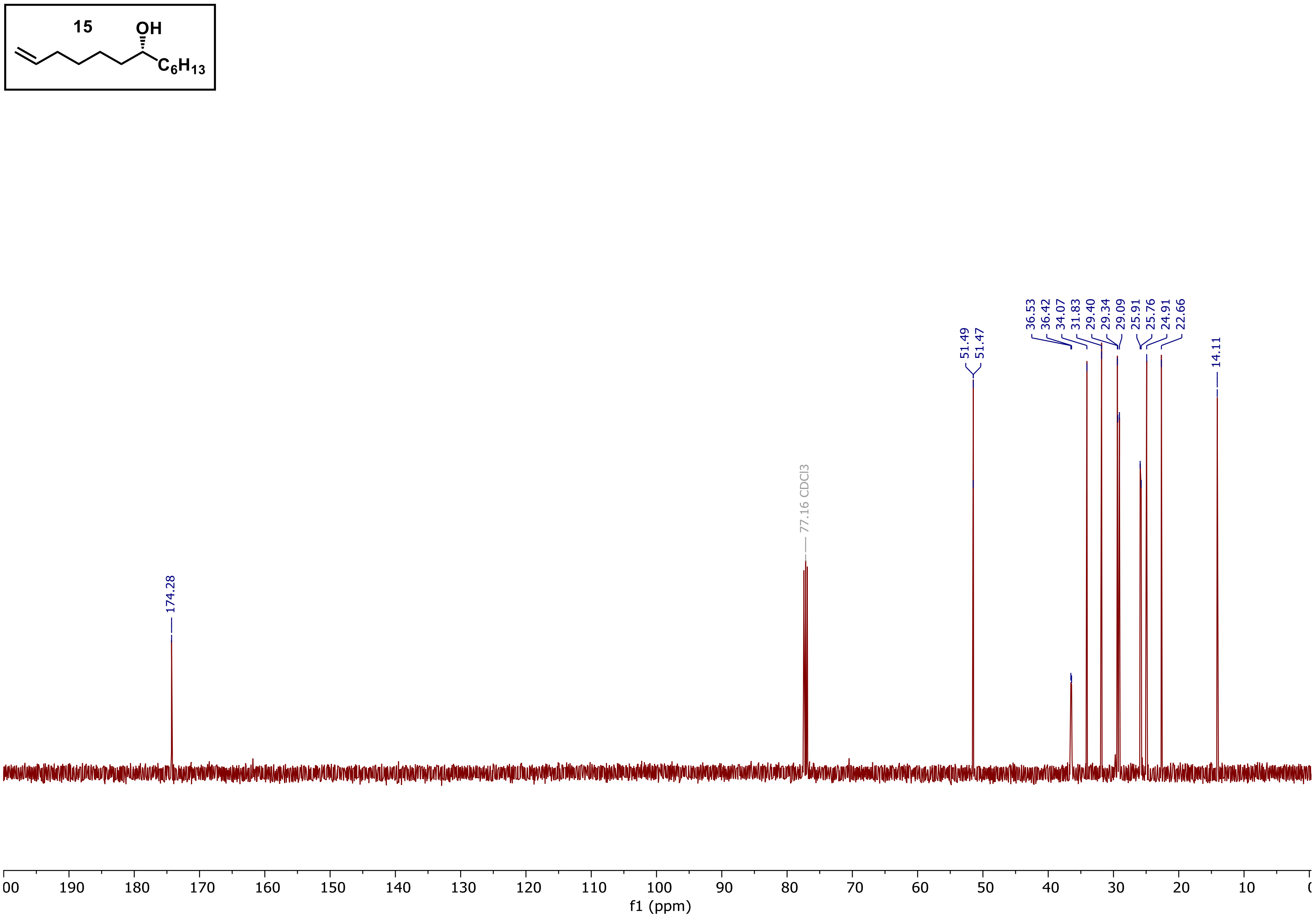
15
OH
C6H13
174.28
77.16 CDCl3
51.49
51.47
36.53
36.42
34.07
31.83
29.40
29.34
29.09
25.91
25.76
24.91
22.66
14.11
f1 (ppm)

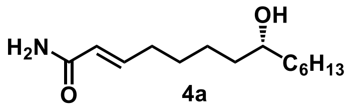

4a

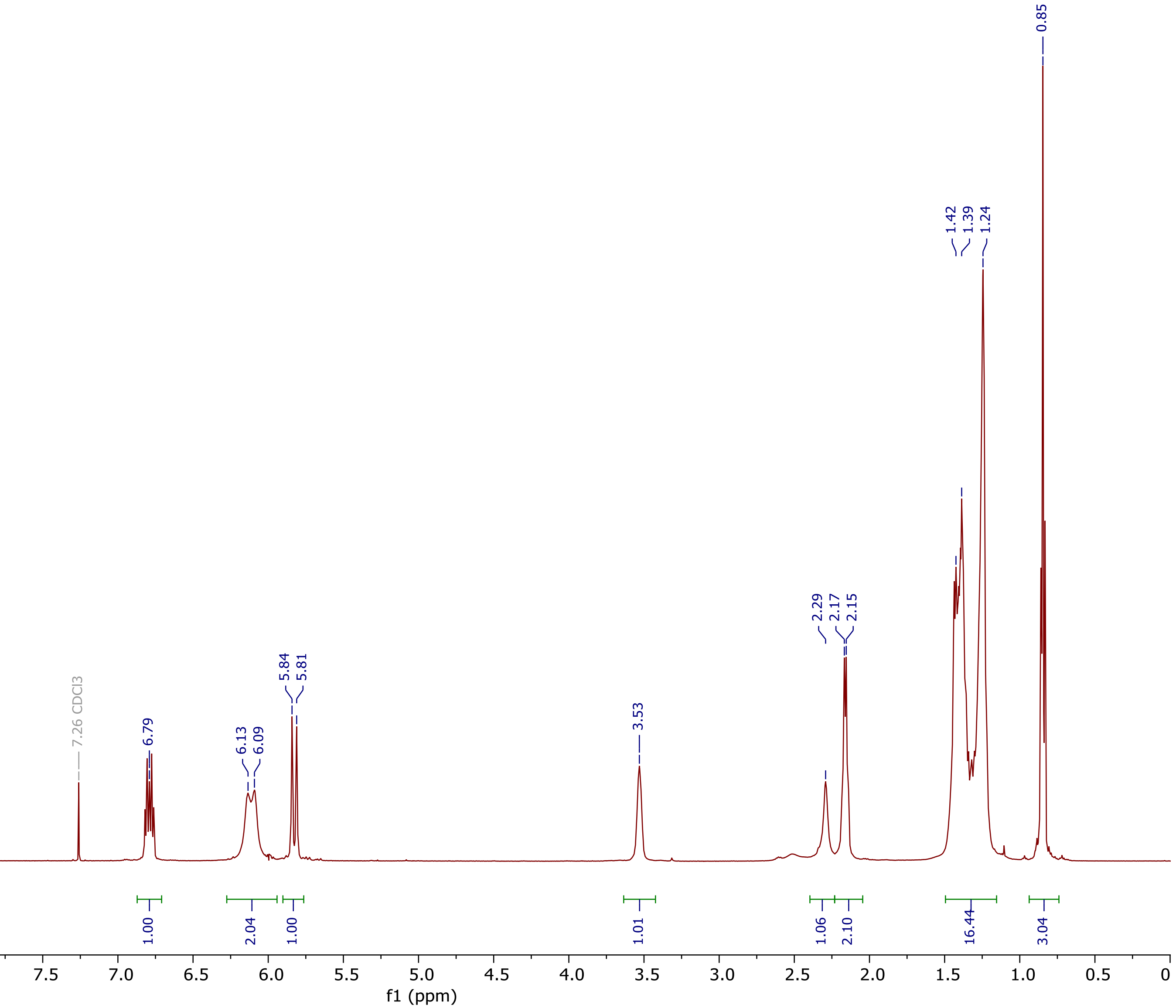
7.26 CDCl3
6.79
6.13
6.09
5.84
5.81
3.53
2.29
2.17
2.15
1.42
1.39
1.24
0.85
1.00
2.04
1.00
1.01
1.06
2.10
16.44
3.04
f1 (ppm)

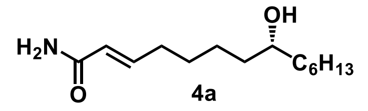

OH
H_2N
C_6H_{13}
O

4a

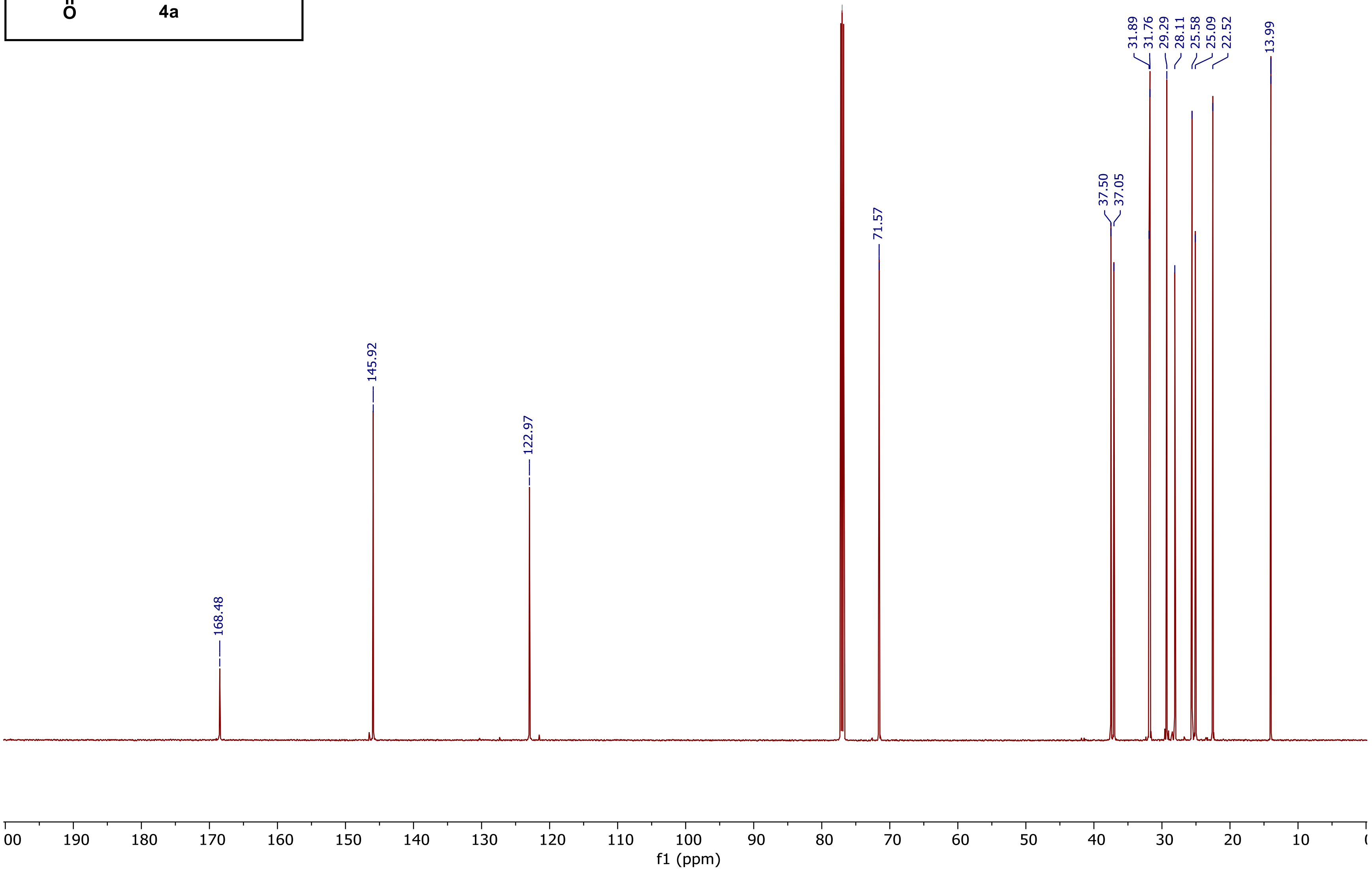

168.48
145.92
122.97
71.57
37.50
37.05
31.89
31.76
29.29
28.11
25.58
25.09
22.52
13.99
f1 (ppm)

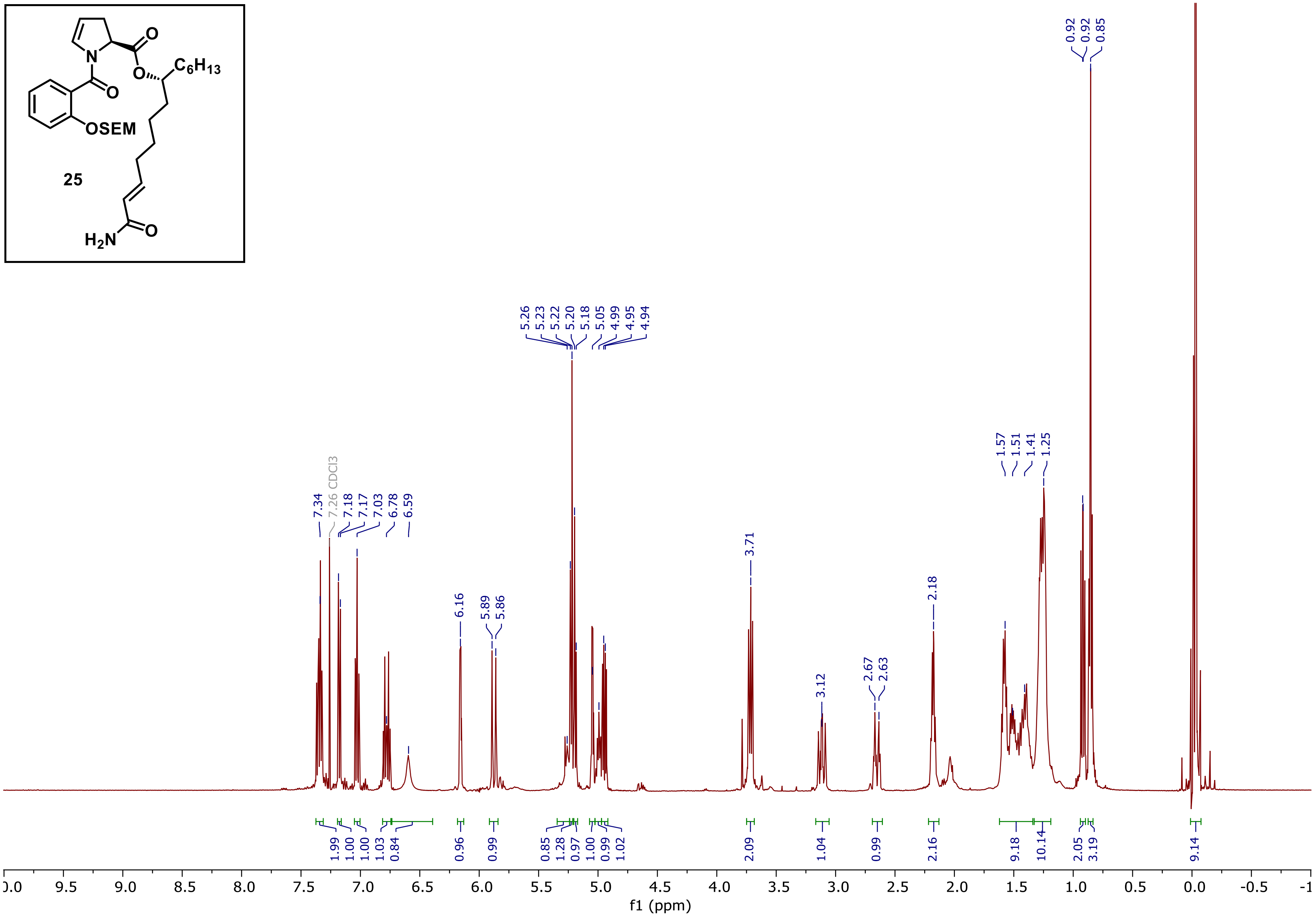

OSEM
C6H13
H2N
25
7.34
7.26 CDCl3
7.18
7.17
7.03
6.78
6.59
6.16
5.89
5.86
5.26
5.23
5.22
5.20
5.18
5.05
4.99
4.95
4.94
3.71
3.12
2.67
2.63
2.18
1.57
1.51
1.41
1.25
0.92
0.92
0.85
1.99
1.00
1.00
1.03
0.84
0.96
0.99
0.85
1.28
0.97
1.00
0.99
1.02
2.09
1.04
0.99
2.16
9.18
10.14
2.05
3.19
9.14
f1 (ppm)

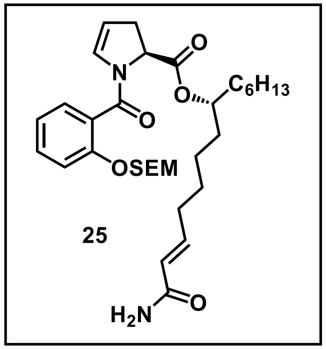
OSEM
C6H13
H2N

25

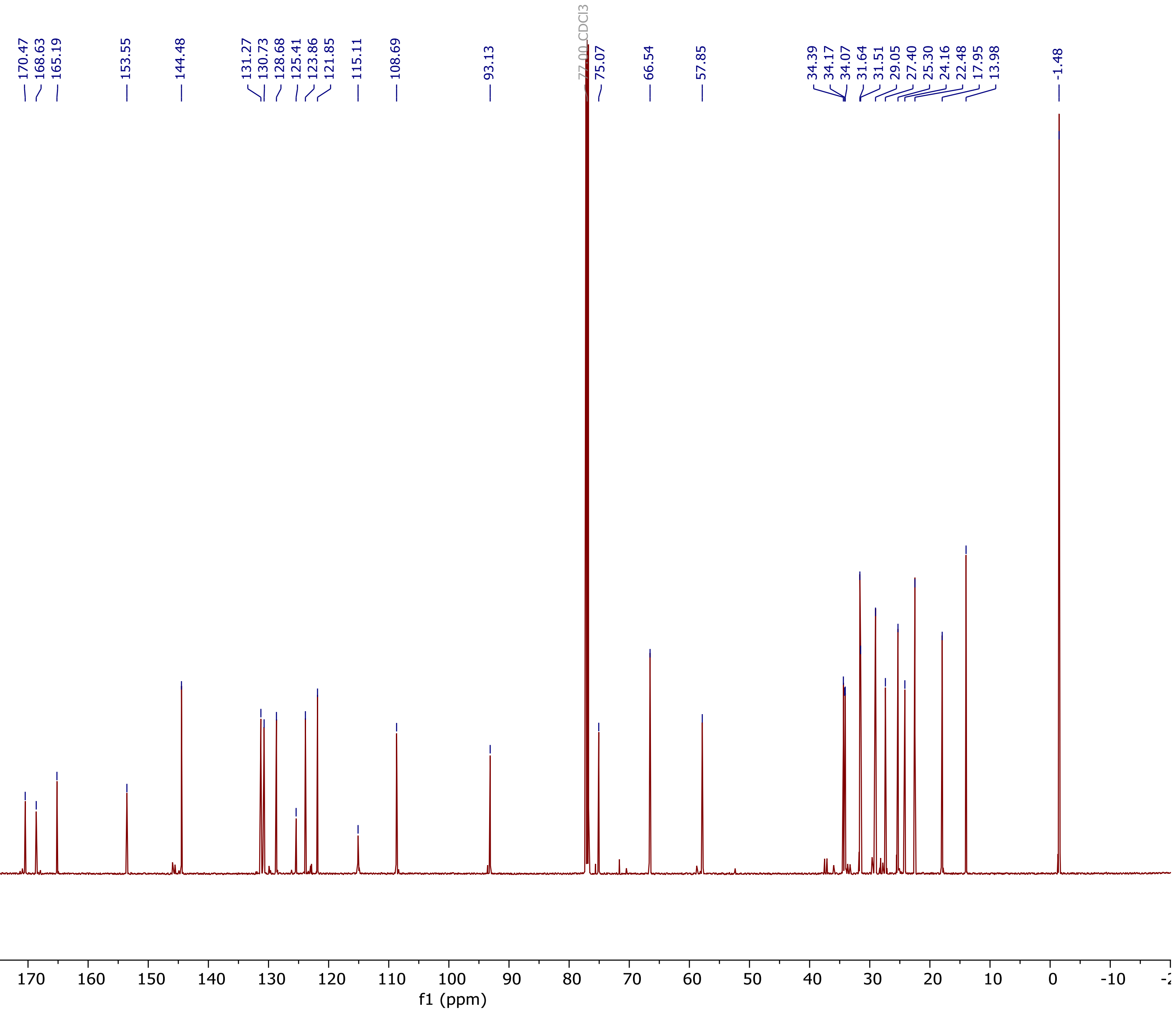
170.47
168.63
165.19
153.55
144.48
131.27
130.73
128.68
125.41
123.86
121.85
115.11
108.69
93.13
77.00 CDCl3
75.07
66.54
57.85
34.39
34.17
34.07
31.64
31.51
29.05
27.40
25.30
24.16
22.48
17.95
13.98
-1.48
210 200 190 180 170 160 150 140 130 120 110 100 90 80 70 60 50 40 30 20 10 0 -10
f1 (ppm)

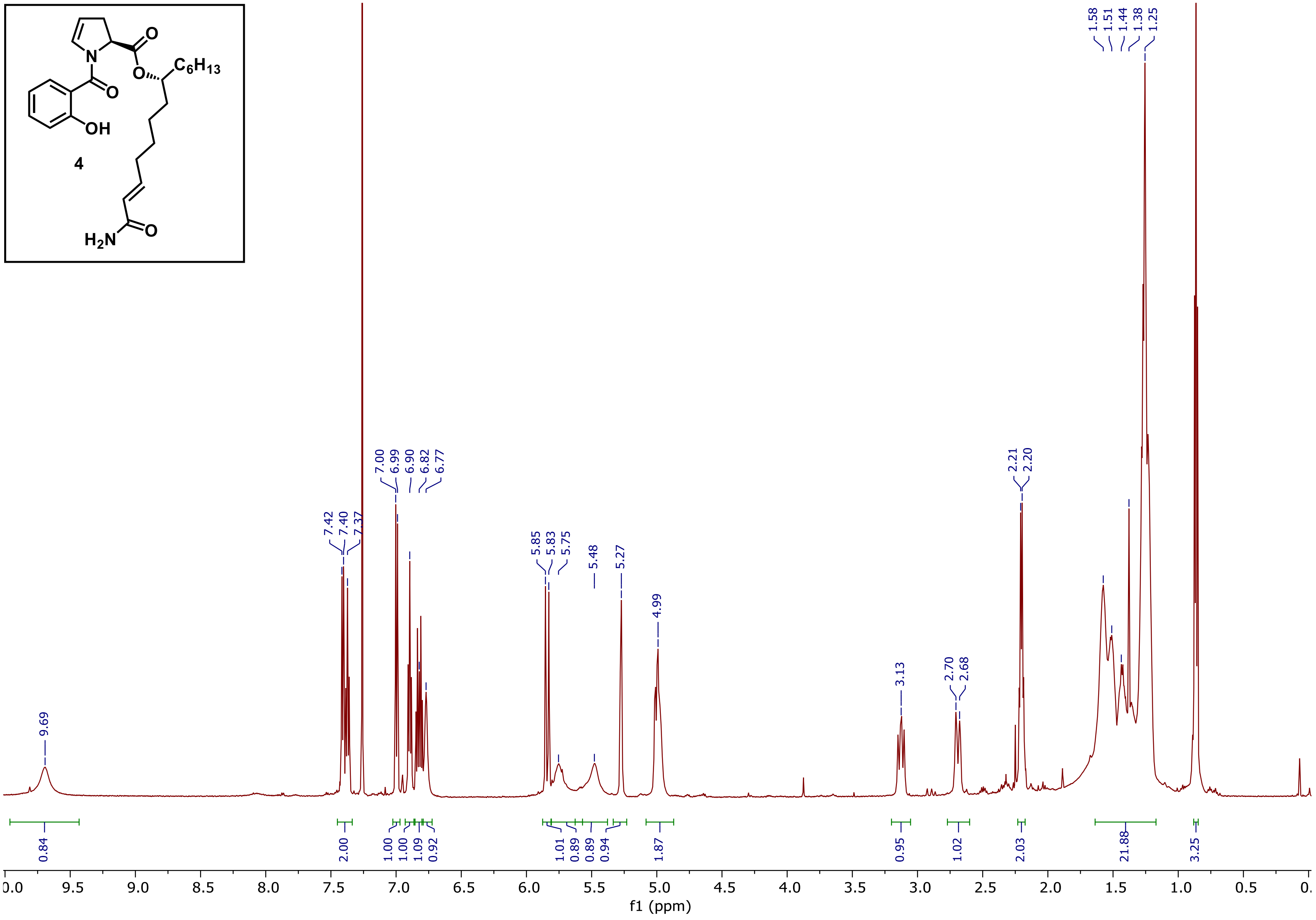

OH
O
N
O
O
C_6H_{13}
H_2N
4
9.69
7.42
7.40
7.37
7.00
6.99
6.90
6.82
6.77
5.85
5.83
5.75
5.48
5.27
4.99
3.13
2.70
2.68
2.21
2.20
1.58
1.51
1.44
1.38
1.25
0.84
2.00
1.00
1.00
1.09
0.92
1.01
0.89
0.89
0.94
1.87
0.95
1.02
2.03
21.88
3.25
f1 (ppm)

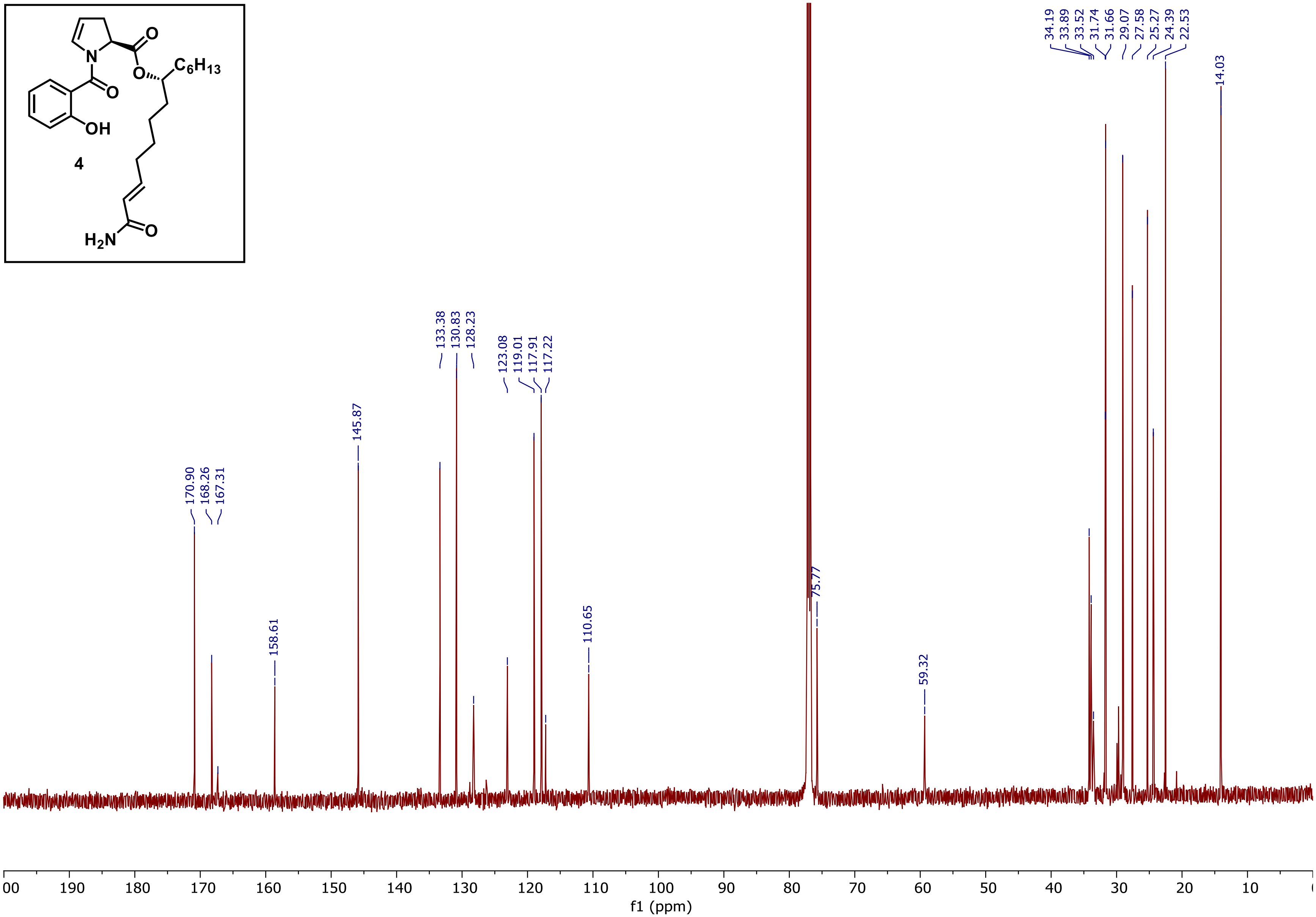
O
N
O
C6H13
O
OH
H2N
O
4
170.90
168.26
167.31
158.61
145.87
133.38
130.83
128.23
123.08
119.01
117.91
117.22
110.65
75.77
59.32
34.19
33.89
33.52
31.74
31.66
29.07
27.58
25.27
24.39
22.53
14.03
f1 (ppm)

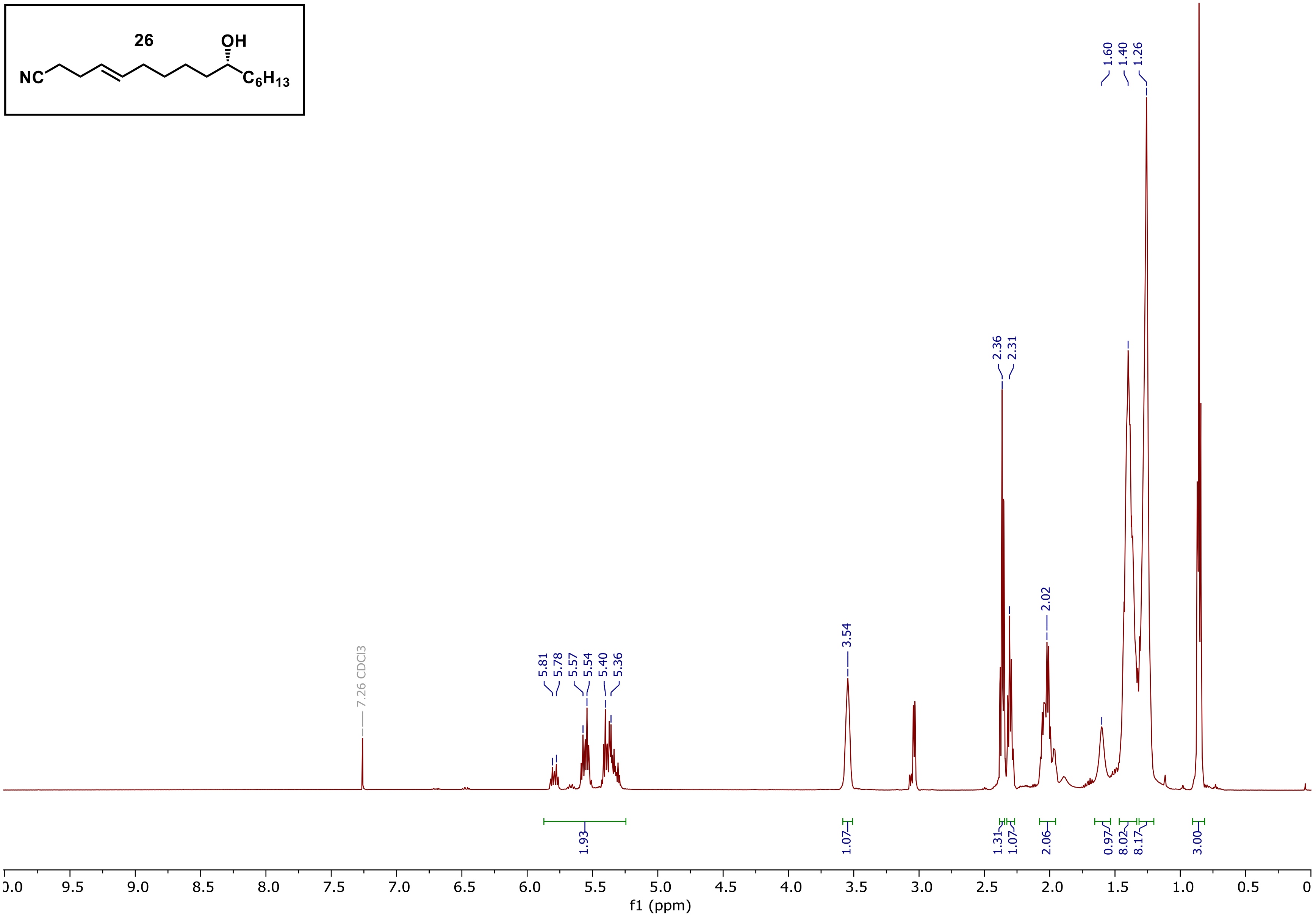

26
OH
NC
C_6H_{13}
7.26 CDCl3
5.81
5.78
5.57
5.54
5.40
5.36
3.54
2.36
2.31
2.02
1.60
1.40
1.26
1.93
1.07
1.31
1.07
2.06
0.97
8.02
8.17
3.00
f1 (ppm)

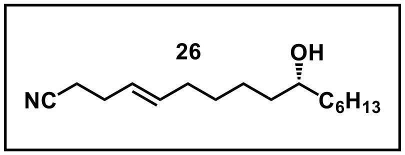
26
OH
NC
C_6H_{13}

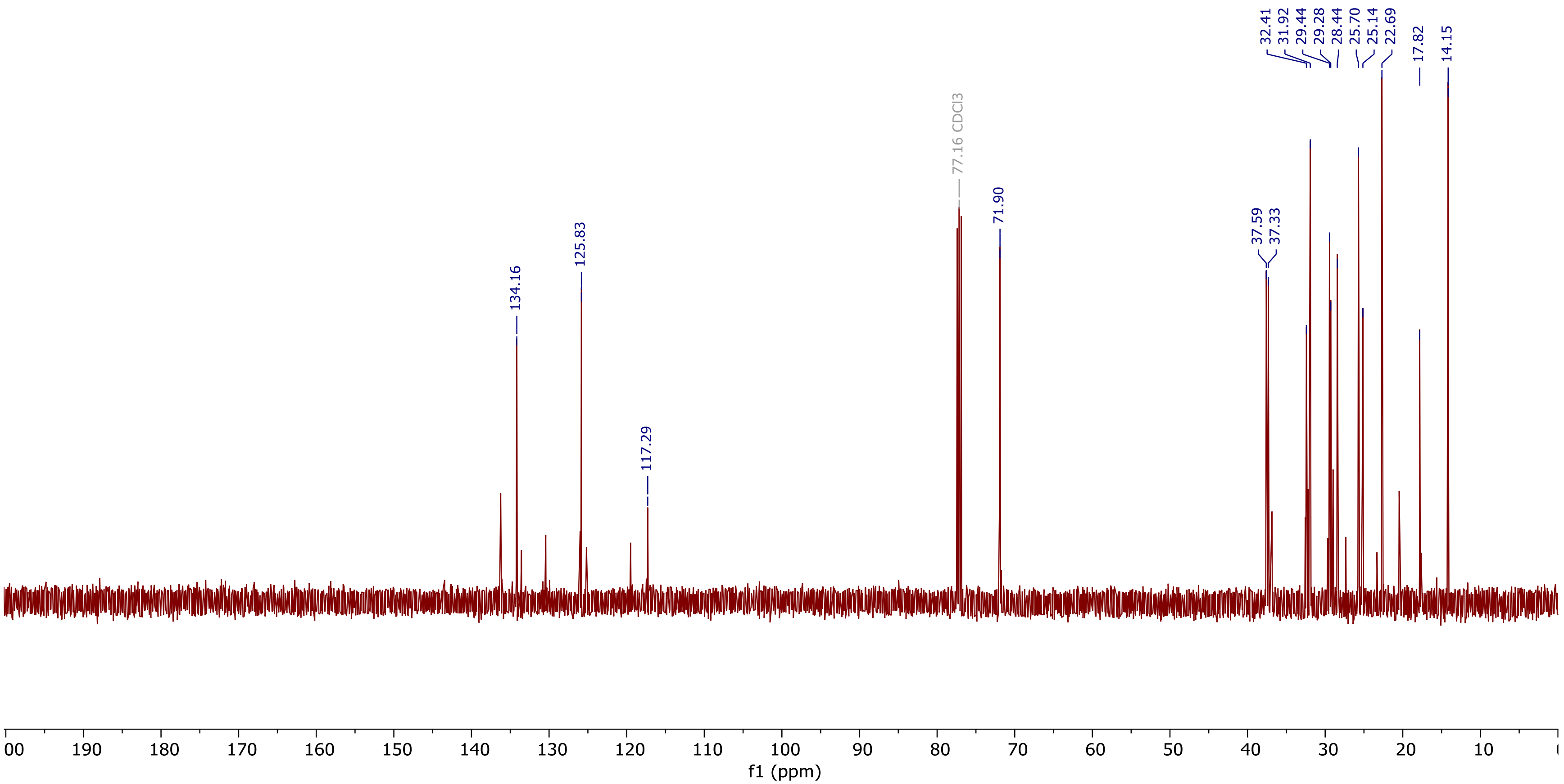
32.41
31.92
29.44
29.28
28.44
25.70
25.14
22.69
17.82
14.15
77.16 CDCl3
71.90
37.59
37.33
134.16
125.83
117.29
00
190
180
170
160
150
140
130
120
110
100
90
80
70
60
50
40
30
20
10
f1 (ppm)

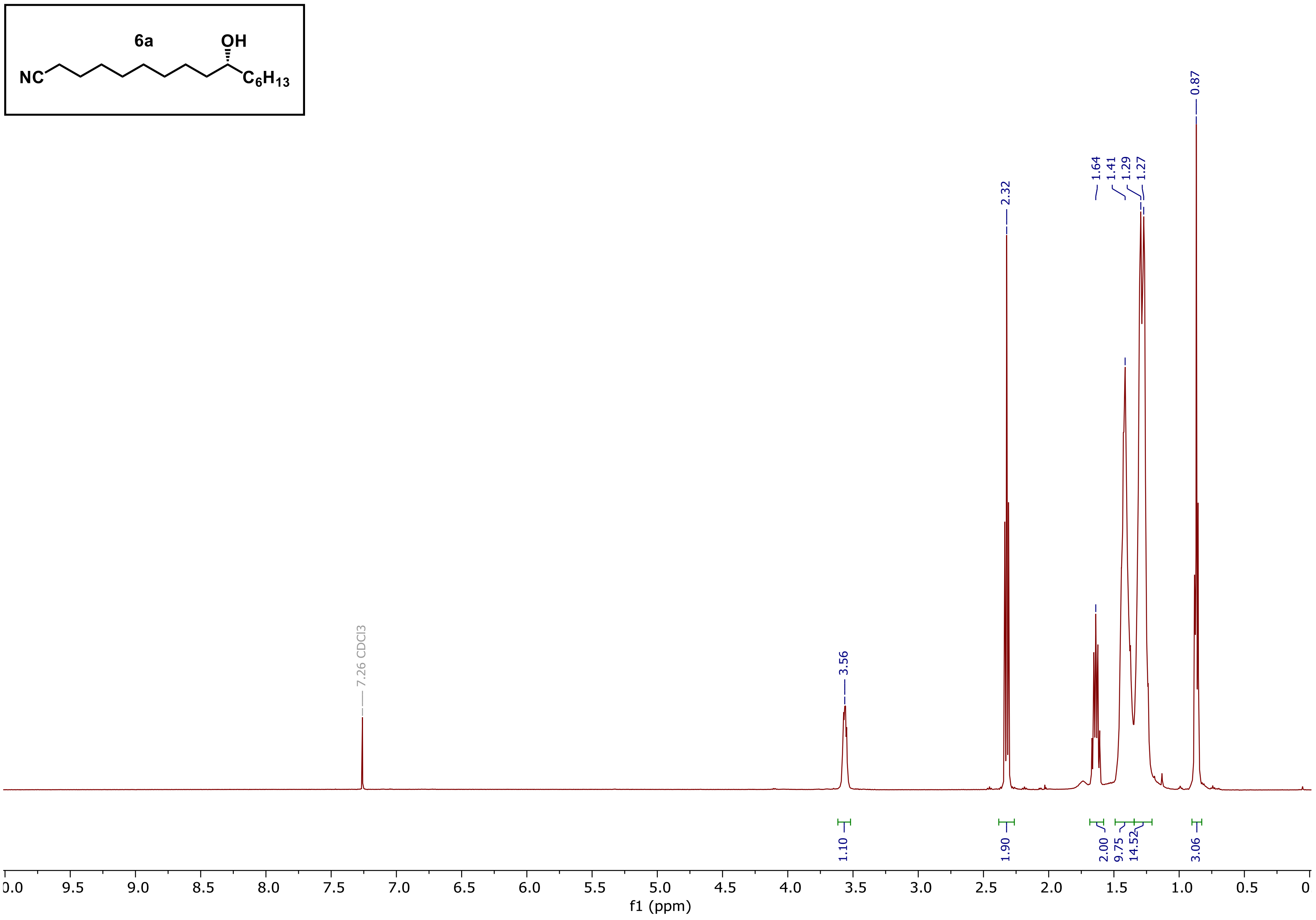

6a
OH
NC
C6H13
0.87
1.64
1.41
1.29
1.27
2.32
7.26 CDCl3
3.56
1.10
1.90
2.00
9.75
14.52
3.06
10.0
9.5
9.0
8.5
8.0
7.5
7.0
6.5
6.0
5.5
5.0
4.5
4.0
3.5
3.0
2.5
2.0
1.5
1.0
0.5
0
f1 (ppm)

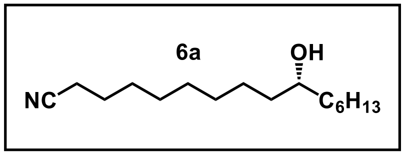
6a
OH
NC
C6H13

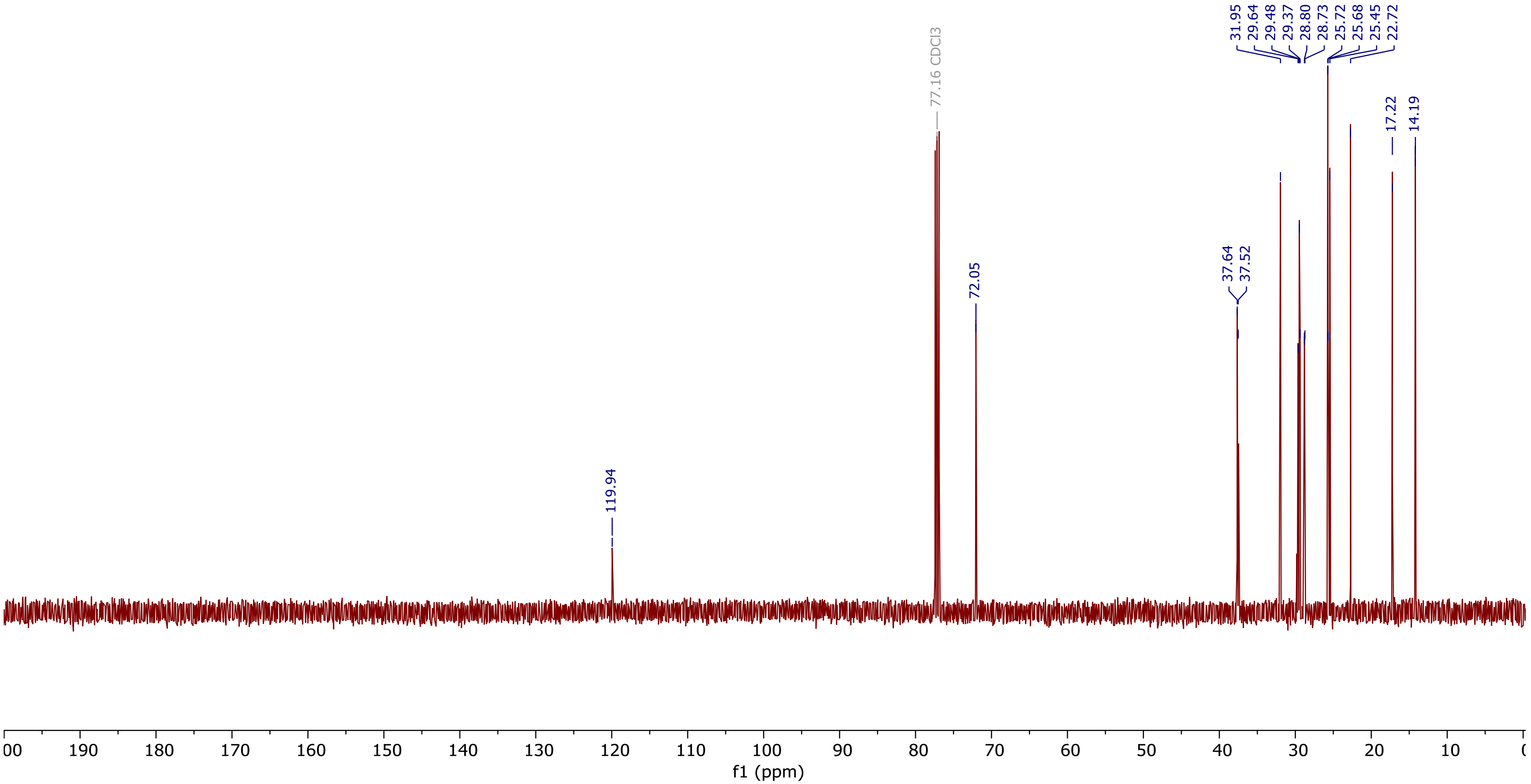
119.94
77.16 CDCl3
72.05
37.64
37.52
31.95
29.64
29.48
29.37
28.80
28.73
25.72
25.68
25.45
22.72
17.22
14.19
f1 (ppm)

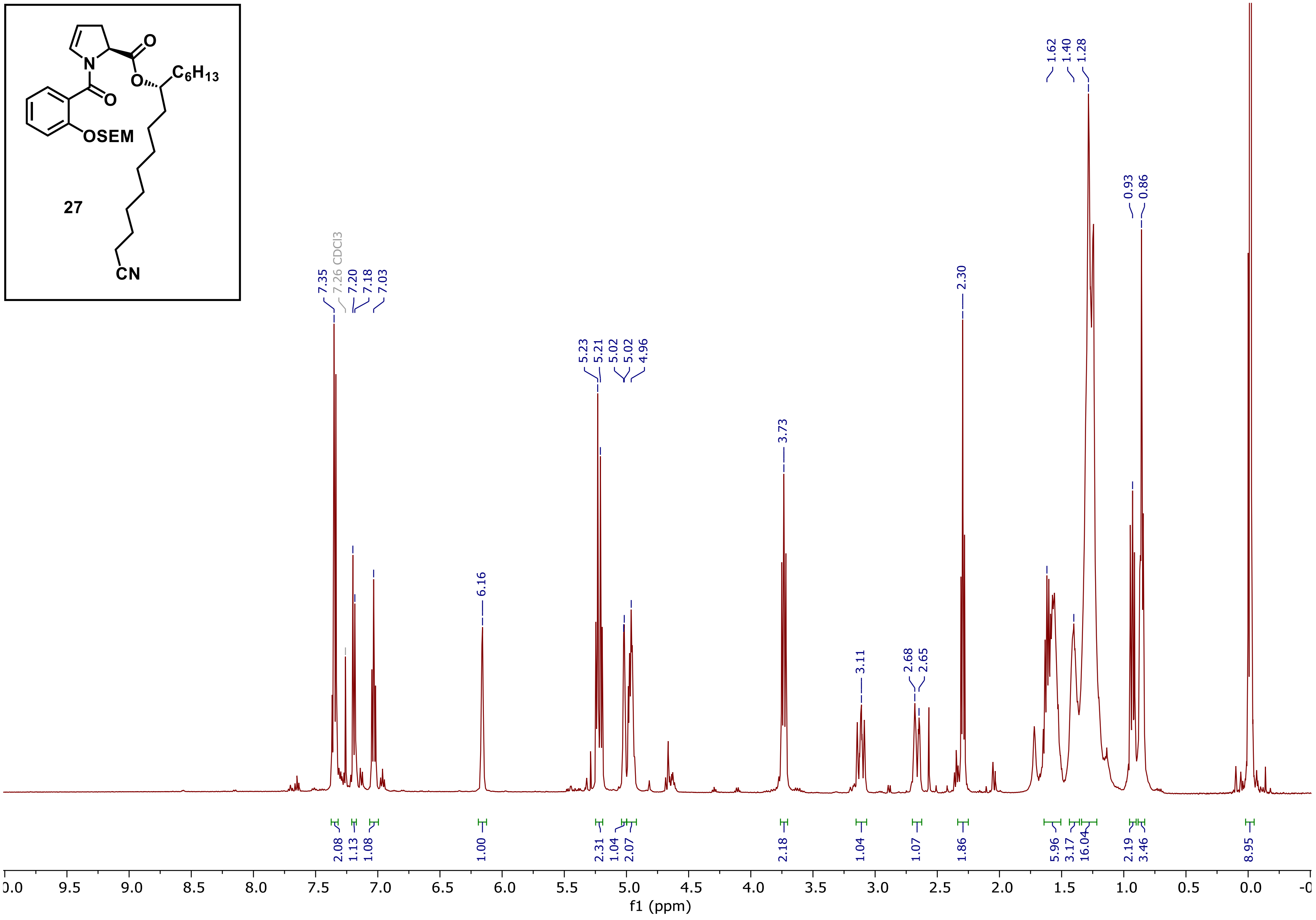
27
OSEM
CN
f1 (ppm)
7.26 CDCl3

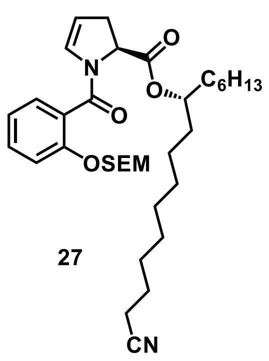

O
N
O
C6H13
O
OSEM
CN

27

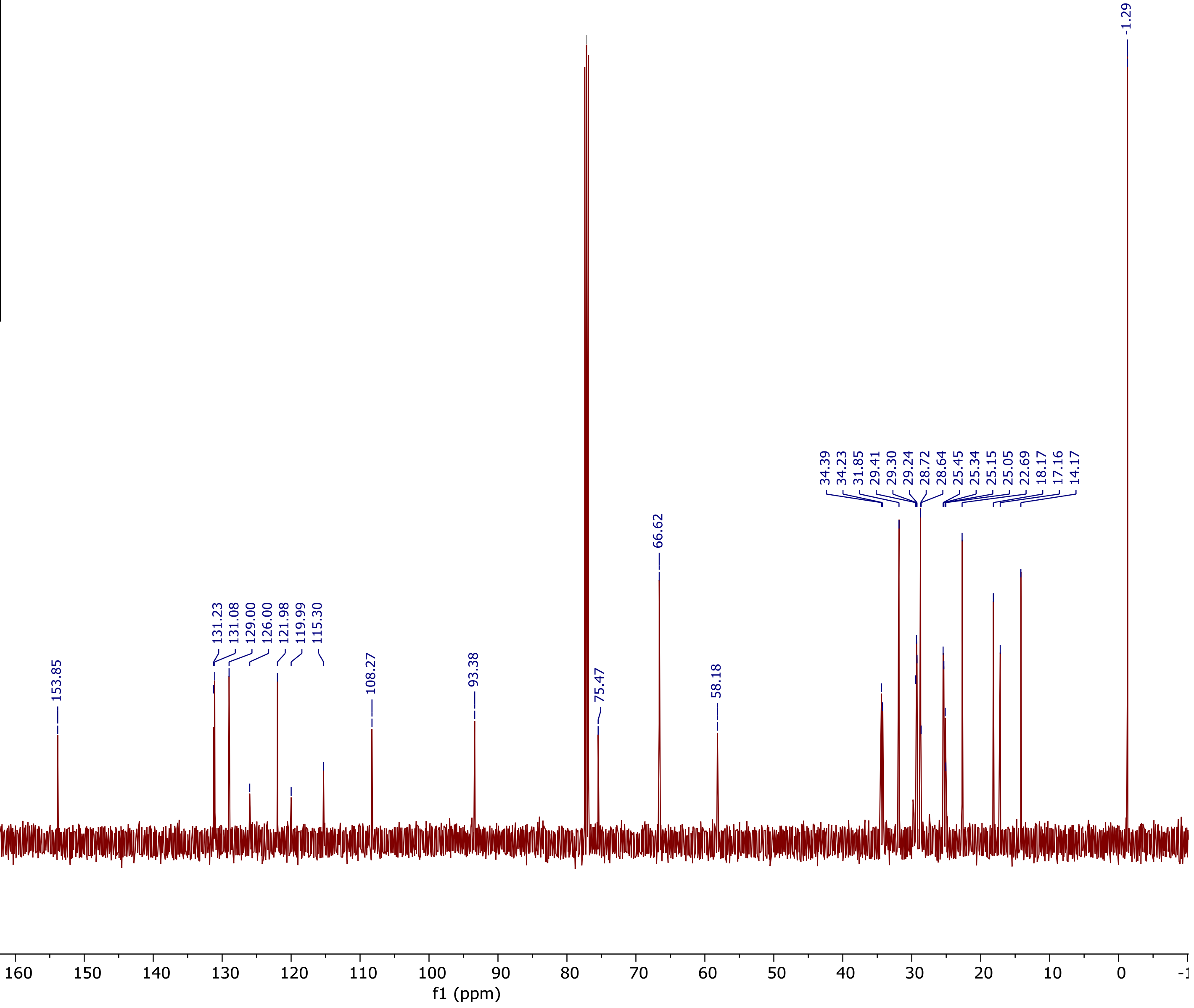

-1.29
34.39
34.23
31.85
29.41
29.30
29.24
28.72
28.64
25.45
25.34
25.15
25.05
22.69
18.17
17.16
14.17
66.62
131.23
131.08
129.00
126.00
121.98
119.99
115.30
170.82
164.98
153.85
108.27
93.38
75.47
58.18
00
190
180
170
160
150
140
130
120
110
100
90
80
70
60
50
40
30
20
10
0
-1
f1 (ppm)

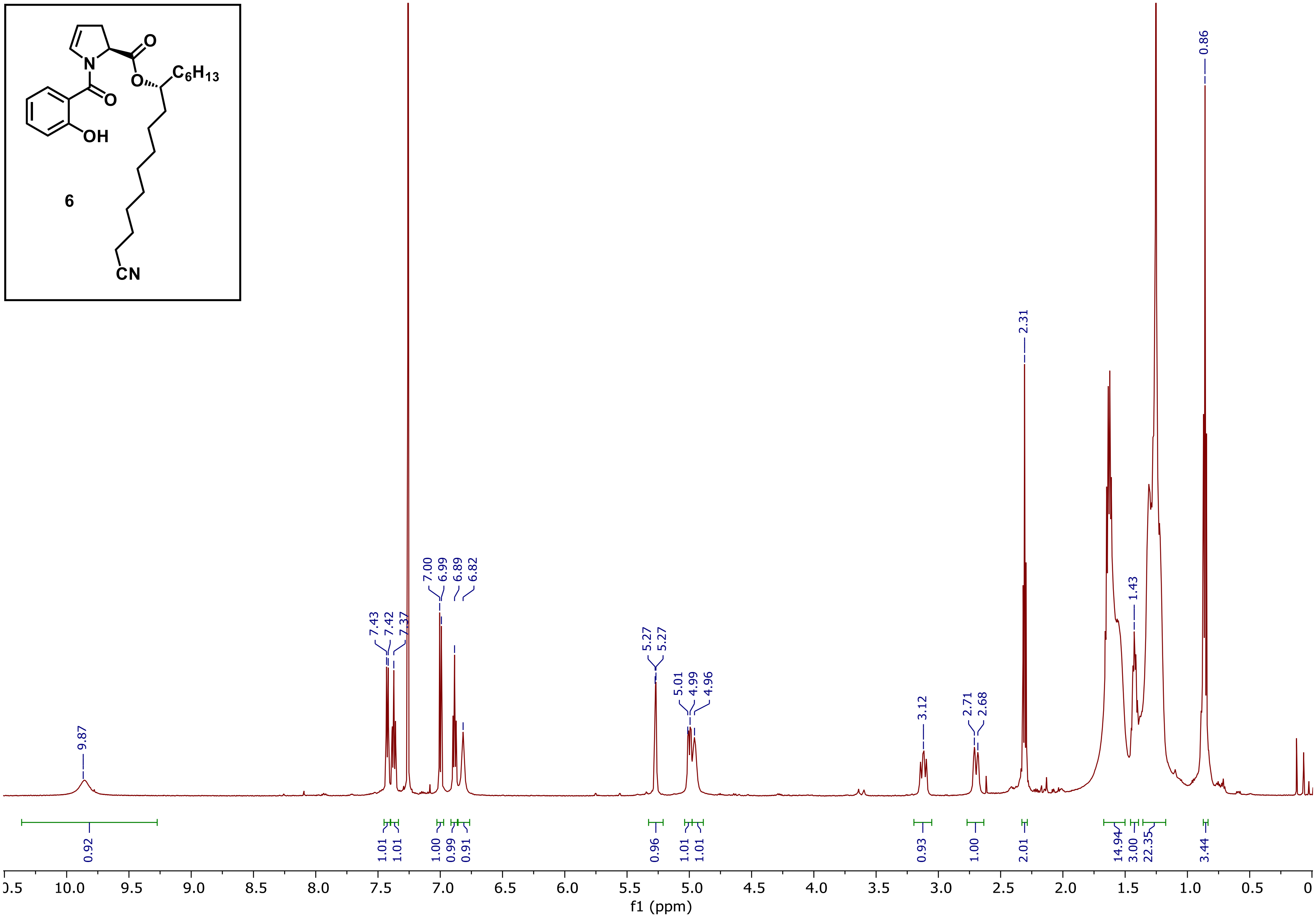

O
N
O
O
C6H13
OH
CN
6
f1 (ppm)

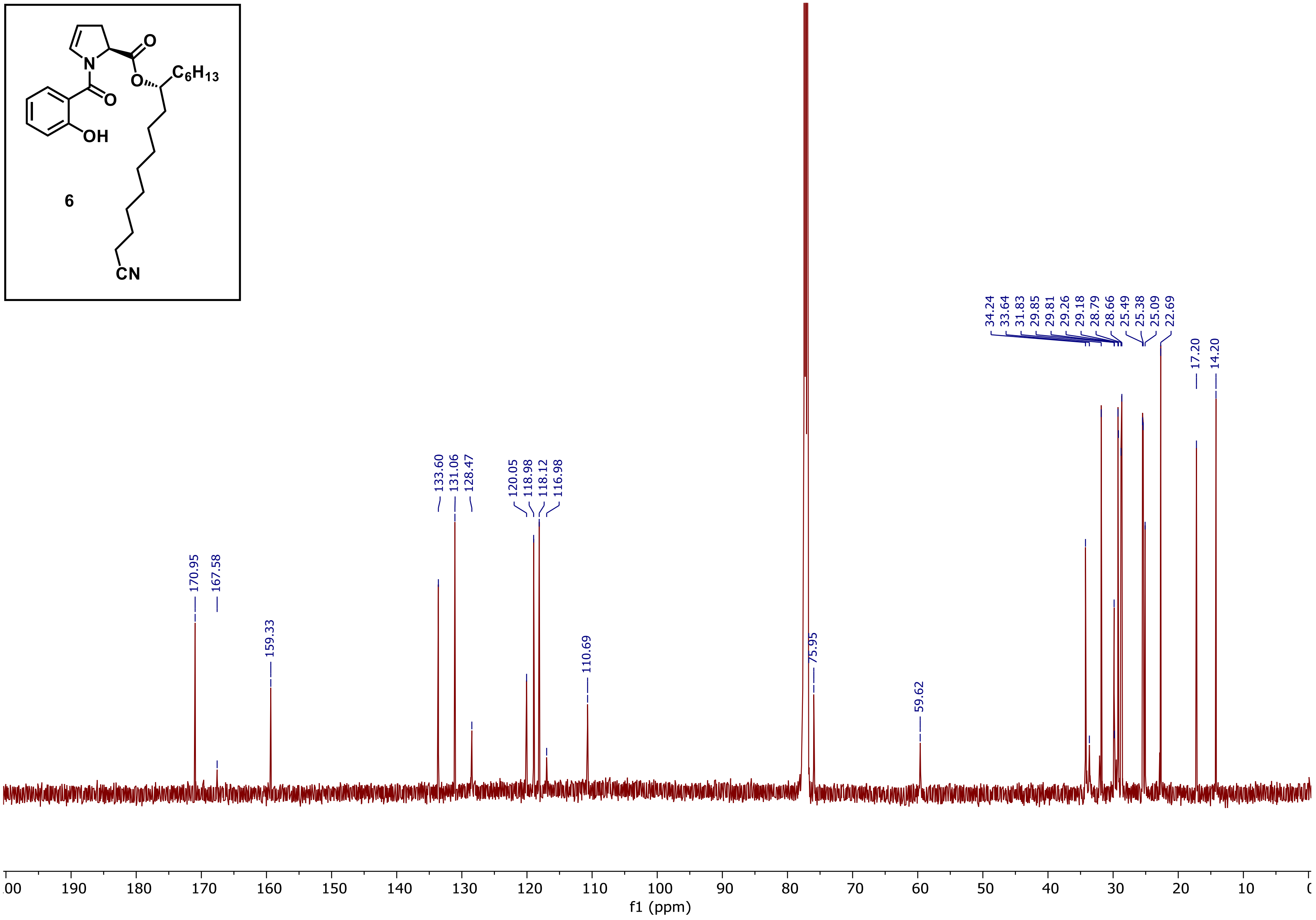
OH
O
N
O
O
C6H13
CN
6
170.95
167.58
159.33
133.60
131.06
128.47
120.05
118.98
118.12
116.98
110.69
75.95
59.62
34.24
33.64
31.83
29.85
29.81
29.26
29.18
28.79
28.66
25.49
25.38
25.09
22.69
17.20
14.20
f1 (ppm)

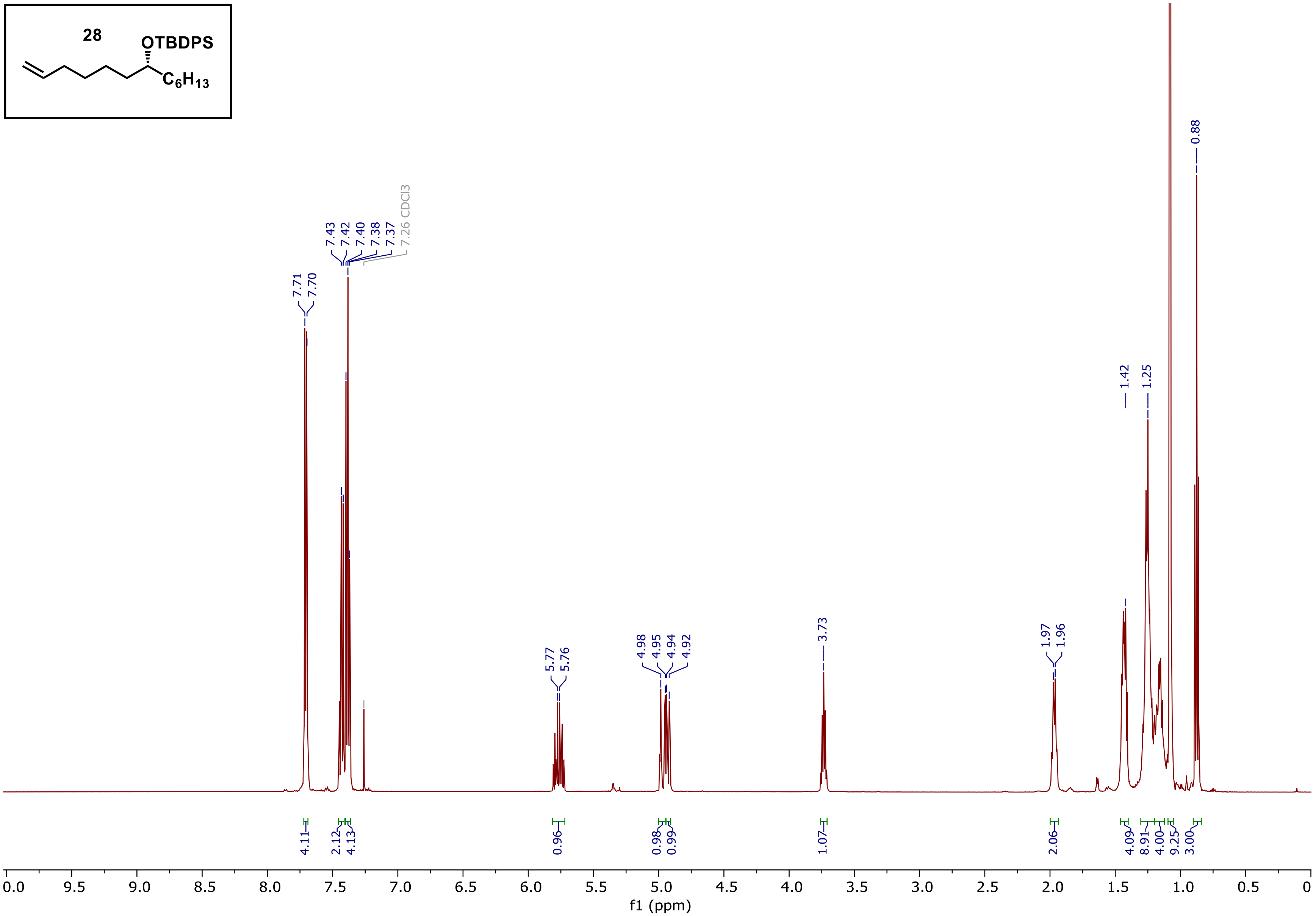
28
OTBDPS
C6H13
7.71
7.70
7.43
7.42
7.40
7.38
7.37
7.26 CDCl3
5.77
5.76
4.98
4.95
4.94
4.92
3.73
1.97
1.96
1.42
1.25
0.88
4.11
2.12
4.13
0.96
0.98
0.99
1.07
2.06
4.09
8.91
4.00
9.25
3.00
0.0
9.5
9.0
8.5
8.0
7.5
7.0
6.5
6.0
5.5
5.0
4.5
4.0
3.5
3.0
2.5
2.0
1.5
1.0
0.5
0
f1 (ppm)

28

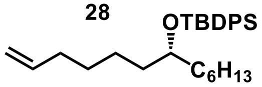
OTBDPS
C6H13

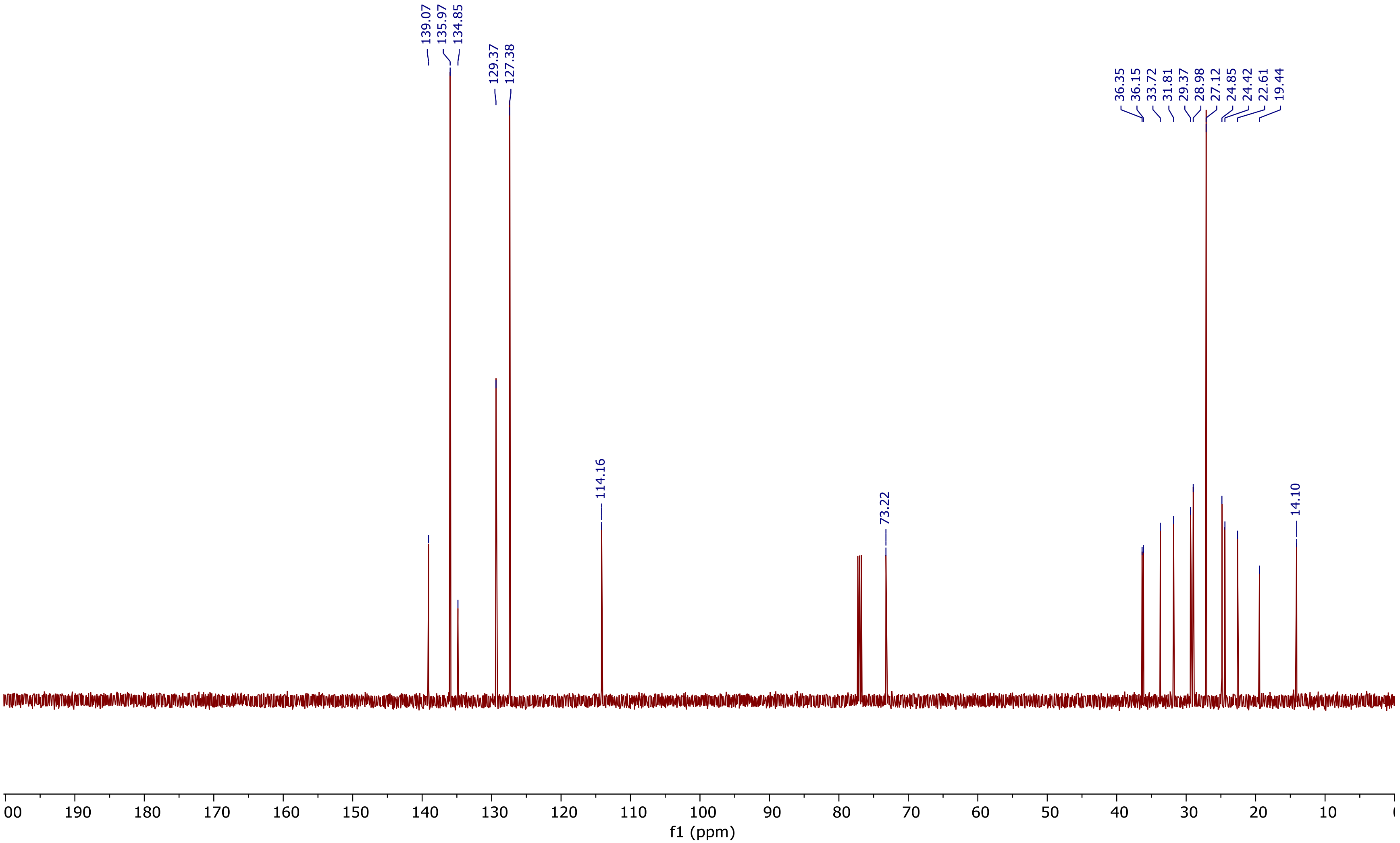
139.07
135.97
134.85
129.37
127.38
114.16
73.22
36.35
36.15
33.72
31.81
29.37
28.98
27.12
24.85
24.42
22.61
19.44
14.10
190
180
170
160
150
140
130
120
110
100
90
80
70
60
50
40
30
20
10
f1 (ppm)

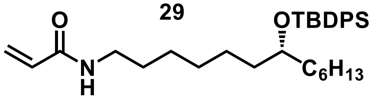

29

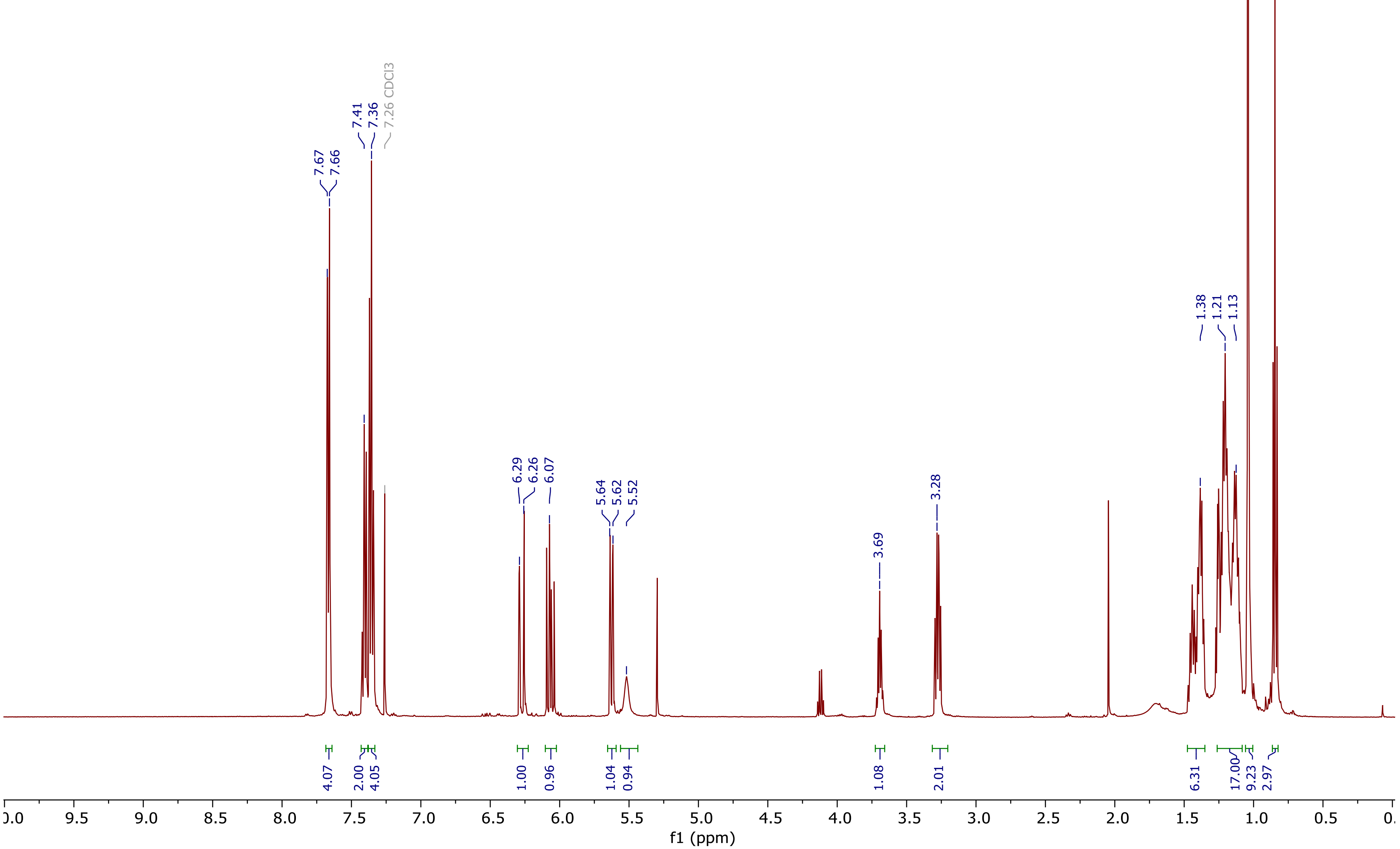

7.67
7.66
7.41
7.36
7.26 CDCl3
6.29
6.26
6.07
5.64
5.62
5.52
3.69
3.28
1.38
1.21
1.13
0.85
4.07
2.00
4.05
1.00
0.96
1.04
0.94
1.08
2.01
6.31
17.00
9.23
2.97
f1 (ppm)

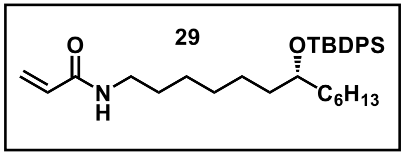
29
O
OTBDPS
N
H
C6H13

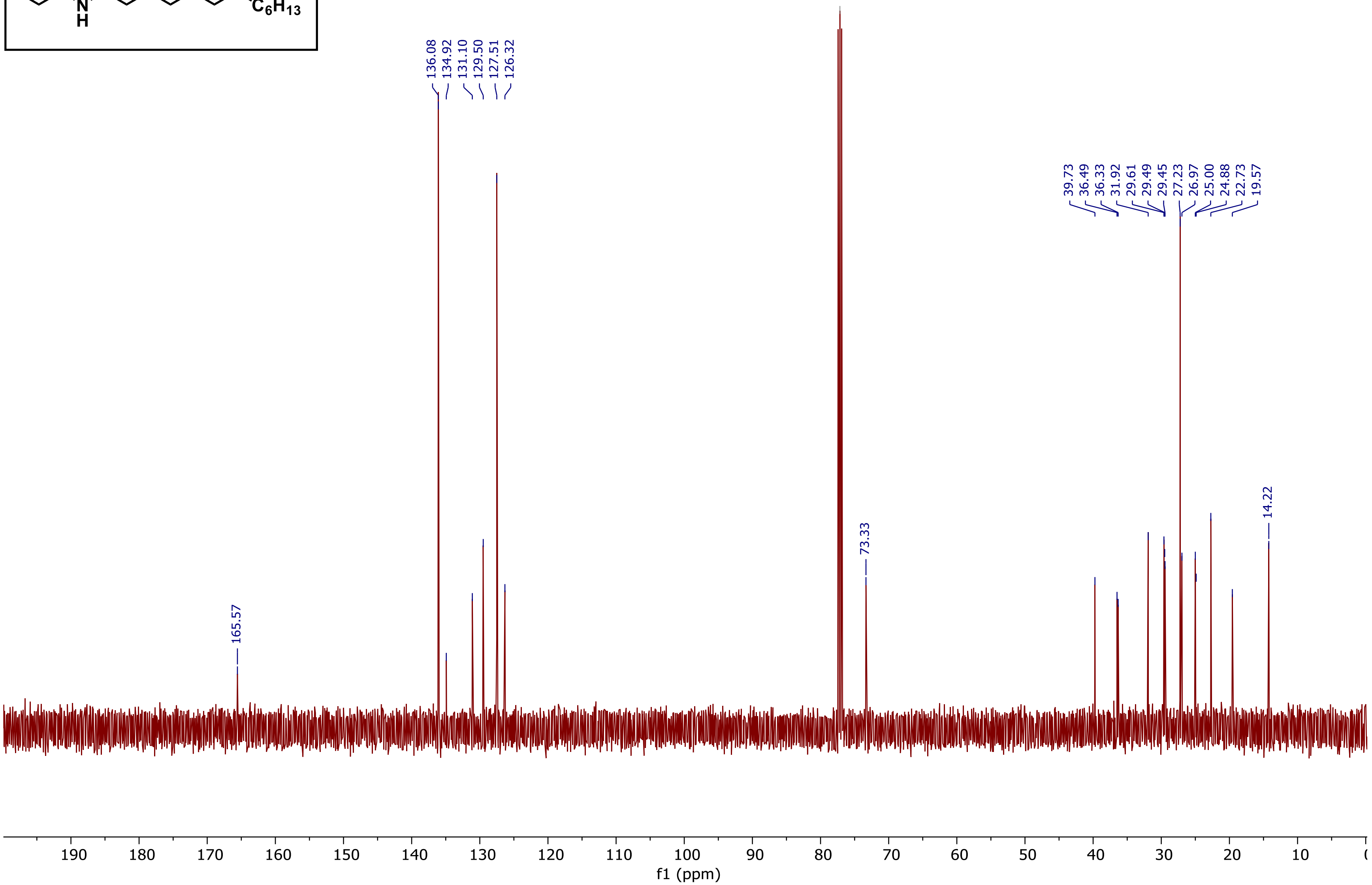
136.08
134.92
131.10
129.50
127.51
126.32
165.57
73.33
39.73
36.49
36.33
31.92
29.61
29.49
29.45
27.23
26.97
25.00
24.88
22.73
19.57
14.22
190 180 170 160 150 140 130 120 110 100 90 80 70 60 50 40 30 20 10 0
f1 (ppm)

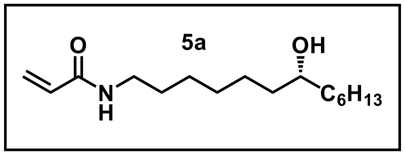
5a
OH
O
N
H
C6H13

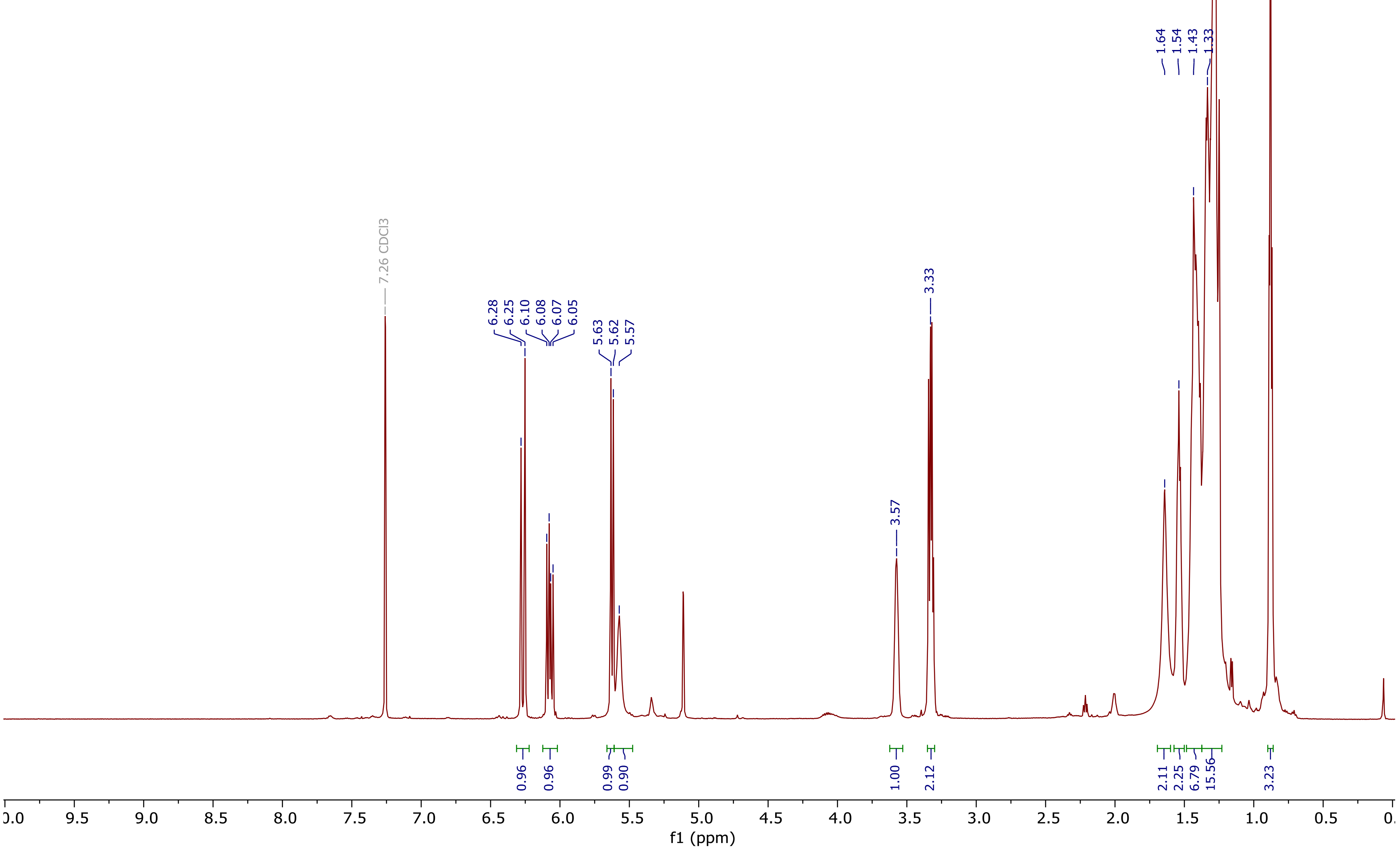
7.26 CDCl3
6.28
6.25
6.10
6.08
6.07
6.05
5.63
5.62
5.57
3.57
3.33
1.64
1.54
1.43
1.33
0.96
0.96
0.99
0.90
1.00
2.12
2.11
2.25
6.79
15.56
3.23
f1 (ppm)

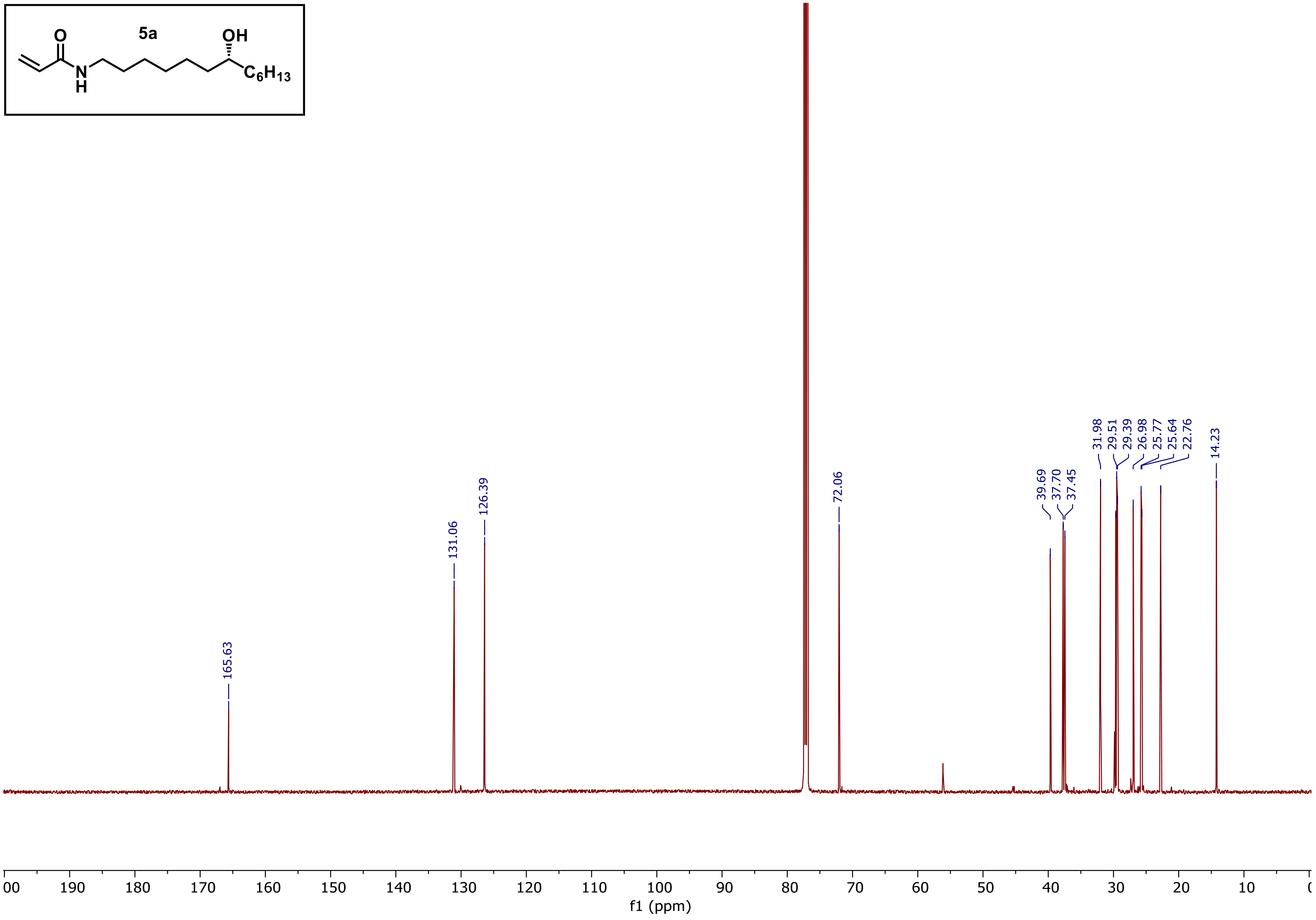
5a
OH
O
N
H
C6H13
165.63
131.06
126.39
72.06
39.69
37.70
37.45
31.98
29.51
29.39
26.98
25.77
25.64
22.76
14.23
00
190
180
170
160
150
140
130
120
110
100
90
80
70
60
50
40
30
20
10
f1 (ppm)

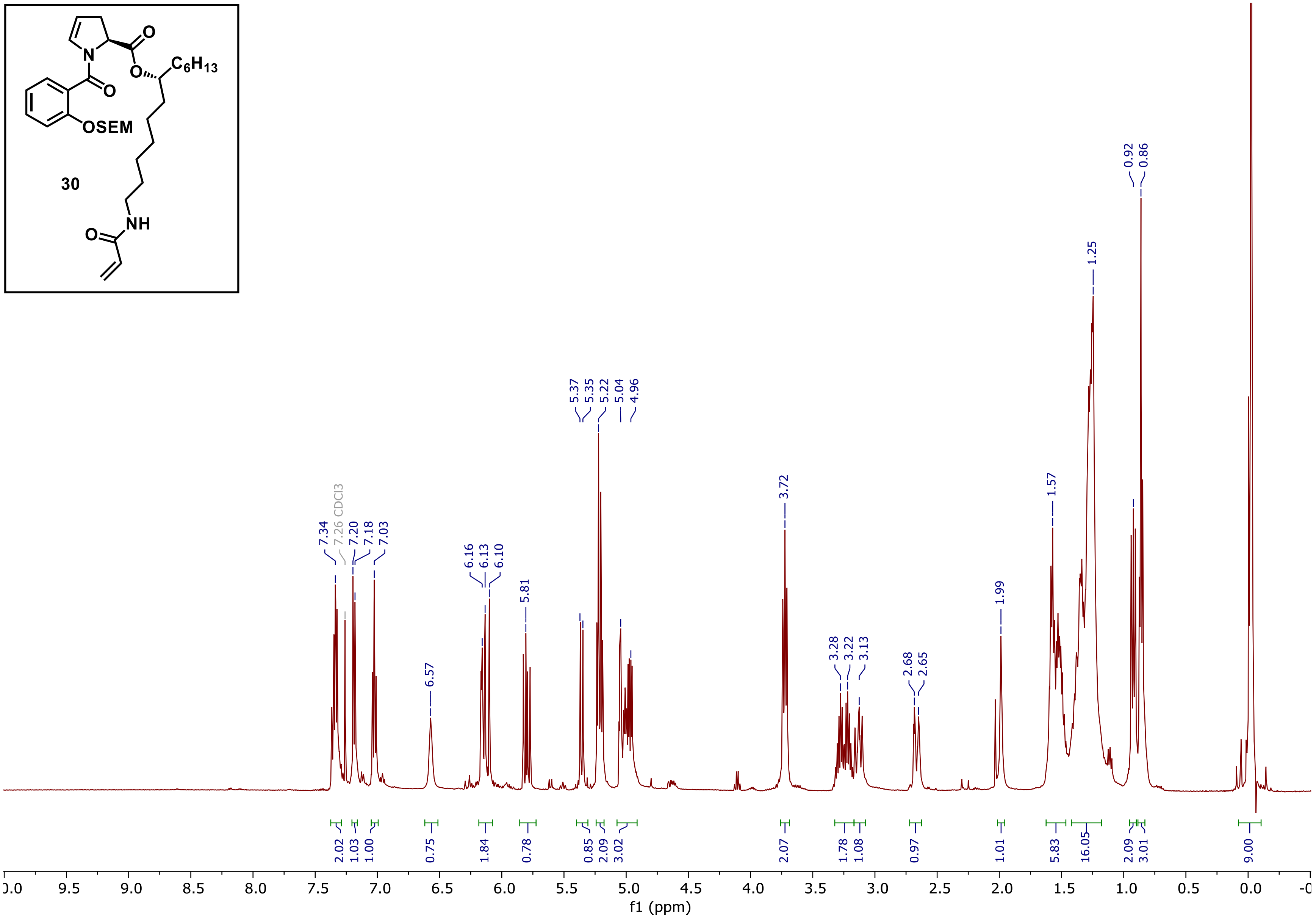

OSEM
C6H13
30
NH
7.34
7.26 CDCl3
7.20
7.18
7.03
6.57
6.16
6.13
6.10
5.81
5.37
5.35
5.22
5.04
4.96
3.72
3.28
3.22
3.13
2.68
2.65
1.99
1.57
1.25
0.92
0.86
2.02
1.03
1.00
0.75
1.84
0.78
0.85
2.09
3.02
2.07
1.78
1.08
0.97
1.01
5.83
16.05
2.09
3.01
9.00
f1 (ppm)

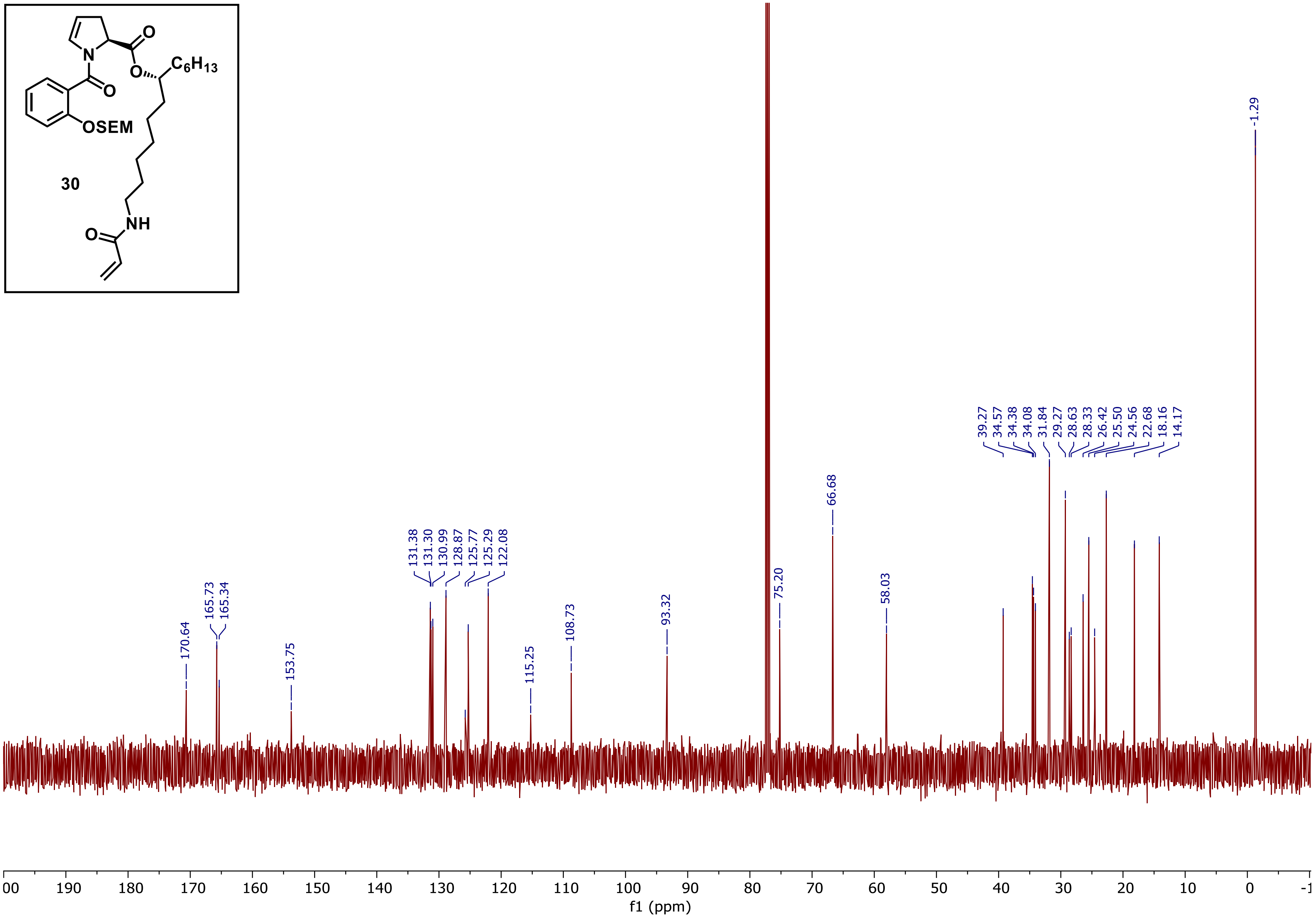
N
O
O
C6H13
O
OSEM
30
NH
O
170.64
165.73
165.34
153.75
131.38
131.30
130.99
128.87
125.77
125.29
122.08
115.25
108.73
93.32
75.20
66.68
58.03
39.27
34.57
34.38
34.08
31.84
29.27
28.63
28.33
26.42
25.50
24.56
22.68
18.16
14.17
-1.29
f1 (ppm)

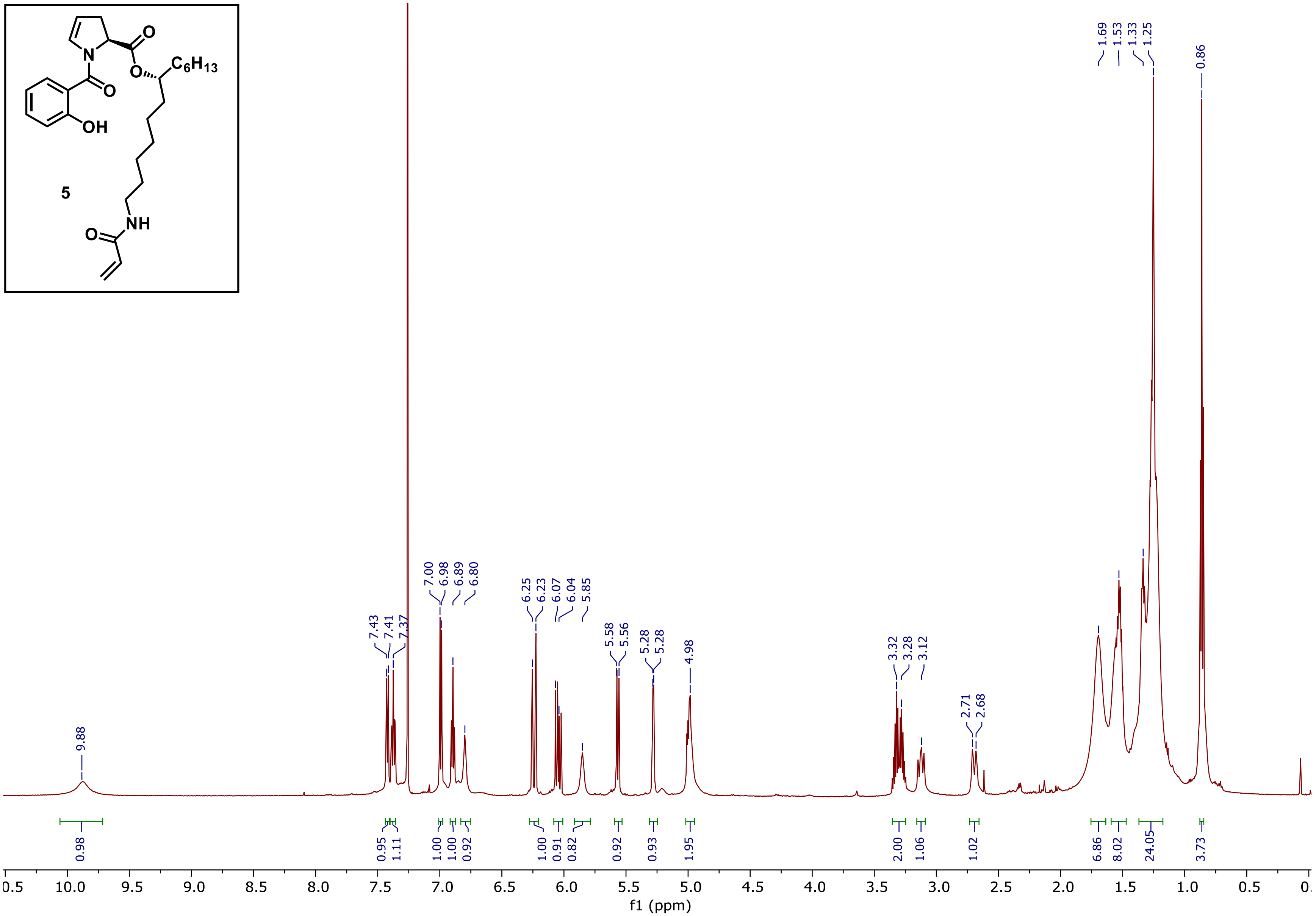

C6H13
OH
NH
O
N
5
9.88
7.43
7.41
7.37
7.00
6.98
6.89
6.80
6.25
6.23
6.07
6.04
5.85
5.58
5.56
5.28
5.28
4.98
3.32
3.28
3.12
2.71
2.68
1.69
1.53
1.33
1.25
0.86
0.98
0.95
1.11
1.00
1.00
0.92
1.00
0.91
0.82
0.92
0.93
1.95
2.00
1.06
1.02
6.86
8.02
24.05
3.73
f1 (ppm)

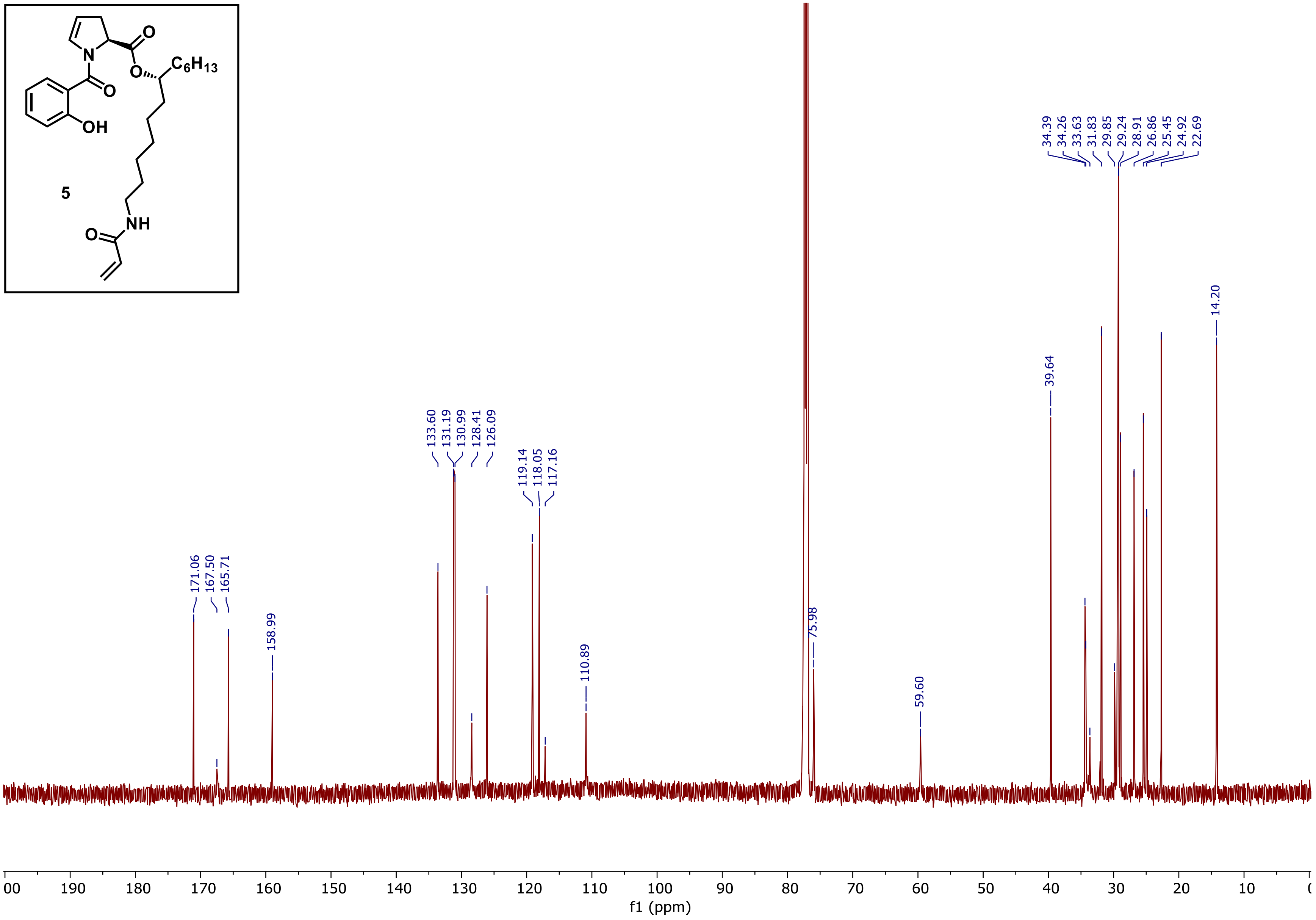

O
N
O
O
C_6H_{13}
OH
5
NH
O
171.06
167.50
165.71
158.99
133.60
131.19
130.99
128.41
126.09
119.14
118.05
117.16
110.89
75.98
59.60
39.64
34.39
34.26
33.63
31.83
29.85
29.24
28.91
26.86
25.45
24.92
22.69
14.20
f1 (ppm)
